# Genetic Characterization of a Novel North American-Origin Avian Influenza A (H6N5) Virus Isolated from Bean Goose of South Korea in 2018

**DOI:** 10.3390/v12070774

**Published:** 2020-07-17

**Authors:** Ngoc Minh Nguyen, Haan Woo Sung, Ki-Jung Yun, Hyun Park, Seon-Ju Yeo

**Affiliations:** 1 Zoonosis Research Center, Department of Infection Biology, School of Medicine, Wonkwang University, Iksan 570-749, Korea; minhngoc90@wku.ac.kr; 2 College of Veterinary Medicine, Kangwon National University, Chuncheon 200-701, Korea; sunghw@kangwon.ac.kr; 3 Department of Pathology, School of Medicine, Wonkwang University, Iksan 570-749, Korea; kjyun@wku.ac.kr

**Keywords:** intercontinental transmission virus isolate, H6N5, South Korea, North American-origin, bean goose

## Abstract

The complex overlap in waterfowl migratory pathways across the world has established numerous occurrences of genetic reassortment and intercontinental spread of avian influenza virus (AIV) over long distances, thereby calling for huge efforts and targeted surveillance for infection control. During annual surveillance in South Korea in 2018, a novel avian influenza H6N5 (K6) subtype was isolated from the fecal sample of wild bird. Genomic characterization using a phylogenetic tree indicated the K6 virus to be of North American-origin, with partial homology to an H6N5 strain, A/Aix galericulata/South Korea/K17-1638-5/2017 (K17). A monobasic residue at the HA cleavage site and absence of a notable mutation at the HA receptor-binding site suggested the isolate to be of low pathogenicity. However, molecular analysis revealed the E119V mutation in the NA gene and a human host marker mutation E382D in the polymerase acidic (PA) gene, implying their susceptibility to neuraminidase inhibitors and potential infectivity in humans, respectively. For comparison, K6 and K17 were found to be dissimilar for various mutations, such as A274T of PB2, S375N/T of PB1, or V105M of NP, each concerning the increased virulence of K6 in mammalian system. Moreover, kinetic data presented the highest viral titer of this H6N5 isolate at 10^6.37^ log_10_TCID_50_ after 48 h of infection, thus proving efficient adaptability for replication in a mammalian system in vitro. The mouse virus challenge study showed insignificant influence on the total body weight, while viral load shedding in lungs peaked at 1.88 ± 0.21 log_10_ TICD_50_/mL, six days post infection. The intercontinental transmission of viruses from North America may continuously be present in Korea, thereby providing constant opportunities for virus reassortment with local resident AIVs; these results hint at the increased potential risk of host jumping capabilities of the new isolates. Our findings reinforce the demand for regular surveillance, not only in Korea but also along the flyways in Alaska.

## 1. Introduction

Based on surface glycoproteins, avian influenza virus (AIV) has been divided into subtypes including 18 HA and 11 NA [1], which are further distributed based on their geographical separation, into the Eurasian and American lineages [2]. However, despite the physical barriers, intercontinental circulation and reassortment of these ancestries have been frequently observed.

The main reason underlying viral dispersal over long distances is likely the overlap of flyways of wild migratory birds, which happen to be the major hosts for AIV. Particularly, four flyways (East Asian–Australasian, West Pacific, Pacific Americas, and Mississippi Americas) intersect at the boundary of the Eastern and Western hemispheres, near Alaska, causing an extensive diversity of Eurasian and North American bird species to be found in this area during spring and fall migrations [3]. Certain key vector bird species, such as green-winged teal (*Anas crecca*), mallard (*Anas platyrhynchos*), and northern pintails (*Anas acuta*), have been reported to carry viruses containing genetic divergence of both Asian and North American species, showing a high prevalence of group HA-1, 3, 4, 5, 6 and NA-1, 2, 6, 8 during the 2006–2008 surveillance in Alaska [4,5,6].

South Korea has recently recorded converse intercontinental exchanges from the North American lineage of the nonstructural (NS) gene segment in several subtypes (H1N1, H1N8, H4N6, H6N1, H6N2, and H6N8) during the surveillance from 2012 to 2017, as a consequence of wild bird (greater white-fronted geese) movement during summer and winter, thereby suggesting the introduction of a gene from American-origin AIV in Korea [7].

In a recent publication, an H6N5 strain, A/Aix galericulata/South Korea/K17-1638-5/2017(H6N5) (designated as K17) was isolated from the feces of a mandarin duck during July 2017–March 2018. This virus carried the full gene segment from a North American ancestor [8]. However, mandarin ducks (*Aix galericulata*) belong to the Asian population that is primarily found in Southeast China, and, therefore, does not regularly participate in the transfer between Eurasia and North America; hence, there would likely be alternative wild birds involved in the transmission of H6N5 to the mandarin ducks in Korea [8]. Frequently, the H6 subtype has been recorded as the cause of widespread outbreaks in poultry and may also sporadically infect humans [9,10,11]. Previously, an unconventional H6N5 strain in Korea had been characterized as a novel pathogenic AIV, capable of infecting mammalian species, with no evidence of adaptation [12]; this suggested the potential risk of cross-species, although the isolate was confirmed to contain all gene segments from Eurasian ancestry.

The impact of foreign genes imported to the local population of AIV H6 in Korea has been rarely described. Therefore, we aimed to emphasize the need for continued surveillance and characterization of H6 outbreaks. In this study, a novel H6N5 strain introduced into Korea from North America was investigated for replicability, pathogenicity, and toxicity in mice. Specifically, the analysis based on satellite tracking devices attached to host species provided valuable information to understand how North American-origin H6N5 could spread in Korea.

## 2. Materials and Methods

### 2.1. Sample Collection

Fresh feces of wild birds in South Korea were collected using sterile swabs in March 2018. The collected samples were stored at 2–8 °C, before being shipped to the laboratory within 12 h, for further analysis.

### 2.2. Virus Isolation from the Feces

As described previously [13], the fecal samples were resuspended in phosphate-buffered saline (pH 7.4; supplemented with 100 U/μL penicillin and 100 mg/μL streptomycin), thoroughly mixed by vortexing, and centrifugated at 3000 rpm for 10 min at 4 °C. The supernatants were reserved for subsequent filtering (0.45-µm pore size; GVS Syringe, Nova-Tech, Kingwood, TX, USA), and then inoculated into the allantoic cavities of specific pathogen-free 10-day-old embryonated chicken eggs (Seng-Jin Inc., Eumsung, Korea) for 72 h at 37 °C, under humidified conditions, eventually chilling them at 4 °C overnight. The presence of influenza virus in allantoic fluids of the inoculated eggs was first verified using an HA assay [14].

### 2.3. RNA Extraction and Bird Identification

Viral RNA was extracted directly from confirmed-HA-positive samples using the RNeasy Mini kit (Macherey-Nagel, Düren, Germany) according to the manufacturer’s instructions. RNA was eluted in 60 μL of RNase-free water, supplied with 20 units of RNase inhibitor for storage at −80 °C. To verify that the samples contained Influenza A viruses, reverse transcription polymerase chain reaction (RT-PCR) was performed to amplify the matrix gene, following the World Health Organization guideline, using universal primers and probes [15]. Bird identification was conducted via barcoding technique, using primers for identifying the mitochondrial cytochrome oxidase (COI) gene in DNA isolated from the fecal samples, as described previously [16,17].

### 2.4. Next-Generation Sequencing (NGS) by Illumina HiSeq X Method

NGS was conducted by GnCBio (Daejeon, Korea), following the HiSeq X method, as reported previously [13,18]. Briefly, influenza viral RNA was detected using an RNA 6000 pico kit (Agilent Technologies, Santa Clara, CA, USA), and RNA concentration was measured with a spectrophotometer. A cDNA library of influenza viral RNA was prepared using the QIAseq FX single cell RNA library kit (Qiagen, Hilden, Germany). The library concentration was measured by LightCycler qPCR (Roche, Basel, Switzerland), and library size was checked using TapeStation HS D5000 ScreenTape (Agilent Technologies, Santa Clara, CA, USA. For cluster generation, the library was loaded into a flow cell, in which fragments were captured on a lawn of surface-bound oligos complementary to the library adapters. Each fragment was then amplified into distinct clonal clusters through bridge amplification. When cluster generation was complete, the templates were used for sequencing. Illumina SBS technology utilizes a proprietary reversible terminator-based method that detects single bases as they become incorporated into DNA template strands. Since all four reversible, terminator-bound dNTPs were present during each sequencing cycle, natural competition minimized their incorporation bias and greatly reduced raw error rates, compared to that in other technologies. Results provided highly accurate base-by-base sequencing that virtually eliminated sequence-context-specific errors, even within repetitive sequence regions and homopolymers. Sequencing data was then converted into raw data for analysis.

### 2.5. Phylogenetic Analysis

Reference sequences were downloaded from both the NCBI-Influenza Virus Resource (https://www.ncbi.nlm.nih.gov/genbank/) and Global Initiative on Sharing All Influenza Data (GISAID; http://www.gisaid.org). Nucleotide homology analysis was compared by BLAST search of the NCBI and/or GISAID databases. The phylogenetic relationships with reference sequences of eight gene segments (polymerase basic 2 (PB2): 2313 nucleotides (nt), polymerase basic 1 (PB1): 2288 nt, polymerase acidic (PA): 2199 nt, HA: 1705 nt, nucleoprotein (NP): 1532 nt, neuraminidase (NA): 1425 nt, matrix (M): 1001 nt, and NS: 864 nt) were determined by alignment using MUSCLE and edited with Molecular Evolutionary Genetics Analysis Version 6.0 (MEGA6) [19]. Rooted phylograms were prepared by the maximum likelihood method with 1000 bootstrap replicates for each gene using MEGA6 software.

### 2.6. Viral Replication Kinetics in MDCK Cells

To examine the viral replication capacity in mammalian cells, an isolated H6N5 (A/Bean goose/South Korea/KNU18-6/2018(H6N5)) virus, along with reference strains: human-origin H1N1 (A/California/04/2009 (H1N1)), H7N1 (Youngsoori-12-2F4(2012)), and chicken-origin H9N2 (A/chicken/Korea/KNUSWR09/2009 (H9N2)) were infected into Madin-Darby canine kidney (MDCK; obtained from American Type Culture Collection) cells. Briefly, the selected viruses were inoculated into MDCK monolayers at a multiplicity of infection (MOI) of 0.01. The inoculated cells were incubated with DMEM containing 1 µg/mL TPCK-treated trypsin (Sigma-Aldrich, St Louis, MO, USA) at 37 °C. The supernatant was collected at different time points: 12, 24, 36, 48, 60, and 72 h postinfection (hpi) [9]. Viral replication titers were determined by TCID_50_ assay.

### 2.7. TCID_50_—50% Tissue Culture Infectious Dose and EID_50_—50% Egg Infectious Dose

The TCID_50_ titers were determined by enzyme-linked immunosorbent assay (ELISA), as reported previously [20]. Briefly, MDCK cells were grown on flat-bottom 96-well plates and washed with phosphate-buffered saline. Thereafter, the cells were inoculated with 10-fold serial dilutions of virus samples. Inoculated cells were incubated at 37 °C and 5% CO_2_. After 3 days, the medium was removed from microtiter plates, and the cells were washed with PBS, followed by fixation with 80% cold acetone. ELISA was performed and TCID_50_ titers were calculated via the Reed and Muench method [21]. To measure the EID_50_ titer, 10-fold serial dilutions of the virus were prepared, and 100 μL of each dilution was inoculated into the chorio-allantoic cavities of 10-day-old specific-pathogen-free embryonated chicken eggs (Seong-Jin Inc., Eumsung, Korea). The eggs were incubated at 37 °C for 3 days. Five eggs were infected with each viral dilution. Harvested allantoic fluid was then tested for hemagglutination (HA) activity and EID_50_ was determined [22].

### 2.8. Animal Study

Six-week-old female BALB/c mice were purchased from Orient Bio (Seongnam, Gyeonggi, Korea). Each group of 16 mice was inoculated intranasally (i.n.) with 10^5^ EID_50_/50 μL of K6-H6N5, H1N1, or H7N1 strains [12]. For the K6-H6N5 group, 10^5^, 10^6^, and 10^7^ EID_50_/50 μL were inoculated. Anesthesia was conducted with isoflurane, following manufacturer’s instruction (Hana Pharm Co. Ltd., Hwasung, Korea) under the guidelines of vertebrate animal research, the University of Iowa [23]. Three mice per group were sacrificed at 3, 6, and 14 days post infection (dpi), and the virus titer in lung was determined thereafter. Ten mice per group were monitored for weight change, morbidity, and mortality, each day for 14 days post infection. This study was approved by the Animal Ethics Committee of the Wonkwang University (WKU19-64), December 19, 2019 and all methods were performed in accordance with the relevant guidelines and regulations. At 6 dpi, lungs from three mice were placed in 10% formalin/saline for studying pathology. Tissues in 10% formalin/PBS were processed and embedded in paraffin. Lung sections were cut at 4–5 μm from paraffin embedded lung tissues and mounted on glass slides. Histological characterization was analyzed using standard hematoxylin and eosin (H&E) staining and light microscopy (magnification ×100).

### 2.9. Statistics

The mean, SD, and Student’s *t*-test were conducted using GraphPad Prism version 5.0. Results were presented as the mean ± SD. A value of *p* < 0.05 was considered significant. Two-way or one-way analysis of variance (ANOVA) was used to analyze viral replication kinetics in MDCK cells and compare lung weight of viruses infected mouse, respectively.

## 3. Results

### 3.1. Genetic Characterization of Novel Avian Influenza A (H6N5) in 2018

In March 2018, fresh fecal samples from wild birds were collected from Gyeonggi-do (37° 45′28.35″N, 126° 40′ 8.60″E), South Korea. Full-length genomes of the isolates were investigated by NGS; consequently, the H6N5 isolate, A/Bean goose/South Korea/KNU18-6/2018(H6N5) (abbreviated as K6), was selected for genomic characterization and deposited in GenBank (Table 1). Bean goose was identified as the bird species carrying the H6N5 virus, based on the COI gene sequence, as shown in Figure S1.

All gene segments of K6, isolated from bean goose in March 2018, shared ≥ 99% nucleotide identity with those of ≥ 1 isolates recovered from wild and domestic birds sampled in North America during 2007–2018 (Table S1). According to BLAST results, five of the eight gene segments (PB2, PA, HA, NA, and NS) for the K6 isolate shared ≥ 99% nucleotide identity with those of K17 (Table 1). In contrast, three internal genes (PB1, NP, and MP) of K6 and K17 shared ≤ 99% nucleotide identity and possessed ≥ 99% nucleotide identity with other strains in North America, implying K6 and K17 to be partially identical (Table 1).

**Figure 1:**
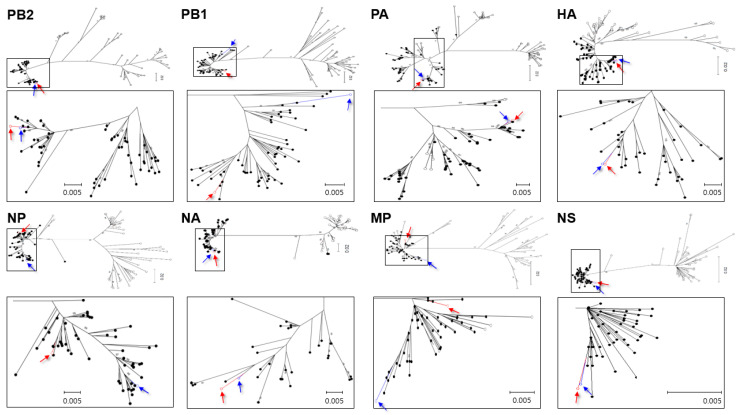
Maximum likelihood phylogenetic trees showing inferred relationships among nucleotide sequences for the complete coding regions of K6 and K17 gene segments. Red open circle indicates K6-H6N5 and blue open circle indicates K17. These are highlighted with red and blue arrows used for K6 and K17, respectively. Closed and open circles indicate lineages of North America and Eurasia, respectively.

The phylogenetic tree strongly supported the structuring of major clades by continental affiliation of reference strains. Sequence information for all eight gene segments of K6 clustered within the clades composed of reference sequences for AIV originating from samples collected in North America (Figure 1 and Figure S2 and S3).

Based on the phylogenetic analysis, both K6 and K17 belonged to North American strains; specifically, the HA and NA genes may have originated from the same AIV ancestor (A/northern pintail/California/2015 (H6N5)). We, therefore, provided evidence to support the North American ancestry of K6, along with a close relationship with K17. Our results may also provide evidence for the introduction of H6N5, of North American-origin, into Korea via bean geese, based on the previous evidence for the introduction of K17 into Korea by mandarin ducks.

### 3.2. Molecular Characterization of H6N5 Isolate

Acquisition of multiple basic amino acids at the HA cleavage site and/or additional changes in amino acids in the HA receptor-binding site, or in other gene segments, have been involved in the evolution of an LPAI virus into HPAI [24,25]. Table 2 shows the comparison of several well-known mutations in gene segments between our isolate (K6-2018) and reference strains isolated in Korea: K17-2017 (A/Aix galericulata/South Korea/K17-1638-5/2017 (H6N5)), CN5-2009 (A/aquatic bird/Korea/CN5/2009 (H6N5)), and W69-2005 (A/aquatic bird/Korea/W69/2005(H6N5)), together with an H6 infected-human isolate: TW02/2013: A/Taiwan/2/2013 (H6N1) human infected isolate.

The monobasic residue at the HA cleavage site, with no notable mutation at the HA receptor-binding site, indicated our H6N5 isolate and reference strains to be LPAI (Table 2). Additionally, the NAs of the H6N5 isolates had no amino acid deletion in the stalk region [26]. However, the E119V mutation in NA was detected in all H6N5 isolates, suggesting their susceptibility to neuraminidase inhibitors, such as Oseltamivir, Peramivir, and Zanamivir [27]. Mutations in internal genes, also presented in Table 2, might imply an increase in viral replication efficiency, along with virulence, in mammals. Particularly, one human host marker mutation, E382D, in the PA gene was observed in two new strains imported from North America (K6 and K17), although they were not displayed in two old H6N5 isolates from Korea (CN5 and W69), which had originated from Eurasia. K6 and K17 were variable in certain molecular aspects, such as mutation at A274T of PB2, S375N/T of PB1, or V105M of NP, each contributing to the potentially increased virulence of K6 in mammals (Table 2).

**Table 2:** Molecular characteristics of influenza A (H6N5) isolates used in this study.

Viral Protein	Amino Acid	K6-2018	K17-2017	CN5-2009	W69-2005	TW02/2013	Comments	Reference
HA *	Cleavage sites	PQIETR↓GLF	PQIETR↓GLF	PQIETR↓GLF	PQIETR↓GLF	PQIATR↓GLF	Monobasic-LPAI	[24]
HA *-Receptor-binding site	A138S	A	A	A	A	A	Switch the preference from avian- to human-type receptors	[25]
	E190D	E	E	E	E	V	Switch the preference from avian- to human-type receptors	[25]
	G225D	G	G	G	G	G	Switch the preference from avian- to human-type receptors	[25]
	Q226L	Q	Q	Q	Q	Q	Switch the preference from avian- to human-type receptors	[25]
	G228S	G	G	G	G	S	Switch the preference from avian- to human-type receptors	[25]
HA*	L102F	L	L	L	L	L	Increased pathogenicity in mice	[28]
	T/E173G/D/V	T	T	N	N	T	Increased virus binding to α-2,6-linked sialic acid	[28]
	N379T	N	N	N	N	N	Increased virulence in mammals	[29]
NA	Amino acid deletion at stalk region	No	No	No	No	Yes	Increased virulence in mice	[26]
	E119V	V	V	V	V	I	Reduced inhibition by OS (Oseltamivir) and PER (Peramivir) and highly reduced inhibition by ZA (Zanamivir)	[21]
PB2	T63I (with PB1 M677T)	I	I	I	I	I	Pathogenic in mice	[30]
	L89V	V	V	V	V	V	Enhanced polymerase activity, increased virulence in mice	[31]
	K251R	R	R	R	R	K	Increased virulence in mice	[32]
	A274T	T	A	A	A	A	Increased polymerase activity, increased virulence in mammals and birds	[33]
	G309D	D	D	D	D	D	Enhanced polymerase activity, increased virulence in mice	[31]
	T339K	K	K	K	K	K	Enhanced polymerase activity, increased virulence in mice	[31]
	Q368R	R	R	R	R	R	Increased polymerase activity, increased virulence in mammals	[34,35]
	H447Q	Q	Q	Q	Q	Q	Increased polymerase activity, increased virulence in mammals	[34,35]
	R477G	G	G	G	G	G	Enhanced polymerase activity, increased virulence in mice	[31]
	I495V	V	V	V	V	V	Enhanced polymerase activity, increased virulence in mice	[31]
	A676T	T	T	T	T	V	Enhanced polymerase active, increased virulence in mice	[31]
	D701N	D	D	N	D	D	Increased polymerase activity, increased virulence in mammals, mammalian host marker	[36,37,38]
PB1	D/A3V	V	V	V	V	V	Increased polymerase activity, increased virulence in mammals	[34,35]
	L13P	P	P	P	P	P	Increased polymerase activity, increased virulence in mammals, mammalian host marker	[36,37]
	R207K	K	K	K	K	K	Increased polymerase activity in mammalian cells	[39]
	K328N	N	N	N	N	S	Increased polymerase activity, increased virulence in mammals	[34,35]
	S375N/T	N	S	N	N	N	Increased polymerase activity, increased virulence in mammals, human host marker	[34,35,40]
	H436Y	Y	Y	Y	Y	Y	Increased polymerase activity and virulence in mallards, ferrets, and mice	[39]
	A469T (with NS1 N205K; NEP T48N)	T	T	T	T	T	Conferred in contact transmissibility in guinea pigs	[41]
	L473V	V	V	V	V	V	Increased polymerase activity and replication efficiency	[42]
	V652A	A	A	A	A	A	Increased virulence in mice	[32]
	M677T (with PB2 T63I)	T	T	T	T	T	Pathogenic in mice	[30]
PB1-F2	N66S	N	S	N	N	Truncated	Increased virulence in mammals	[43,44]
PA	S37A	A	A	A	A	A	Significantly increased viral growth and polymerase activity in mammalian cells	[45]
	H266R	R	R	R	R	R	Increased polymerase activity, increased virulence in mammals and birds	[33]
	F277S	S	S	S	S	S	Adapt to mammalian hosts	[46]
	C278Q	Q	Q	Q	Q	Q	Adapt to mammalian hosts	[46]
	E382D	D	D	E	E	E	Human host marker	[40,47]
	S/A515T	T	T	T	T	T	Increased polymerase activity, increased virulence in mammals and birds	[33,39]
	L653P	S	P	P	P	P	Adapt to mammalian hosts	[46]
NP	V41I	I	I	I	I	I	Might contribute to viral transmissibility	[48]
	V105M	M	V	M	I	I	Contribute to the increased virulence	[49]
	F253I	I	I	I	I	I	Results in attenuated pathogenicity of the virus in mice	[50]
	I353V	V	V	V	V	V	Increased virulence in mice	[32]
MP	N30D	D	D	D	D	D	Increased virulence in mammals	[51]
	T215A	A	A	A	A	A	Increased virulence in mammals	[52]
NS1	A/P42S	S	S	S	S	S	Increased virulence in mammals, antagonism of IFN induction	[52,53]
	T/D/V/R/A127N	N	N	N	N	N	Increased virulence in mammals	[54]
	V149A	A	A	A	A	A	Pathogenicity in mice, antagonism of IFN induction	[55]

* H3 numbering. K6-2018: A/Bean goose/South Korea/KNU18-6/2018(H6N5) K17-2017: A/Aix galericulata/South Korea/K17-1638-5/2017 (H6N5) CN5-2009: A/aquatic bird/Korea/CN5/2009 (H6N5) W69-2005: A/aquatic bird/Korea/W69/2005 (H6N5) TW02/2013: A/Taiwan/2/2013 (H6N1) infected-human isolates. The letters are amino acid abbreviation. A, Alanine; E, Glutamate; G, Glycine; Q, Glutamine; L, Leucine; T, Threonine; N, Asparagine; I, Isoleucine; V, Valine.

### 3.3. Viral Replication Kinetics in MDCK Cells

**Figure 2:**
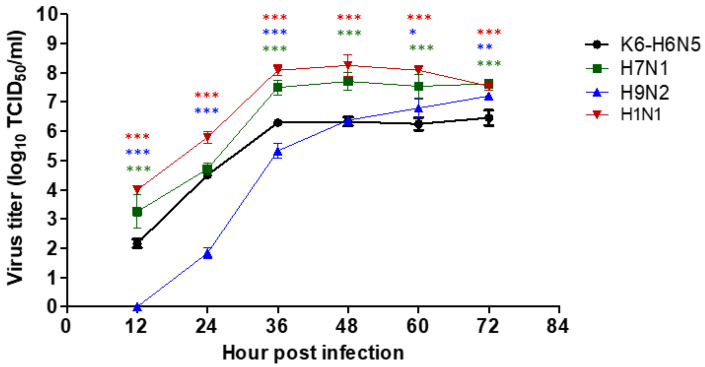
In vitro growth properties of H6N5 in MDCK cells. Viruses were inoculated into MDCK monolayers at a multiplicity of infection (MOI) of 0.01. The supernatants were collected at different time points: 12, 24, 36, 48, 60, and 72 hours post infection (hpi). Viral replication titers were determined by TCID_50_ assay. *, *p* < 0.05; **, *p* < 0.01; ***, *p* < 0.001.

To address the potentially increased virulence of the novel H6N5 originating from North America, the viral replication capability of our isolate in mammalian cells was investigated relative to that of H1N1, H7N1, and H9N2 strains in vitro (Figure 2).

Results showed that the K6 virus grew efficiently and reached a peak at 48 hpi. The same kinetic curve pattern was observed in the control H7N1 strain. However, the titer of the H7N1 virus was approximately 10–30-fold higher than that of H6N5 at 36 hpi. In contrast, the H9N2 virus grew slowly and showed the lowest titer from the beginning to 12, 24, and 36 hpi. Raw data for the TCID_50_ assay are presented in Figure S4.

### 3.4. Animal Study

**Figure 3:**
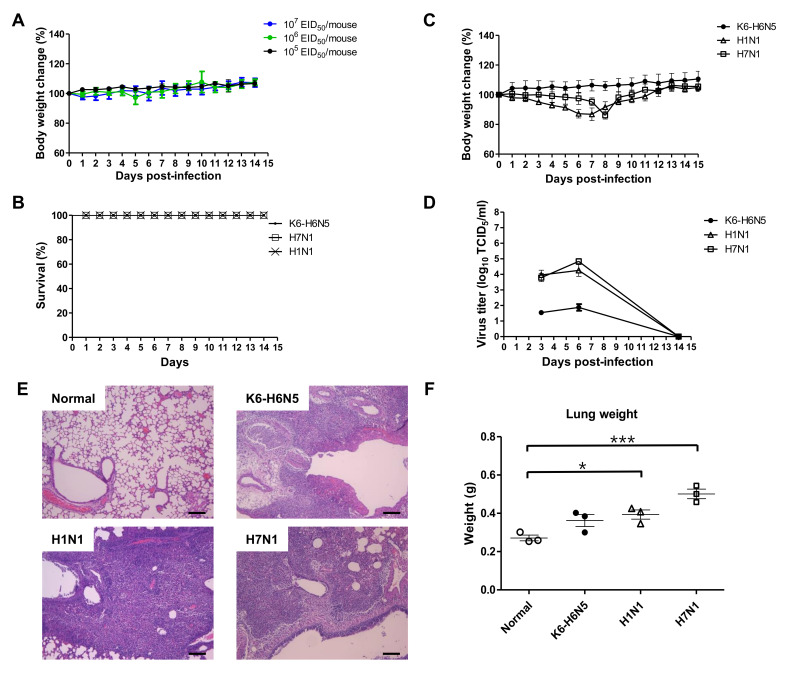
Pathogenicity of K6-H6N5 virus in vivo. (**A**) Balb/c mice were intranasally challenged with different titers of the K6-H6N5 isolate. (**B**–**F**) H1N1 and H7N1 strains were used as controls, administered intranasally with 10^5^ EID_50_. Mouse weight (**B**) and survival rate (**C**) were observed for 14 dpi. Body weight is presented as % of those of original mice (*n* = 10). (**D**) Mean viral titers in the lungs of mice (*n* = 3) were measured at 3, 6, and 14 dpi. (**E**,**F**) Histology of lung inflammation following virus infection was observed. (**E**) H&E staining of uninfected control (normal); H6N5; H1N1; H7N1-infected mouse lungs at 6 dpi (scale bar, 100 μm; original magnification, 100 ×). (**F**) Mean of lungs weight from an uninfected control (normal) and H1N1-, H7N1-, and H6N5-infected mice at 6 dpi (*n* = 3). *, *p* < 0.05; ***, *p* < 0.001

To determine the pathogenic potential of the new isolate in mammals, 10^5^,10^6^, and 10^7^ EID_50_/ 50 µl of H6N5 viruses were inoculated into six-week-old female BALB/c mice. Overall, the infected mice displayed no severe clinical signs, including ruffled fur, depression, labored breathing, and/or severe weight loss during the 14 days post infection (dpi). Although increasing titers of the H6N5 virus infected to the mice from 10^5^ to 10^6^ to 10^7^ EID_50_, it did cause insignificant changes in the total body weight during the 14 dpi period (Figure 3A).

For comparison, control H1N1 and H7N1 strains were inoculated at 10^5^ EID_50_ /mouse. The body weight of the mice infected with these viruses was found to decrease regularly, with the lowest weight (86.81% and 86.08%) observed at approximately day 7–8 post challenge for H1N1 and H7N1, respectively. Meanwhile, the group infected with the K6-H6N5 isolate, retained stable bodyweight throughout the 14 days. Moreover, from day 9 the mice infected with all three strains recovered their body weight (Figure 3B). As a result, no significant differences were observed between the groups of mice in survival rate (Figure 3C). The viral load shedding in lungs after 3, 6, and 14 dpi has been represented in Figure 3D and Figure S5. K6-H6N5 replicated in the lung with the lowest titer (1.54 ± 0.08 log_10_ TICD_50_/mL) at 3 dpi, which then increased slightly to 1.88 ± 0.21 log_10_ TICD_50_/mL at 6 dpi, and was absent at 14 dpi; whereas H1N1 and H7N1 were maintained at a comparatively high titer at 3 dpi (3.96 ± 0.31; 3.79 ± 0.26 log_10_ TICD_50_/mL, respectively) and at 6 dpi (4.25 ± 0.37; 4.84 ± 0.19 log_10_ TICD_50_/mL, respectively). On day 14, there was no viral shedding in the lungs of all groups.

The mouse infected with K6 did not show any considerable change in the body weight or the survival rate; however, it exhibits the viral shedding in mice lungs of those at 3; 6 dpi, the lung histopathology of infected mice were examined. Hematoxylin and eosin (H&E) stained section of the K6, H7N1, H1N1-infected lung at 6 dpi showing an extensive penetrate of neutrophils that fill the alveolar air spaces (Figure 3E). K6-H6N5 virus-infected lungs presented cellular consolidation compared with those of the normal (uninfected) mice at 6 dpi (Figure S6) although lungs of K6-H6N5-infected mice did not show a significant increase in weight whereas those of H7N1- or H1N1-infection increased a significant increase of lung weight (Figure 3F). Taken together, it indicated that K6-H6N5 has a relatively lower adaptation to mice compared to H7N1 and H1N1.

### 3.5. Hypothesis for Reassortment Event of A/Bean goose/South Korea/KNU18-6/2018(H6N5)

**Figure 4:**
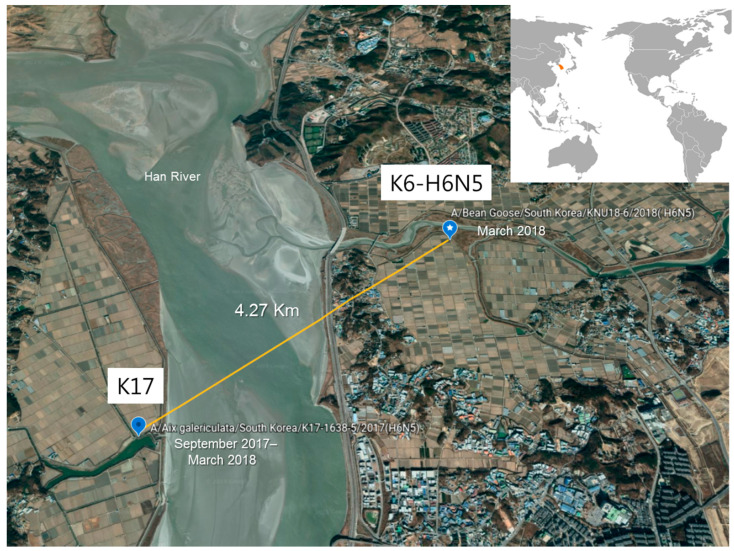
Location of H6N5 isolates in Korea: A/Bean goose/South Korea/KNU18-6/2018(H6N5) and A/Aix galericulata/South Korea/K17-1638-5/2017(H6N5). The map was retrieved from Google Earth.

K17 was identified during September 2017–March 2018 when a K6 strain was isolated in March 2018, and the locations of K17 (37°43′37″ N, 126°39′54″ E) (Figure S7) [8] and K6 were placed within a radius of 4.27 km (Figure 4).

During 2015–2017 in North America, the HA and NA of A/California/H6N5 may have reassorted with other internal gene segments of local AIV residents, thereby transmitting it to Alaska to recruit PB2 for K17, and gathering both PB1 and PB2 to K6 from a donor virus A/Mallard/Alaska/Ah0029066S.2.A/2016 H12N5.

**Figure 5:**
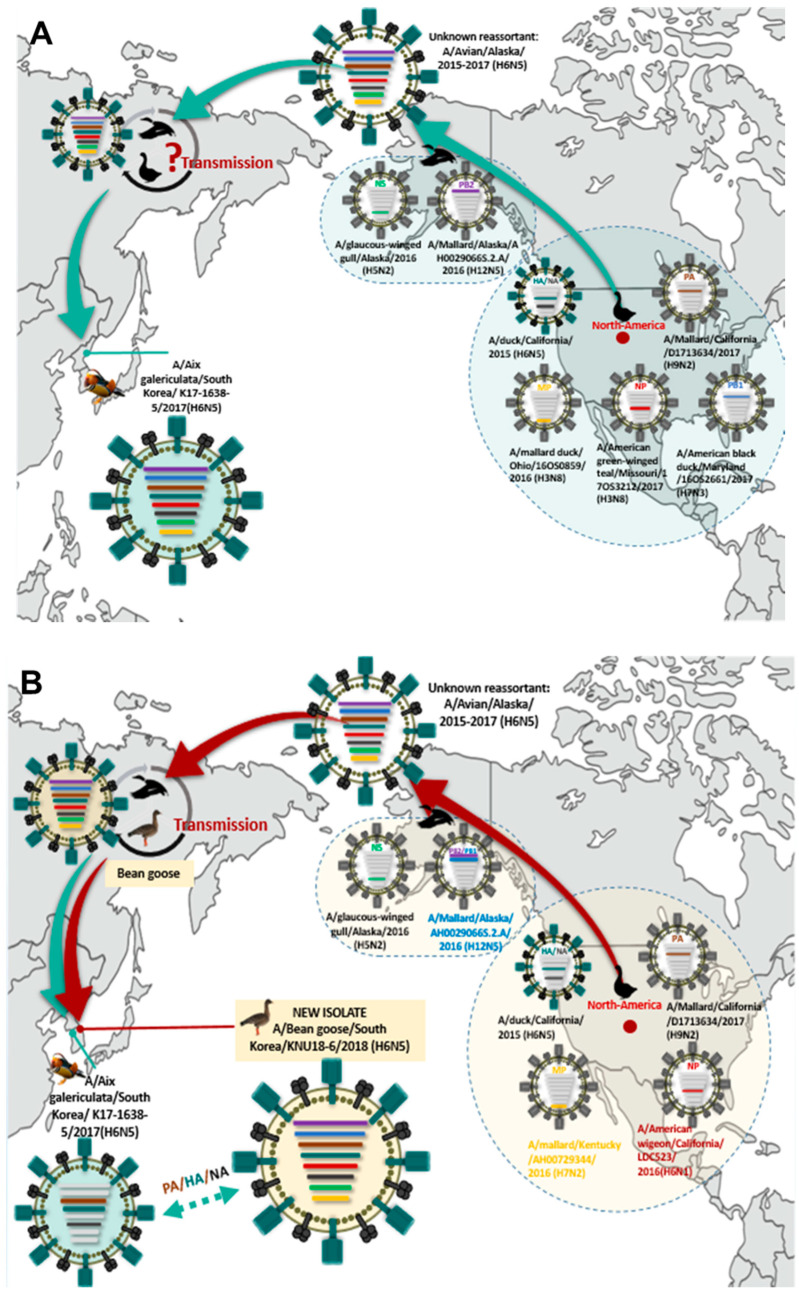
Evolutionary hypothesis for the ancestor of each gene segment of A/Aix galericulata/South Korea/K17-1638-5/2017(H6N5) (**A**) and A/Bean goose/South Korea/KNU18-6/2018(H6N5) (**B**).

The intercontinental transmission may have been facilitated by certain key vector species, such as the northern pintail and mallard, which have wide geographic distribution across Europe, Palearctic, and North America. Somewhere in Russia, near Alaska, the North American-origin H6N5 may have spread to bean geese before being introduced to Korea as K6 (Figure 5B). However, *Aix galericulata* as a host of K17 is an Asian population that breeds, feeds, and overwinters in lowland eastern China and southern Japan. Therefore, K17 has been suggested to be transferred from migratory birds to other native species before spreading to mandarin ducks in Korea (Figure 5A).

## 4. Discussion

The H5 HPAI viruses has been spreading widely across several countries, providing an increasing opportunity for the Asian-origin viruses to be reassorted in the Alaska area before subsequently being transferred to Canada and other states in the USA, rather than being created by multiple reassortments within the local resident LPAI strains. Therefore, the emergence of intercontinental distribution of AI has been tightly monitored to explain the high variations in AIV.

Alaska is a feasible location for intercontinental exchanges, since various AIV of Eurasian and North American lineages have been identified there. Several subtypes, such as H8N4, H9N2, H13, H16, H3, and N7, found in Alaska, contain mixed Eurasian-origin gene segments [56,57,58,59].

The HPAI H5N8 epidemic that occurred during January 2014 in South Korea, infecting 161 commercial poultry flocks, led to the culling of 14,000,000 birds; subsequently, the virus was detected continuously in Germany, England, and the Netherlands in early November 2014. Soon afterward, in early 2015, HPAI H5 had threatened Canada, along with various states in the USA, with more than 200 confirmed cases and 48,000,000 birds affected [60]. Certain HPAI H5 clade 2.3.4.4 viruses isolated from North America were proven to possess the HA gene originating from Asian lineages [61,62].

To date, intercontinental reassortment of several AIV genes in Asian waterfowl has been described in Japan (H4N6) [63] and Korea (H3N8 and H4N6), resulting in the eventual transmission to live animal markets [64]. Moreover, the imported single NS gene segment of AIV from North American to South Korea was recorded between 2012 and 2017 to have occurred via a greater white-fronted goose (*Anser albifrons*) [7], whereas the full K17-H6N5 genome was introduced in 2018 [8].

Phylogenetic tree analysis indicated eight K17 gene segments to share full sequence homology with North American AIV strains, during 2016–2017, rather than with those of Eurasian ancestry (Figure S2). However, the host for K17 was determined to be the mandarin duck [8], which shows narrow distribution in the Far East and Southeast Asia, within the East Asian–Australasian flyway, and is, therefore, not likely to move between the two continents of Eurasia and North America [65].

In contrast, the host of K6 was the bean goose *(Anser fabalis*), which is widely distributed across Asia, Europe, and Russia for both breeding and feeding [65]. Moreover, an overlap area has been identified in Russia, near Alaska, where the migratory birds from North America, such as northern pintail or mallard ducks, share the same distribution as bean goose. Indeed, northern pintails breed across Poland, Russia, Mongolia, Canada, Alaska, and Midwestern United States, and winter primarily at the equator, in Panama, northern sub-Saharan Africa, and tropical South Asia [65]. They have also been detected as the major hosts of several AIV subtypes (H1–8, 10–12, and N1–9), isolated over three years (2006–2008) in Alaska, with seven of their eight gene segments acquired as a result of intercontinental transfer from Asia [6]. Therefore, considering the close habitats of bean geese (host of K6) and mandarin ducks (host of K17) over the same seasonal period in Korea (Figure 4), we hypothesized that the introduction of K17 might be associated with that of K6 to Korea. Although it is possible that K6 and K17 were independently transported, due to the intersection of the K6 and K17 host near Alaska, reassortment may occur during groupings in these similar habitats as well.

This hypothesis was supported by records acquired by the National Institute of Biological Resources from two different satellite tracking devices (br1691 and br1692) monitoring the movement of bean geese during January to November 2017 in Korea. According to the data, in the summer, the bean goose migrated to Russia (near Alaska) from Korea and returned to Korea in November 2017. Furthermore, the migration pattern of the bean goose, with the br1691 device attached, corresponded well with the isolation site identified for K17 during October–November 2017 (Figure S8A), while that of the K6 virus corresponded to the migratory path of the bean goose with br1692 attached, in November 2017, i.e., four months prior to the isolation of K6 in this study (Figure S8B). This data, therefore, supported the theory for possible reassortment of North American-origin virus in Russia, near Alaska, and potential transmission of the K17 virus from a bean goose to mandarin duck during the summer months, before they are returned to Korea, or possible reassortment via cross grouping within Korea (Figure 5B).

To date, gene segments from the novel Eurasian, highly pathogenic, H5 virus in Washington, USA, in 2014, have been found to be genetically similar to that of A/bean goose/Korea/H40/2014 [62]. Therefore, bean geese warrant intensive surveillance for intercontinental spread of Asian- or North American-origin AIV through Beringia in Alaska.

Considering the lack of satellite tracking data for bean geese in Alaska, their migration from North America directly to Asia may still be possible. Bean geese could join other migratory birds and transmit AIV to Eurasia and North America via flyway overlap with wild birds (East Asia, Pacific Americas, and Mississippi Americas), thus indicating a demand for enhancing surveillance in these regions.

The H6 subtype reportedly infects various mammals, including humans. A study examining the antigenic and genetic relatedness of 14 H6 influenza viruses reported efficient replication of an H6 subtype in mice and ferrets without preadaptation [66]. The first report of human infection with a wild H6N1 was from Taiwan [67]. This virus has since been circulating throughout both North America and Eurasia [68]. However, an important G228S (H3 numbering) substitution at the receptor-binding site of HA, as well as a 14-amino acid deletion in the stalk region of NA noted in a human-infecting H6N1 strain, were not identified in the K6 or K17 isolates (Table 2). These results suggest the low pathogenicity of K6 compared to that of the H6 subtype in Taiwan.

Meanwhile, gene reassortment has permitted the H6 AIV to serve as a source of novel HPAI, facilitating the circulation of strains that had previously threatened humans. For instance, during 2014–2016 in China, the novel H5N6 HPAI was reported as lethal in chickens, and moderately pathogenic in mice, leading to several cases of infection and deaths in humans [69,70]. The whole genome of these H5N6 strains exhibited high similarity with those from human isolates and their NA genes originating from H6N6. Thus, it demands the phylogenetic tree analysis, as well as the study replication ability in mammalian systems of new isolates.

According to phylogenetic tree analyses, the previously reported K17 and our current K6 possess a highly similar genome sequence; however, the adaptation in mammals or mice was not characterized for the K17 strain. In our study, K6 showed the highest viral titer of 10^6.37^ log_10_ TCID_50_ at 36–48 hpi, which corresponded with the previous results that had shown H6 viruses to grow efficiently and reach a peak of 10^5^ log_10_ TCID_50_ at 36 hpi in MDCK cells [9]. Moreover, the K6 virus was found to replicate efficiently in lungs without prior adaptation; however, it was not lethal in mice, which is consistent with other publications that examined the toxicity in mice, induced by H6-AIV [71]. The mice challenged intranasally with 10^6^ EID_50_ exhibited slight loss in body weight, and viral load in the lung was found to be 4.82 log_10_ TCID_50_ /100 mg. Alternatively, the A/aquatic bird/Korea/CN5/2009 (H6N5) strain causes illness and lethality in mice, with severe clinical signs, including significant weight loss, ruffled fur, lethargy, and ataxia, with 100% mortality noted within 7 days [12]. According to the molecular analysis of gene segments between K6 and A/aquatic bird/Korea/CN5/2009 (H6N5), the D701N mutation in PB2 may contribute to the increased virulence observed for H6N5 in the mammalian host (Table 2).

Although the K6 or K17 are low pathogenic H6N5 strains imported to Korea with a full genome from North America, they pose a potential risk for increasing pathology in mice and humans via participating in reassortment with local AIV residents. Particularly, the PB2 and/or PA gene of the CN5-2009 strain harboring D701N and L653P substitutions, respectively, can become reassorted with other K6 genes, resulting in potentially increased virulence in mammals. Additionally, K6 may increase the possibility of host jumping by providing the PB2 with A274T mutation or/and the PA gene with E382D alteration to CN5-2009 or other Korea strains reassortment (Table 2).

For comparison, H7N1 and H1N1 were used as positive controls of mouse-adapted strains while H9N2 was a non-mouse-adapted strain. Results showed a kinetic peak for H7N1 at 10^7.7^ log_10_ TCID_50_ at 48 hpi, whereas another publication showed a peak at 10^5^ log_10_ TCID_50_ [72]. Besides, mice challenged with the H7N1 strain displayed 30% weight loss at 8 dpi and only 60% survival by day 15. Meanwhile, our data showed 100% survival in mice with H7N1 at 15 dpi, with only 20% loss in body weight. This difference may be derived from genomic variation of H7 strains.

Overall, our results contributed to furthering the current understanding regarding how North American-origin H6N5 in mandarin duck was introduced into Korea, based on the genetic identity and migration pattern of bean geese during the isolation season of K6 and K17. However, a limitation of our study was that we only suggested the potential import of H6N5 by bean geese to Korean mandarin ducks, considering the database for the spatial migratory patterns of bean geese. Additional influenza surveillance in bean geese, along the flyways in Alaska, might provide useful inferences for early introduction of novel strains into Korea. Further studies should, therefore, focus on collecting direct AIV samples from bean geese with satellite tracking devices attached.

## Figures and Tables

**Table 1:** Sequence homology of the whole A/Bean goose/South Korea/KNU18-6/2018(H6N5) genome.

Gene	GenBank Accession # of K6	Strain Having Highest Nucleotide Identity with K6		Genetic Identity between K6 and K17
		Strain	GenBank Accession # (% Genetic Identity)	
PB2	MN749559	A/Mallard/Alaska/AH0029066S.1.A/2016(H12N5)	MN254630.1 (99.34%)	99.21%
PB1	MN749560	A/Mallard /Alaska/AH0029066S.2.A /2016(H12N5)	MN254483.1 (99.56%)	94.64%
PA	MN749561	A/Aix galericulata/South Korea/K17-1638-5/2017(H6N5)	MK830102.1 (99.49%)	99.49%
HA	MN749562	A/Aix galericulata/South Korea/K17-1638-5/2017(H6N5)	EPI1526581 99.30%	99.30%
NP	MN749563	A/lesser scaup/Wisconsin/17OS5811/2017(H6N1)	EPI1537365 99.67%	96.32%
NA	MN749564	A/Aix galericulata/South Korea/K17-1638-5/2017(H6N5)	EPI1526586 99.16%	99.16%
MP	MN749565	A/mallard/Kentucky/AH00729344/2016(H7N2)	EPI952771 99.59%	97.66%
NS	MN749566	A/blue-wingedteal/Missouri/17OS3211/2017(A/H3N1)	EPI1537413 99.52%	99.40%

## Supplementary Materials

Supplementary materials can be found at https://www.mdpi.com/1999-4915/12/7/774/s1. Figure S1, host information by COI gene sequence (A) and information of bird species identification (B). Figure S2, phylogenetic tree of whole genome of the K6 avian influenza virus (AIV) compared to influenza sequences available in NCBI and GISAID. Figure S3, maximum likelihood phylogenetic trees showing inferred relationships among nucleotide sequences for the complete coding regions of K6 and K17 gene segments. Figure S4, raw ELISA data to conduct TCID50 assay. Figure S5, raw ELISA data to conduct TCID50 assay to measure virus K6-H6N5, H1N1, H7N1 titer in lung at day 3 (A); day 6 (B); day 14 (C) post infected. Figure S6, lungs from (A) uninfected control; (B) K6-H6N5; (C) H7N1; (D) H7N1 -infected mouse at 6 dpi. Scale bar: 0.5cm. Figure S7, location map of mandarin duck marked with satellite transmitters in Korea in October in 2017. Figure S8, spatial distribution patterns of Bean geese during Jan 2017- Nov 2017. Table S1, information of strains with more than 99% genetic homology.
