# Supplementary material for: Genetic Characterization of a Novel North American-Origin Avian Influenza A (H6N5) Virus Isolated from Bean Goose of South Korea in 2018
Source: Viruses. 2020 Jul 17;12(7):774. doi: 10.3390/v12070774 (PMC7411716; doi:10.3390/v12070774)
Supplement: Supplementary file 1 [file viruses-12-00774-s001.pdf]

Supplementary data

Genetic characterization of a novel North America-Origin avian Influenza A (H6N5) virus isolated from Bean gooses in South Korea in 2018

Ngoc Minh Nguyen[1], Haan Woo Sung[2], Ki-Jung Yun[3], Hyun Park[1§], and Seon-Ju Yeo[1§]

Figure S1. Host information by COI gene sequence. Initial input of Barcoding COI gene (A) and information of bird species identification (B)

Table S1. Information of strains with more than 99% genetic homology

Figure S2. Phylogenetic tree of whole genome of the K6 avian influenza virus (AIV) compared to influenza sequences available in NCBI and GISAID.

Figure S3. Maximum likelihood phylogenetic trees showing inferred relationships among nucleotide sequences for the complete coding regions of K6 and K17 gene segments.

Figure S4. Raw ELISA data to conduct $TCID_{50}$ assay.

Figure S5. Raw ELISA data to conduct $TCID_{50}$ assay to measure virus K6-H6N5, H1N1, H7N1 titer in lung at day 3 (A); day 6 (B); day 14 (C) post infected.

Figure S6. Lungs from (A) uninfected control; (B) K6-H6N5; (C) H7N1; (D) H7N1 - infected mouse at 6 dpi.

Figure S7. Location map of mandarin duck marked with satellite transmitters in Korea in October in 2017.

Figure S8. Spatial distribution patterns of Bean geese during Jan 2017- Nov 2017.

(A)

CTCTAGGCGACGACCAAATTTACATGTAATCGTTACCGCTCACGCCTTTGTAATA
ATCTTCTTTATAGTCATACCCATCATGATCGGAGGATTCGGCAACTGATTAGTCC
CCCTCATAATCGGTGCCCCCGACATAGCATTCCCGCGAATAAACAACATAAGCT
TTTGACTCCTCCCCCCATCATTCCTCCTGCTACTAGCTTCATCCACTGTAGAAGCT
GGCGCCGGCACAGGCTGAACTGTCTACCCTCCCCTAGCAGGTAACCTCGCCCAC
GCCGGAGCTTCAGTAGACCTGGCTATCTTCTCACTCCACTTAGCCGGTATCTCCT
CCATTCTTGGAGCCATCAACTTTATCACCACAGCTATCAACATAAAACCCCCCG
CACTCTCACAATACCAAACCCCACTATTTGTCTGATCCGTGCTAATTACCGCCAT
CCTACTCCTTCTATCACTCCCCGTACTCGCCGCCGGTATTACAATACTACTAACT
GATCGAAACCTAAACACCACATTCTTCGATCCCGCTGGAGGAGGAGACCCAAT
CCTGTACCAACACCTATTCTGATTTTTC

(B)

BOLD TaxonID Taxonomy Report

Order	Family	Species
Anseriformes[99 individuals][11 species]		
	Anatidae[99 individuals][11 species]	
		Anser albifrons[23 individuals]
		Anser anser[19 individuals]
		Anser brachyrhynchus[5 individuals]
		Anser caerulescens[11 individuals]
		Anser cygnoides[10 individuals]
		Anser erythropus[4 individuals]
		Anser fabalis[11 individuals]
		Anser indicus[6 individuals]
		Anser rossii[2 individuals]
		Anser sp.[1 individuals]
		Branta canadensis[7 individuals]

Figure S1. Host information by COI gene sequence. Initial input of Barcoding COI gene (A) and information of bird species identification (B)

Table S1. Information of strains with more than 99% genetic homology

Gene	K6		K17	
	Strain	% genetic homology	Strain	% genetic homology
PB2	A/Mallard/Alaska/AH0029066S.1.A/2016(H12N5) (MN254630.1)	99.34	A/Mallard/Alaska/AH0029066S.1.A/2016 (H12N5) (MN254630.1)	99.62
	A/Aix galericulata/South Korea/K17-1638-5/2017(H6N5) (MK830104.1)	99.21	A/Mallard/California/D1713638/2017(H12N5) (MK995808.1)	99.15
	A/glaucous-winged gull/Southcentral Alaska/16MB03648/2016(H5N2) (CY239407.1)	99.17	A/Mallard/Utah/D1801802/2018(H5N2) (MK995816.1)	99.1
			A/glaucous-winged gull/Southcentral Alaska/16MB03648/2016(H5N2) (CY239407.1)	99.44
PB1	A/Mallard/Alaska/AH0029066S.2.A/2016 (H12N5) (MN254483.1)	99.56	A/American black duck/Maryland/16OS2661/2017(H7N3) (MK236794.1)	99.27
	A/glaucous-winged gull/Southcentral Alaska/16MB03648/2016(H5N2) (CY239406.1)	99.38		
	A/northern pintail/Alaska/16-041335-6/2016(H5N2) (MH546889.1)	99.21		
	A/northern pintail/Alaska/16-041335-18/2016(H5N2) (MH546873.1)	99.21		
	A/northern pintail/Alaska/16-041335-16/2016(H5N2) (MH546857.1)	99.16		
	A/mallard duck/California/UCD738/2016(H4N5) (KY990805.1)	99.03		
PA	A/Aix galericulata/South Korea/K17-1638-5/2017(H6N5) (MK830102.1)	99.49	A/Mallard/California/D1713634/2017(H9N2) (MK995798.1)	99.33
	A/emperor goose/Alaska/UGAI15-6737/2015(H3N8) (KX949459.1)	99.26	A/Northern Shoveler/California/D1715961/2017(H8N4) (MK995838.1)	99.28
	A/emperor goose/Alaska/UGAI15-6758/2015(H3N8) (KX949467.1)	99.26	A/Green-Winged Teal/California/D1800952/2018(H9N2) (MK995782.1)	99.28
	A/emperor goose/Alaska/UGAI15-6759/2015(H3N8) (KX949475.1)	99.26	A/Green-Winged Teal/California/D1800177/2018(mixed) (MK995772.1)	99.28
	A/emperor goose/Alaska/UGAI15-6734/2015(H3N8) (KY131296.1)	99.21	A/mallard/Alberta/71/2017(H3N8) (MH411975.1)	99.28
	A/Mallard/California/D1713634/2017(H9N2) (MK995798.1)	99.16	A/mallard/Alberta/40/2017(H3N8) (MH411894.1)	99.33
	A/mallard/Alberta/71/2017(H3N8) (MH411975.1)	99.16	A/mallard/Alberta/362/2017(H3N8) (MH637046.1)	99.24
	A/mallard/Alberta/40/2017(H3N8) (MH411894.1)	99.16	A/mallard/Alberta/81/2017(H3N8) (MH412146.1)	99.28

	A/mallard/Southcentral Alaska/12ML01469/2014(mixed) (CY194170.1)	99.16	A/lesser snow goose/Oregon/16-006224-1/2016(H5N3) (MH546530.1)	99.19
	A/Northern Shoveler/California/D1715961/2017 (H8N4) (MK995838.1)	99.12	A/blue-winged teal/Alberta/98/2017(H3N8) (MH412308.1)	99.28
	A/Green-Winged Teal/California/D1800952/2018(H9N2) (MK995782.1)	99.12	A/northern shoveler/California/AH0076719/2016(H7N3) (KY550793.1)	99.1
	A/Green-Winged Teal/California/D1800177/2018(mixed) (MK995772.1)	99.12	A/mallard/Alberta/182/2017(H3N8) (MH637074.1)	99.15
	A/mallard/Alberta/273/2017(H4N6) (MH981933.1)	99.12	A/Green-Winged Teal/Nevada/D1612806/2016(H5N2) (MK928161.1)	99.06
	A/mallard/Alberta/498/2017(H4N6) (MH932484.1)	99.12	A/blue-winged teal/Alberta/205/2017(H4N6) (MH932335.1)	99.28
	A/blue-winged teal/Alberta/210/2017(H4N6) (MH932445.1)	99.12	A/mallard/Alberta/249/2017(H4N6) (MH932343.1)	99.28
	A/blue-winged teal/Alberta/209/2017(H4N6) (MH932407.1)	99.12	A/mallard/Alberta/243/2017(H4N6) (MH932273.1)	99.28
	A/mallard/Alberta/231/2017(H4N6) (MH932391.1)	99.12	A/mallard/Alberta/245/2017(H4N6) (MH932128.1)	99.28
	A/mallard/Alberta/228/2017(H4N6) (MH932383.1)	99.12	A/American green-winged teal/Illinois/17OS2545/2017(H4N6) (MK237839.1)	99.15
	A/blue-winged teal/Alberta/207/2017(H4N6) (MH932311.1)	99.12	A/mallard/Alberta/251/2017(H4N6) (MH932224.1)	99.28
	A/mallard/Alberta/129/2017(mixed) (MH932359.1)	99.12	A/blue-winged teal/Illinois/17OS2553/2017(H6N1) (MK237200.1)	99.01
	A/blue-winged teal/Alberta/205/2017(H4N6) (MH932335.1)	99.12	A/mallard/Southcentral Alaska/12ML01469/2014(mixed) (CY194170.1)	99.32
	A/blue-winged teal/Alberta/211/2017(H4N6) (MH932303.1)	99.12	A/bufflehead/Alaska/512/2014(H4N8) (KT338435.1)	99.23
	A/mallard/Alberta/249/2017(H4N6) (MH932343.1)	99.12	A/mallard/Washington/16-027073-5/2016(H5N2) (MH546802.1)	99.28
	A/mallard/Alberta/253/2017(H4N6) (MH932287.1)	99.12	A/mallard/Washington/16-027073-48/2016(H5N2) (MH546794.1)	99.28
	A/mallard/Alberta/243/2017(H4N6) (MH932273.1)	99.12	A/mallard/Washington/16-027073-46/2016(H5N2) (MH546786.1)	99.28
	A/mallard/Alberta/241/2017(H4N6) (MH932257.1)	99.12	A/mallard/Washington/16-027073-22/2016(H5N2) (MH546770.1)	99.28
	A/blue-winged teal/Alberta/206/2017(H4N6) (MH932265.1)	99.12	A/mallard/Washington/16-027073-21/2016(H5N2) (MH546762.1)	99.28

	A/mallard/Alberta/255/2017(H4N6) (MH932241.1)	99.12	A/mallard/Alberta/498/2017(H4N6) (MH932484.1)	99.28
	A/mallard/Alberta/234/2017(H4N6) (MH932249.1)	99.12	A/mallard/Alberta/231/2017(H4N6) (MH932391.1)	99.28
	A/mallard/Alberta/251/2017(H4N6) (MH932224.1)	99.12	A/emperor goose/Alaska/UGAI15-6737/2015(H3N8) (KX949459.1)	99.41
	A/mallard/Alberta/233/2017(H4N6) (MH932184.1)	99.12	A/emperor goose/Alaska/UGAI15-6758/2015(H3N8) (KX949467.1)	99.41
	A/mallard/Alberta/123/2017(H4N6) (MH932144.1)	99.12	A/emperor goose/Alaska/UGAI15-6759/2015(H3N8) (KX949475.1)	99.41
	A/mallard/Alberta/245/2017(H4N6) (MH932128.1)	99.12	A/blue-winged teal/Alberta/210/2017(H4N6) (MH932445.1)	99.27
	A/mallard/Alberta/232/2017(H4N6) (MH932112.1)	99.12	A/blue-winged teal/Alberta/209/2017(H4N6) (MH932407.1)	99.27
	A/mallard/Alberta/201/2017(H4N6) (MH932120.1)	99.12	A/mallard/Alberta/228/2017(H4N6) (MH932383.1)	99.27
	A/mallard/Alberta/362/2017(H3N8) (MH637046.1)	99.12	A/blue-winged teal/Alberta/207/2017(H4N6) (MH932311.1)	99.27
	A/blue-winged teal/Alberta/98/2017(H3N8) (MH412308.1)	99.12	A/mallard/Alberta/129/2017(mixed) (MH932359.1)	99.27
	A/mallard/Alberta/81/2017(H3N8) (MH412146.1)	99.12	A/mallard/Alberta/253/2017(H4N6) (MH932287.1)	99.27
	A/mallard/Washington/16-027073-5/2016(H5N2) (MH546802.1)	99.12	A/mallard/Alberta/241/2017(H4N6) (MH932257.1)	99.27
	A/mallard/Washington/16-027073-48/2016(H5N2) (MH546794.1)	99.12	A/blue-winged teal/Alberta/206/2017(H4N6) (MH932265.1)	99.27
	A/mallard/Washington/16-027073-46/2016(H5N2) (MH546786.1)	99.12	A/mallard/Alberta/123/2017(H4N6) (MH932144.1)	99.27
	A/mallard/Washington/16-027073-22/2016(H5N2) (MH546770.1)	99.12	A/mallard/Alberta/232/2017(H4N6) (MH932112.1)	99.27
	A/mallard/Washington/16-027073-21/2016(H5N2) (MH546762.1)	99.12	A/mallard/Alberta/273/2017(H4N6) (MH981933.1)	99.27
	A/mallard/California/16-038989-4/2016(H5N2) (MH546570.1)	99.12	A/blue-winged teal/Alberta/211/2017(H4N6) (MH932303.1)	99.27
	A/mallard/Southcentral Alaska/12ML00991_AAF1/2014(H5N2) (CY194194.1)	99.12	A/mallard/Alberta/255/2017(H4N6) (MH932241.1)	99.27
	A/bufflehead/Alaska/512/2014(H4N8) (KT338435.1)	99.07	A/mallard/Alberta/201/2017(H4N6) (MH932120.1)	99.27
	A/mallard/Washington/16-028147-4/2016(H5N2) (MH546810.1)	99.07	A/mallard/Washington/16-028147-4/2016(H5N2) (MH546810.1)	99.23
	A/mallard/Washington/16-027073-45/2016(H5N2) (MH546778.1)	99.07	A/emperor goose/Alaska/UGAI15-6734/2015(H3N8) (KY131296.1)	99.36

	A/lesser snow goose/Oregon/16-006224-1/2016(H5N3) (MH546530.1)	99.07	A/mallard/Southcentral Alaska/12ML00991_AAF1/2014(H5N2) (CY194194.1)	99.27
	A/mallard/Alberta/53/2017(H3N8) (MH932351.1)	99.02	A/mallard/Alberta/233/2017(H4N6) (MH932184.1)	99.27
	A/mallard/Alberta/30/2017(H3N8) (MH932327.1)	99.02	A/mallard/Alberta/234/2017(H4N6) (MH932249.1)	99.27
	A/mallard/Alberta/230/2017(H3N8) (MH932295.1)	99.02	A/mallard/Washington/16-027073-45/2016(H5N2) (MH546778.1)	99.23
	A/mallard/Alberta/246/2017(H3N8) (MH932176.1)	99.02	A/mallard/California/16-038989-4/2016(H5N2) (MH546570.1)	99.18
	A/mallard/Alberta/292/2017(H3N8) (MH932160.1)	99.02	A/mallard/Nevada/16-035274-2/2016(H5N2) (MH546674.1)	99.14
	A/blue-winged teal/Alberta/4/2017(H3N8) (MH932152.1)	99.02	A/mallard/Oregon/16-028808-2/2016(H5N2) (MH546714.1)	99.09
	A/mallard/Alberta/270/2017(mixed) (MH932136.1)	99.02	A/mallard/Southcentral Alaska/12ML01175/2014(mixed) (CY206803.1)	99.14
	A/blue-winged teal/Alberta/3/2017(H3N8) (MH411905.1)	99.02	A/northern pintail/Alaska/UGAI15-6407/2015(H3N8) (KX949451.1)	99.14
			A/green-winged teal/Alaska/UGAI15-7287/2015(H3N8) (KX949491.1)	99.14
			A/northern pintail/Alaska/UGAI15-7291/2015(H3N8) (KX949507.1)	99.14
			A/northern pintail/Alaska/UGAI15-7300/2015(H6N1) (KY131359.1)	99.14
			A/green-winged teal/Alaska/UGAI15-6067/2015(H8N4) (KY131249.1)	99.14
			A/American green-winged teal/Nevada/16-033700-1/2016(H5N2) (MH546202.1)	99.05
			A/northern pintail/Alaska/UGAI15-7288/2015(H3N8) (KX949499.1)	99.09
			A/green-winged teal/Alaska/UGAI15-7286/2015(H3N8) (KX949483.1)	99.09
			A/mallard/Alberta/53/2017(H3N8) (MH932351.1)	99.18
			A/mallard/Alberta/30/2017(H3N8) (MH932327.1)	99.18
			A/mallard/Alberta/230/2017(H3N8) (MH932295.1)	99.18
			A/mallard/Alberta/246/2017(H3N8) (MH932176.1)	99.18
			A/mallard/Alberta/292/2017(H3N8) (MH932160.1)	99.18
			A/blue-winged teal/Alberta/4/2017(H3N8) (MH932152.1)	99.18
			A/mallard/Alberta/270/2017(mixed) (MH932136.1)	99.18

			A/blue-winged teal/Alberta/3/2017(H3N8) (MH411905.1)	99.18
			A/northern pintail/Alaska/UGAI15-7399/2015(H3N6) (KY131391.1)	99.04
			A/northern pintail/Alaska/UGAI15-6994/2015(H6N1) (KY131328.1)	99.04
			A/mallard/Alaska/UGAI15-7301/2015(mixed) (KX949547.1)	99
HA	A/Aix galericulata/South Korea/K17-1638-5/2017(H6N5) (MK830100.1)	99.3		
NP	A/lesser scaup/Wisconsin/17OS5811/2017(H6N1) (MK236919.1)	99.67	A/American green-winged teal/Missouri/17OS3212/2017(H3N8) (MK236738.1)	99.55
	A/Northern Pintail/Arizona/D1800538/2018(H12N2) (MK995828.1)	99.6	A/blue-winged teal/Illinois/17OS2636/2017(H4N6) (MK237055.1)	99.42
	A/American wigeon/California/LDC523/2016(H6N1) (MH251193.1)	99.6	A/mallard/Ohio/16OS0620/2016(H10N7) (KY561279.1)	99.23
	A/northern shoveler/Missouri/17OS4821/2017(H6N1) (MK237918.1)	99.6	A/Blue-winged Teal/North Carolina/AH0114567S.5.A/2017(H4N6) (MN254118.1)	99.11
	A/American wigeon/Missouri/17OS4780/2017(H6N1) (MK237159.1)	99.53	A/Northern Shoveler/Nevada/D1615770/2016(H10N9) (MK928247.1)	99.04
	A/American green-winged teal/Tennessee/18OS0447/2018(H5N2) (MK237929.1)	99.47	A/mallard/Idaho/16-034834-1/2016(H5N2) (MH546580.1)	99.1
	A/Gadwall/Ohio/18OS2668/2018(H6N1) (MN431215.1)	99.06	A/mallard duck/Illinois/17OS1431/2017(H10N7) (MG280472.1)	99.04
	A/American Wigeon/Ohio/18OS1967/2018(H6N1) (MN431212.1)	99	A/green-winged teal/Missouri/15OS6706/2015(H6N1) (KY563727.1)	99.03
	A/American Green-winged Teal/Ohio/18OS2656/2018(H6N1) (MN430901.1)	99	A/mallard/Idaho/UGAI16-1929/2016(H3N8) (CY235197.1)	99.03
	A/mallard/Arkansas/AI09-5761/2009 (H2N3) (CY141020.1)	99		
NA	A/Aix galericulata/South Korea/K17-1638-5/2017(H6N5) (MK830105.1)	99.16		
MP	A/mallard/Kentucky/AH00729344/2016 (H7N2) (KY550970.1)	99.59	A/Northern Pintail/Arizona/D1800538/2018(H12N2) (MK995826.1)	99.22
	A/mallard duck/Ohio/15OS7510/2015(H11N3) (KY561112.1)	99.49	A/Blue-winged Teal/South Dakota/AH0037508S.7.A/2015(N8) (MN253741.1)	99.12
	A/Northern shoveler/Mississippi/16OS5984/2016 (H6N1) (MG280067.1)	99.39	A/Mexican duck/EstadodeMexico; Lerma/M_UIFMVZ377/2016(H5N2) (MK828141.1)	99.03

	A/Mallard Duck/Ohio/16OS0899/2016(H2N3) (MN552532.1)	99.29	A/mallard/Ohio/17OS5967/2017(H3N1) (MK236986.1)	99
	A/mallard/Idaho/UGAI16-1941/2016(mixed) (CY235246.1)	99.29	A/mallard/Pennsylvania/UGAI15-4892/2015(H1N1) (MH341857.1)	99
	A/blue-winged teal/Louisiana/UGAI15-2510/2015(H7N3) (KY013813.1)	99.29	A/mallard duck/Ohio/16OS0859/2016(H3N8) (MG280174.1)	99
	A/blue-winged teal/Texas/UGAI14-3653/2014(mixed) (CY205500.1)	99.29	A/mallard duck/Ohio/16OS0862/2016(H3N8) (MG279891.1)	99
	A/northern pintail/Alaska/819/2014(H4N6) (KT338548.1)	99.29	A/mallard duck/Ohio/16OS0846/2016(H3N8) (MG280490.1)	99
	A/American green-winged teal/Alaska/306/2014(H3N3) (KT338352.1)	99.29	A/mallard duck/Ohio/16OS0843/2016(H3N8) (MG280070.1)	99
	A/Mallard/Pennsylvania/AH0119501S.7.A/2017(Mixed) (MN253842.1)	99.19	A/American wigeon/Maryland/16OS5139/2016(H6N8) (MG280072.1)	99
	A/Mallard/Illinois/17OS2751/2017(H5N2) (MN431105.1)	99.19	A/blue-winged teal/Texas/UGAI15-6811/2015(H4N8) (KY014007.1)	99
	A/mallard/Washington/16-027073-5/2016(H5N2) (MH546806.1)	99.19	A/blue-winged teal/Texas/UGAI15-6848/2015(H4N8) (KY014015.1)	99
	A/mallard/Washington/16-027073-48/2016(H5N2) (MH546798.1)	99.19	A/mallard/Utah/AH0020690/2015(H7N3) (KY551350.1)	99
	A/mallard/Washington/16-027073-46/2016(H5N2) (MH546790.1)	99.19	A/mallard/Idaho/AH0011522/2015(H7N7) (KY551319.1)	99
	A/mallard/Washington/16-027073-45/2016(H5N2) (MH546782.1)	99.19		
	A/mallard/Washington/16-027073-22/2016(H5N2) (MH546774.1)	99.19		
	A/mallard/Washington/16-027073-21/2016(H5N2) (MH546766.1)	99.19		
	A/Northern shoveler/Ohio/16OS5078/2016(H10N7) (MG280008.1)	99.19		
	A/mallard/Idaho/UGAI16-1957/2016(mixed) (CY235299.1)	99.19		
	A/cinnamon teal/Idaho/UGAI16-1910/2016(mixed) (CY235050.1)	99.19		
	A/American wigeon/California/HS001/2015(H6N2) (KY983210.1)	99.19		
	A/mallard/Louisiana/UGAI15-0711/2015(H7N3) (KY013743.1)	99.19		
	A/blue-winged teal/Texas/UGAI15-2283/2015(H7N3) (KY013783.1)	99.19		
	A/blue-winged teal/Texas/UGAI15-2338/2015(H7N3) (KY013791.1)	99.19		

	A/northern pintail/Alaska/861/2014(H3N8) (KT338564.1)	99.19		
	A/northern pintail/Alaska/580/2014(H3N8) (KT338486.1)	99.19		
	A/mallard/Alaska/468/2014(H4N6) (KT338399.1)	99.19		
	A/harlequin duck/Alaska/46/2014(H1N1) (KP896494.1)	99.19		
	A/American green-winged teal/Alaska/44493-588/2009(H3N8) (JX081197.1)	99.19		
	A/Mallard/Ohio/18OS1280/2018(H7N 1) (MN431087.1)	99.08		
	A/mallard/Alberta/572/2017(mixed) (MH981948.1)	99.08		
	A/cinnamon teal/Idaho/UGAI16-1901/2016(H4N6) (CY235163.1)	99.08		
	A/cinnamon teal/Idaho/UGAI16-1881/2016(H4N6) (CY235123.1)	99.08		
	A/mallard/Alaska/UGAI15-6397/2015(H6N8) (KX949431.1)	99.08		
	A/green-winged teal/Alaska/UGAI15-6400/2015(H3N8) (KX949439.1)	99.08		
	A/northern pintail/Alaska/UGAI15-6407/2015(H3N8) (KX949455.1)	99.08		
	A/northern pintail/Alaska/UGAI15-6978/2015(H6N1) (KY131308.1)	99.08		
	A/green-winged teal/Alaska/239/2013(H3N8) (KY130980.1)	99.08		
	A/mallard/Southcentral Alaska/12ML01055/2014(H1N1) (CY206790.1)	99.08		
	A/northern pintail/Alaska/562/2014(H3N8) (KT338463.1)	99.08		
	A/northern pintail/Interior Alaska/8BM3696/2008(H3N8) (CY080093.1)	99.08		
NS	A/blue-winged teal/Missouri/17OS3211/2017(H3N1) (MK236976.1)	99.52	A/northern pintail/Alaska/16-041335-6/2016(H5N2) (MH546895.1)	99.44
	A/glaucous-winged gull/Southcentral Alaska/16MB03648/2016(H5N2) (CY239404.1)	99.52	A/northern pintail/Alaska/16-041335-18/2016(H5N2) (MH546879.1)	99.44
	A/Aix galericulata/South Korea/K17-1638-5/2017(H6N5) (MK830107.1)	99.4	A/northern pintail/Alaska/16-041335-16/2016(H5N2) (MH546863.1)	99.44

	A/northern pintail/Alaska/16-041335-6/2016(H5N2) (MH546895.1)	99.4	A/Mallard/Alaska/AH0029066S.8.A/2016 (H12N5) (MN254195.1)	99.33
	A/northern pintail/Alaska/16-041335-18/2016(H5N2) (MH546879.1)	99.4	A/mallard/Alberta/412/2017(H11N9) (MH637323.1)	99.33
	A/northern pintail/Alaska/16-041335-16/2016(H5N2) (MH546863.1)	99.4	A/mallard/Alberta/414/2017(H11N9) (MH637219.1)	99.44
	A/Mallard/Alaska/AH0029066S.8.A/2016(H12N5) (MN254195.1)	99.28	A/mallard/Alberta/380/2017(H11N9) (MH412478.1)	99.33
	A/mallard/Alberta/305/2018(H4N6) (MK861133.1)	99.28	A/mallard/Alberta/350/2017(H11N9) (MH411885.1)	99.33
	A/mallard/Alberta/299/2018(H4N6) (MK861132.1)	99.28	A/mallard/Alberta/416/2017(H11N9) (MH637129.1)	99.44
	A/mallard/Alberta/311/2018(H4N6) (MK861141.1)	99.28	A/Northern Pintail/Washington/AH0042510S.8.A/2015(H9N2) (MN254234.1)	99.21
	A/mallard/Alberta/278/2018(H4N6) (MK861123.1)	99.28	A/mallard/Alberta/328/2017(H3N8) (MH637140.1)	99.21
	A/mallard/Alberta/586/2017(H3N8) (MH932525.1)	99.28	A/blue-winged teal/Iowa/13OS2349/2013(H4N8) (KJ568157.1)	99.21
	A/mallard/Alberta/412/2017(H11N9) (MH637323.1)	99.28	A/blue-winged teal/Alberta/164/2017(H3N8) (MH412138.1)	99.21
	A/mallard/Alberta/411/2017(H3N8) (MH637258.1)	99.28	A/blue-winged teal/Alberta/162/2017(H3N8) (MH411911.1)	99.21
	A/mallard/Alberta/414/2017(H11N9) (MH637219.1)	99.28	A/mallard/Alberta/411/2017(H3N8) (MH637258.1)	99.32
	A/mallard/Alberta/328/2017(H3N8) (MH637140.1)	99.28	A/Mallard/California/D1713634/2017(H9N2) (MK995797.1)	99.1
	A/mallard/Alberta/416/2017(H11N9) (MH637129.1)	99.28	A/mallard/California/UCD1357/2017(H3N8) (MH251182.1)	99.1
	A/mallard/Alberta/380/2017(H11N9) (MH412478.1)	99.28	A/blue-winged teal/Guatemala/CIP049-S_H120-33/2014(H12N4) (MK326849.1)	99.1
	A/blue-winged teal/Alberta/164/2017(H3N8) (MH412138.1)	99.28	A/blue-winged teal/Guatemala/CIP049-H116-51/2013(H5N3) (MK326794.1)	99.1
	A/blue-winged teal/Alberta/162/2017(H3N8) (MH411911.1)	99.28	A/blue-winged teal/Guatemala/CIP049-H116-50/2013(H5N3) (MK326786.1)	99.1
	A/mallard/Alberta/350/2017(H11N9) (MH411885.1)	99.28	A/blue-winged teal/Guatemala/CIP049-H116-22/2013(H5N3) (MK326766.1)	99.1
	A/Northern Pintail/Washington/AH0042510S.8.A/2015(H9N2) (MN254234.1)	99.16	A/blue-winged teal/Guatemala/CIP049-H116-10/2013(H5N3) (MK326745.1)	99.1
	A/Northern Shoveler/18OS3591/2018(H10N3) (MN430734.1)	99.16	A/blue-winged teal/Guatemala/CIP049-H121-36/2014(H2N9) (MK326709.1)	99.1
	A/Green-Winged Teal/Southcentral Alaska/18MB01702/2018(H10N7) (MN210264.1)	99.16	A/blue-winged teal/Guatemala/CIP049-H119-01/2014(H12N3) (MK326693.1)	99.1

	A/mallard/Alberta/595/2017(H3N8) (MH932475.1)	99.16	A/blue-winged teal/Guatemala/CIP049H105-08/2011(H3N3) (KY673160.1)	99.1
	A/mallard/Alberta/587/2017(H3N8) (MH932468.1)	99.16	A/blue-winged teal/Guatemala/CIP049H102-18/2011(H1N3) (KX960456.1)	99.1
	A/mallard/Alberta/408/2017(H3N8) (MH637428.1)	99.16	A/green-winged teal/Ohio/16OS3692/2016(H4N2) (MG280503.1)	99.21
	A/mallard/Alberta/398/2017(H3N8) (MH637216.1)	99.16	A/mallard/Alberta/595/2017(H3N8) (MH932475.1)	99.21
	A/Mallard/Ohio/18OS1880/2018(H6N1) (MN430975.1)	99.05	A/mallard/Alberta/408/2017(H3N8) (MH637428.1)	99.21
	A/Mallard/California/D1713634/2017(H9N2) (MK995797.1)	99.05	A/mallard/Alberta/398/2017(H3N8) (MH637216.1)	99.21
	A/mallard/California/UCD1357/2017(H3N8) (MH251182.1)	99.05	A/glaucous-winged gull/Southcentral Alaska/16MB03648/2016(H5N2) (CY239404.1)	99.65
	A/lesser scaup/Wisconsin/17OS5811/2017(H6N1) (MK237378.1)	99.05		
	A/American wigeon/Missouri/17OS4780/2017(H6N1) (MK237197.1)	99.05		
	A/greater scaup/Wisconsin/17OS4533/2017(H6N1) (MK236725.1)	99.05		
	A/blue-winged teal/Guatemala/CIP049-S_H120-33/2014(H12N4) (MK326849.1)	99.05		
	A/blue-winged teal/Guatemala/CIP049-H116-51/2013(H5N3) (MK326794.1)	99.05		
	A/blue-winged teal/Guatemala/CIP049-H116-50/2013(H5N3) (MK326786.1)	99.05		
	A/blue-winged teal/Guatemala/CIP049-H116-22/2013(H5N3) (MK326766.1)	99.05		
	A/blue-winged teal/Guatemala/CIP049-H116-10/2013(H5N3) (MK326745.1)	99.05		
	A/blue-winged teal/Guatemala/CIP049-H121-36/2014(H2N9) (MK326709.1)	99.05		
	A/blue-winged teal/Guatemala/CIP049-H119-01/2014(H12N3) (MK326693.1)	99.05		
	A/American green-winged teal/Oregon/17-000383-3/2016(H5N2) (MH546215.1)	99.05		

	A/green-winged teal/Ohio/16OS3692/2016(H4N2) (MG280503.1)	99.05		
	A/mallard/Utah/AH0075849/2016(H7N3) (KY551423.1)	99.05		
	A/mallard/Illinois/14OS3635/2014(mixed) (KY463882.1)	99.05		
	A/mallard/Idaho/ND0002646/2013(H4N6) (KM373990.1)	99.05		
	A/blue-winged teal/Iowa/13OS2349/2013(H4N8) (KJ568157.1)	99.05		
	A/mallard/Wisconsin/11OS3577/2011(H6N5) (CY166214.1)	99.05		
	A/mallard/Ohio/11OS2045/2011(mixed) (CY132006.1)	99.05		

A. PB2

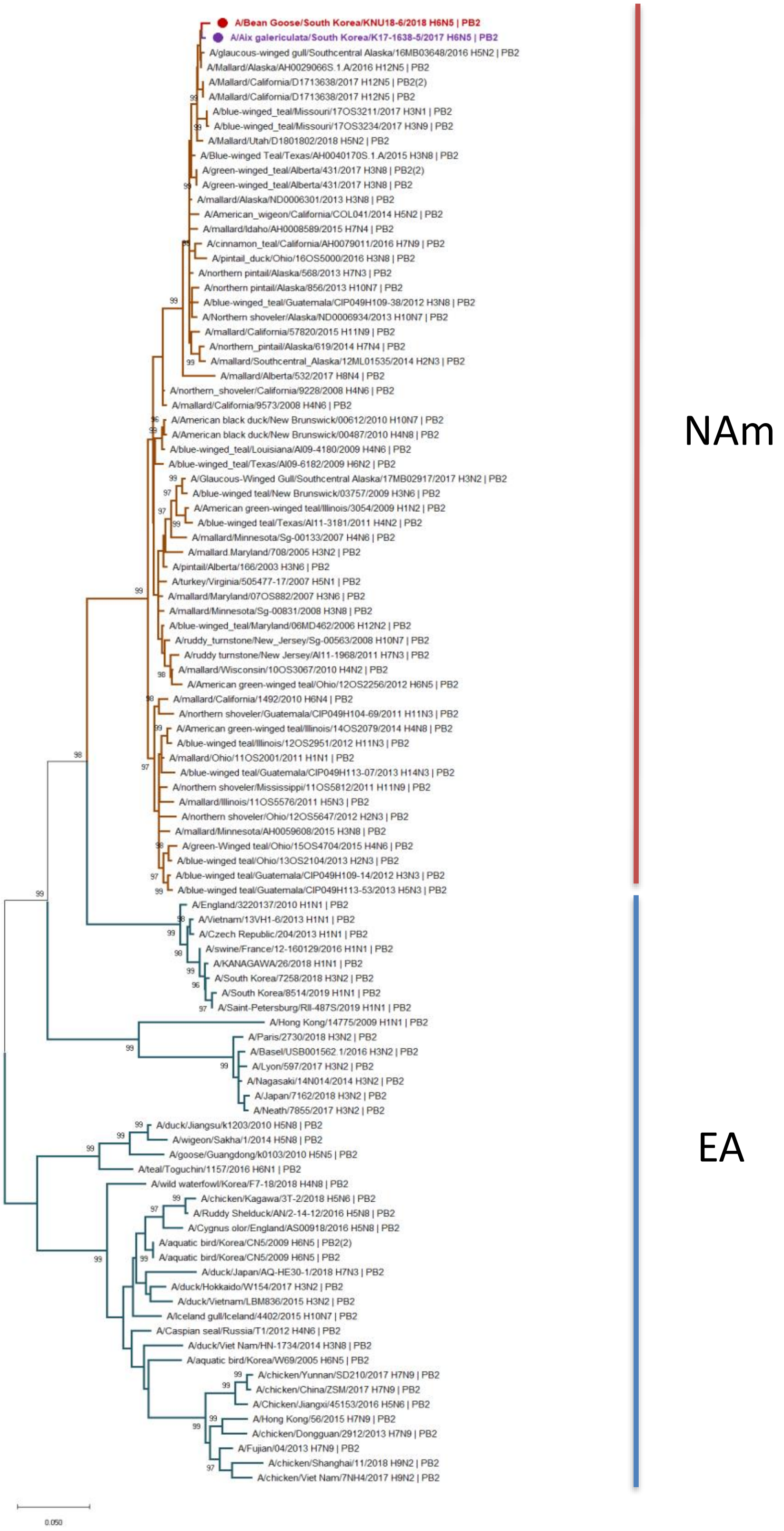
A/Bean Goose/South Korea/KNU18-6/2018 H6N5 | PB2
A/Aix galericulata/South Korea/K17-1638-5/2017 H6N5 | PB2
A/glaucous-winged gull/Southcentral Alaska/16MB03648/2016 H5N2 | PB2
A/Mallard/Alaska/AH0029066S.1.A/2016 H12N5 | PB2
A/Mallard/California/D1713638/2017 H12N5 | PB2(2)
A/Mallard/California/D1713638/2017 H12N5 | PB2
A/blue-winged_teal/Missouri/17OS3211/2017 H3N1 | PB2
A/blue-winged_teal/Missouri/17OS3234/2017 H3N9 | PB2
A/Mallard/Utah/D1801802/2018 H5N2 | PB2
A/Blue-winged Teal/Texas/AH0040170S.1.A/2015 H3N8 | PB2
A/green-winged_teal/Alberta/431/2017 H3N8 | PB2(2)
A/green-winged_teal/Alberta/431/2017 H3N8 | PB2
A/mallard/Alaska/ND0006301/2013 H3N8 | PB2
A/American_wigeon/California/COL041/2014 H5N2 | PB2
A/mallard/Idaho/AH0008589/2015 H7N4 | PB2
A/cinnamon_teal/California/AH0079011/2016 H7N9 | PB2
A/pintail_duck/Ohio/16OS5000/2016 H3N8 | PB2
A/northern pintail/Alaska/568/2013 H7N3 | PB2
A/northern pintail/Alaska/856/2013 H10N7 | PB2
A/blue-winged_teal/Guatemala/CIP049H109-38/2012 H3N8 | PB2
A/Northern shoveler/Alaska/ND0006934/2013 H10N7 | PB2
A/mallard/California/57820/2015 H11N9 | PB2
A/northern_pintail/Alaska/619/2014 H7N4 | PB2
A/mallard/Southcentral_Alaska/12ML01535/2014 H2N3 | PB2
A/mallard/Alberta/532/2017 H8N4 | PB2
A/northern_shoveler/California/9228/2008 H4N6 | PB2
A/mallard/California/9573/2008 H4N6 | PB2
A/American black duck/New Brunswick/00612/2010 H10N7 | PB2
A/American black duck/New Brunswick/00487/2010 H4N8 | PB2
A/blue-winged_teal/Louisiana/AI09-4180/2009 H4N6 | PB2
A/blue-winged_teal/Texas/AI09-6182/2009 H6N2 | PB2
A/Glaucous-Winged Gull/Southcentral Alaska/17MB02917/2017 H3N2 | PB2
A/blue-winged teal/New Brunswick/03757/2009 H3N6 | PB2
A/American green-winged teal/Illinois/3054/2009 H1N2 | PB2
A/blue-winged teal/Texas/AI11-3181/2011 H4N2 | PB2
A/mallard/Minnesota/Sg-00133/2007 H4N6 | PB2
A/mallard.Maryland/708/2005 H3N2 | PB2
A/pintail/Alberta/166/2003 H3N6 | PB2
A/turkey/Virginia/505477-17/2007 H5N1 | PB2
A/mallard/Maryland/07OS882/2007 H3N6 | PB2
A/mallard/Minnesota/Sg-00831/2008 H3N8 | PB2
A/blue-winged_teal/Maryland/06MD462/2006 H12N2 | PB2
A/ruddy_turnstone/New_Jersey/Sg-00563/2008 H10N7 | PB2
A/ruddy turnstone/New Jersey/AI11-1968/2011 H7N3 | PB2
A/mallard/Wisconsin/10OS3067/2010 H4N2 | PB2
A/American green-winged teal/Ohio/12OS2256/2012 H6N5 | PB2
A/mallard/California/1492/2010 H6N4 | PB2
A/northern shoveler/Guatemala/CIP049H104-69/2011 H11N3 | PB2
A/American green-winged teal/Illinois/14OS2079/2014 H4N8 | PB2
A/blue-winged teal/Illinois/12OS2951/2012 H11N3 | PB2
A/mallard/Ohio/11OS2001/2011 H1N1 | PB2
A/blue-winged teal/Guatemala/CIP049H113-07/2013 H14N3 | PB2
A/northern shoveler/Mississippi/11OS5812/2011 H11N9 | PB2
A/mallard/Illinois/11OS5576/2011 H5N3 | PB2
A/northern shoveler/Ohio/12OS5647/2012 H2N3 | PB2
A/mallard/Minnesota/AH0059608/2015 H3N8 | PB2
A/green-Winged teal/Ohio/15OS4704/2015 H4N6 | PB2
A/blue-winged teal/Ohio/13OS2104/2013 H2N3 | PB2
A/blue-winged teal/Guatemala/CIP049H109-14/2012 H3N3 | PB2
A/blue-winged teal/Guatemala/CIP049H113-53/2013 H5N3 | PB2
A/England/3220137/2010 H1N1 | PB2
A/Vietnam/13VH1-6/2013 H1N1 | PB2
A/Czech Republic/204/2013 H1N1 | PB2
A/swine/France/12-160129/2016 H1N1 | PB2
A/KANAGAWA/26/2018 H1N1 | PB2
A/South Korea/7258/2018 H3N2 | PB2
A/South Korea/8514/2019 H1N1 | PB2
A/Saint-Petersburg/RII-487S/2019 H1N1 | PB2
A/Hong Kong/14775/2009 H1N1 | PB2
A/Paris/2730/2018 H3N2 | PB2
A/Basel/USB001562.1/2016 H3N2 | PB2
A/Lyon/597/2017 H3N2 | PB2
A/Nagasaki/14N014/2014 H3N2 | PB2
A/Japan/7162/2018 H3N2 | PB2
A/Neath/7855/2017 H3N2 | PB2
A/duck/Jiangsu/k1203/2010 H5N8 | PB2
A/wigeon/Sakha/1/2014 H5N8 | PB2
A/goose/Guangdong/k0103/2010 H5N5 | PB2
A/teal/Toguchin/1157/2016 H6N1 | PB2
A/wild waterfowl/Korea/F7-18/2018 H4N8 | PB2
A/chicken/Kagawa/3T-2/2018 H5N6 | PB2
A/Ruddy Shelduck/AN/2-14-12/2016 H5N8 | PB2
A/Cygnus olor/England/AS00918/2016 H5N8 | PB2
A/aquatic bird/Korea/CN5/2009 H6N5 | PB2(2)
A/aquatic bird/Korea/CN5/2009 H6N5 | PB2
A/duck/Japan/AQ-HE30-1/2018 H7N3 | PB2
A/duck/Hokkaido/W154/2017 H3N2 | PB2
A/duck/Vietnam/LBM836/2015 H3N2 | PB2
A/Iceland gull/Iceland/4402/2015 H10N7 | PB2
A/Caspian seal/Russia/T1/2012 H4N6 | PB2
A/duck/Viet Nam/HN-1734/2014 H3N8 | PB2
A/aquatic bird/Korea/W69/2005 H6N5 | PB2
A/chicken/Yunnan/SD210/2017 H7N9 | PB2
A/chicken/China/ZSM/2017 H7N9 | PB2
A/Chicken/Jiangxi/45153/2016 H5N6 | PB2
A/Hong Kong/56/2015 H7N9 | PB2
A/chicken/Dongguan/2912/2013 H7N9 | PB2
A/Fujian/04/2013 H7N9 | PB2
A/chicken/Shanghai/11/2018 H9N2 | PB2
A/chicken/Viet Nam/7NH4/2017 H9N2 | PB2
NAm
EA
0.050

a. PB2

NAm

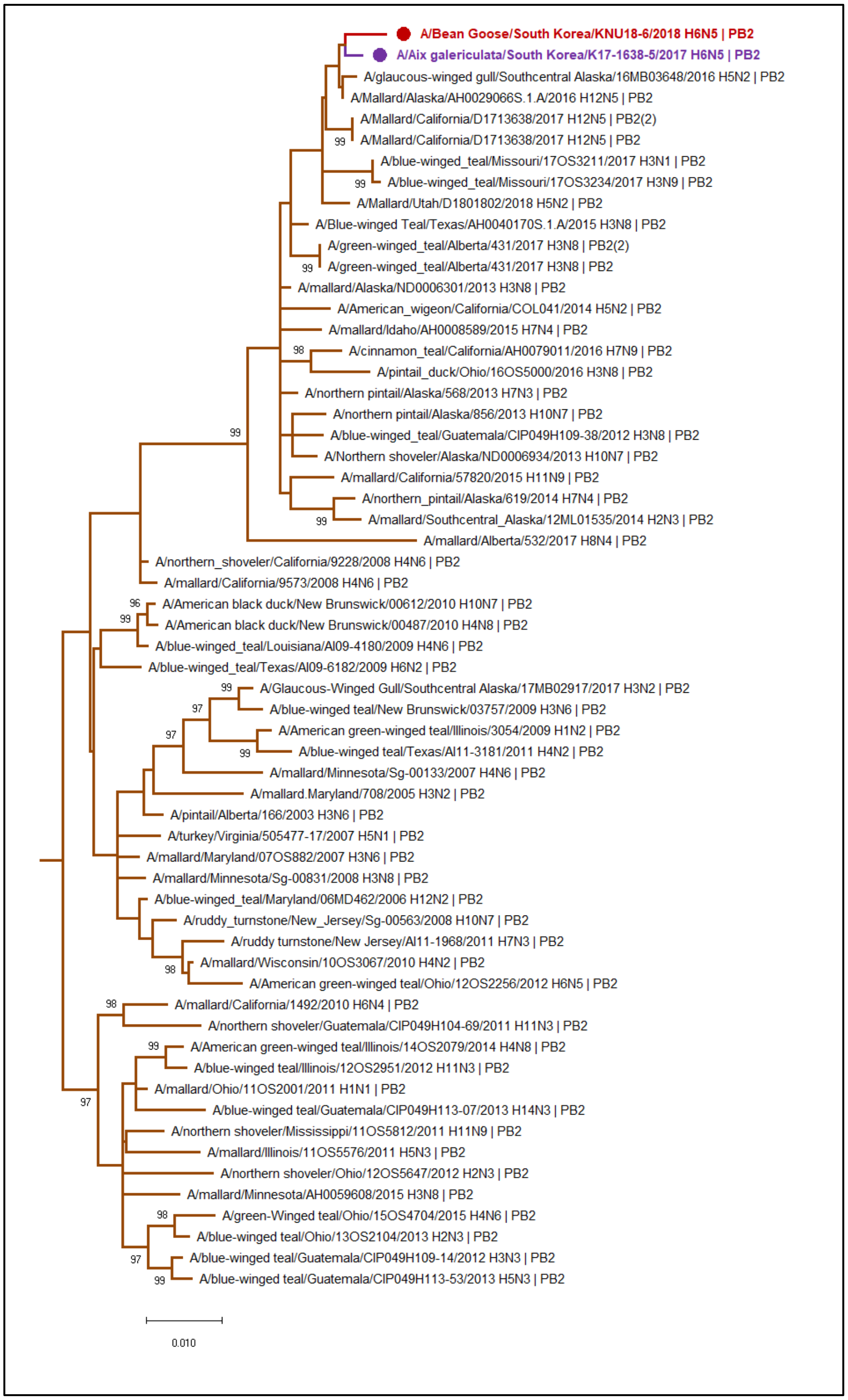

A/Bean Goose/South Korea/KNU18-6/2018 H6N5 | PB2
A/Aix galericulata/South Korea/K17-1638-5/2017 H6N5 | PB2
A/glaucous-winged gull/Southcentral Alaska/16MB03648/2016 H5N2 | PB2
A/Mallard/Alaska/AH0029066S.1.A/2016 H12N5 | PB2
A/Mallard/California/D1713638/2017 H12N5 | PB2(2)
A/Mallard/California/D1713638/2017 H12N5 | PB2
A/blue-winged_teal/Missouri/17OS3211/2017 H3N1 | PB2
A/blue-winged_teal/Missouri/17OS3234/2017 H3N9 | PB2
A/Mallard/Utah/D1801802/2018 H5N2 | PB2
A/Blue-winged Teal/Texas/AH0040170S.1.A/2015 H3N8 | PB2
A/green-winged_teal/Alberta/431/2017 H3N8 | PB2(2)
A/green-winged_teal/Alberta/431/2017 H3N8 | PB2
A/mallard/Alaska/ND0006301/2013 H3N8 | PB2
A/American_wigeon/California/COL041/2014 H5N2 | PB2
A/mallard/Idaho/AH0008589/2015 H7N4 | PB2
A/cinnamon_teal/California/AH0079011/2016 H7N9 | PB2
A/pintail_duck/Ohio/16OS5000/2016 H3N8 | PB2
A/northern pintail/Alaska/568/2013 H7N3 | PB2
A/northern pintail/Alaska/856/2013 H10N7 | PB2
A/blue-winged_teal/Guatemala/CIP049H109-38/2012 H3N8 | PB2
A/Northern shoveler/Alaska/ND0006934/2013 H10N7 | PB2
A/mallard/California/57820/2015 H11N9 | PB2
A/northern_pintail/Alaska/619/2014 H7N4 | PB2
A/mallard/Southcentral_Alaska/12ML01535/2014 H2N3 | PB2
A/mallard/Alberta/532/2017 H8N4 | PB2
A/northern_shoveler/California/9228/2008 H4N6 | PB2
A/mallard/California/9573/2008 H4N6 | PB2
A/American black duck/New Brunswick/00612/2010 H10N7 | PB2
A/American black duck/New Brunswick/00487/2010 H4N8 | PB2
A/blue-winged_teal/Louisiana/AI09-4180/2009 H4N6 | PB2
A/blue-winged_teal/Texas/AI09-6182/2009 H6N2 | PB2
A/Glaucous-Winged Gull/Southcentral Alaska/17MB02917/2017 H3N2 | PB2
A/blue-winged teal/New Brunswick/03757/2009 H3N6 | PB2
A/American green-winged teal/Illinois/3054/2009 H1N2 | PB2
A/blue-winged teal/Texas/AI11-3181/2011 H4N2 | PB2
A/mallard/Minnesota/Sg-00133/2007 H4N6 | PB2
A/mallard.Maryland/708/2005 H3N2 | PB2
A/pintail/Alberta/166/2003 H3N6 | PB2
A/turkey/Virginia/505477-17/2007 H5N1 | PB2
A/mallard/Maryland/07OS882/2007 H3N6 | PB2
A/mallard/Minnesota/Sg-00831/2008 H3N8 | PB2
A/blue-winged_teal/Maryland/06MD462/2006 H12N2 | PB2
A/ruddy_turnstone/New_Jersey/Sg-00563/2008 H10N7 | PB2
A/ruddy turnstone/New Jersey/AI11-1968/2011 H7N3 | PB2
A/mallard/Wisconsin/10OS3067/2010 H4N2 | PB2
A/American green-winged teal/Ohio/12OS2256/2012 H6N5 | PB2
A/mallard/California/1492/2010 H6N4 | PB2
A/northern shoveler/Guatemala/CIP049H104-69/2011 H11N3 | PB2
A/American green-winged teal/Illinois/14OS2079/2014 H4N8 | PB2
A/blue-winged teal/Illinois/12OS2951/2012 H11N3 | PB2
A/mallard/Ohio/11OS2001/2011 H1N1 | PB2
A/blue-winged teal/Guatemala/CIP049H113-07/2013 H14N3 | PB2
A/northern shoveler/Mississippi/11OS5812/2011 H11N9 | PB2
A/mallard/Illinois/11OS5576/2011 H5N3 | PB2
A/northern shoveler/Ohio/12OS5647/2012 H2N3 | PB2
A/mallard/Minnesota/AH0059608/2015 H3N8 | PB2
A/green-Winged teal/Ohio/15OS4704/2015 H4N6 | PB2
A/blue-winged teal/Ohio/13OS2104/2013 H2N3 | PB2
A/blue-winged teal/Guatemala/CIP049H109-14/2012 H3N3 | PB2
A/blue-winged teal/Guatemala/CIP049H113-53/2013 H5N3 | PB2
99
99
99
99
98
99
99
99
96
99
99
97
97
99
98
98
99
97
98
97
99
0.010

B. PB1

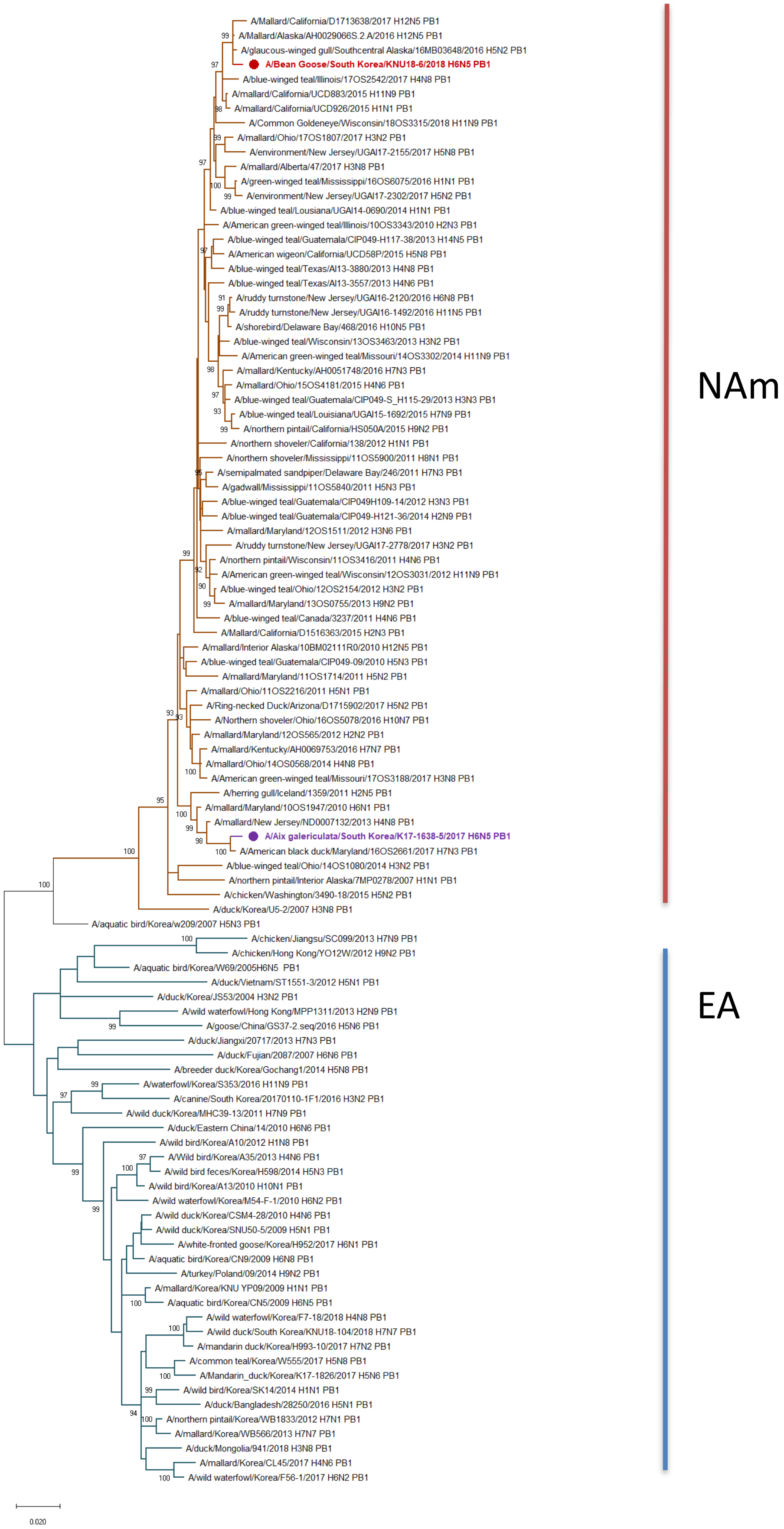

A/Mallard/California/D1713638/2017 H12N5 PB1
A/Mallard/Alaska/AH0029066S.2.A/2016 H12N5 PB1
A/glaucous-winged gull/Southcentral Alaska/16MB03648/2016 H5N2 PB1
A/Bean Goose/South Korea/KNU18-6/2018 H6N5 PB1
A/blue-winged teal/Illinois/17OS2542/2017 H4N8 PB1
A/mallard/California/UCD883/2015 H11N9 PB1
A/mallard/California/UCD926/2015 H1N1 PB1
A/Common Goldeneye/Wisconsin/18OS3315/2018 H11N9 PB1
A/mallard/Ohio/17OS1807/2017 H3N2 PB1
A/environment/New Jersey/UGAI17-2155/2017 H5N8 PB1
A/mallard/Alberta/47/2017 H3N8 PB1
A/green-winged teal/Mississippi/16OS6075/2016 H1N1 PB1
A/environment/New Jersey/UGAI17-2302/2017 H5N2 PB1
A/blue-winged teal/Lousiana/UGAI14-0690/2014 H1N1 PB1
A/American green-winged teal/Illinois/10OS3343/2010 H2N3 PB1
A/blue-winged teal/Guatemala/CIP049-H117-38/2013 H14N5 PB1
A/American wigeon/California/UCD58P/2015 H5N8 PB1
A/blue-winged teal/Texas/AI13-3880/2013 H4N8 PB1
A/blue-winged teal/Texas/AI13-3557/2013 H4N6 PB1
A/ruddy turnstone/New Jersey/UGAI16-2120/2016 H6N8 PB1
A/ruddy turnstone/New Jersey/UGAI16-1492/2016 H11N5 PB1
A/shorebird/Delaware Bay/468/2016 H10N5 PB1
A/blue-winged teal/Wisconsin/13OS3463/2013 H3N2 PB1
A/American green-winged teal/Missouri/14OS3302/2014 H11N9 PB1
A/mallard/Kentucky/AH0051748/2016 H7N3 PB1
A/mallard/Ohio/15OS4181/2015 H4N6 PB1
A/blue-winged teal/Guatemala/CIP049-S_H115-29/2013 H3N3 PB1
A/blue-winged teal/Louisiana/UGAI15-1692/2015 H7N9 PB1
A/northern pintail/California/HS050A/2015 H9N2 PB1
A/northern shoveler/California/138/2012 H1N1 PB1
A/northern shoveler/Mississippi/11OS5900/2011 H8N1 PB1
A/semipalmated sandpiper/Delaware Bay/246/2011 H7N3 PB1
A/gadwall/Mississippi/11OS5840/2011 H5N3 PB1
A/blue-winged teal/Guatemala/CIP049H109-14/2012 H3N3 PB1
A/blue-winged teal/Guatemala/CIP049-H121-36/2014 H2N9 PB1
A/mallard/Maryland/12OS1511/2012 H3N6 PB1
A/ruddy turnstone/New Jersey/UGAI17-2778/2017 H3N2 PB1
A/northern pintail/Wisconsin/11OS3416/2011 H4N6 PB1
A/American green-winged teal/Wisconsin/12OS3031/2012 H11N9 PB1
A/blue-winged teal/Ohio/12OS2154/2012 H3N2 PB1
A/mallard/Maryland/13OS0755/2013 H9N2 PB1
A/blue-winged teal/Canada/3237/2011 H4N6 PB1
A/Mallard/California/D1516363/2015 H2N3 PB1
A/mallard/Interior Alaska/10BM02111R0/2010 H12N5 PB1
A/blue-winged teal/Guatemala/CIP049-09/2010 H5N3 PB1
A/mallard/Maryland/11OS1714/2011 H5N2 PB1
A/mallard/Ohio/11OS2216/2011 H5N1 PB1
A/Ring-necked Duck/Arizona/D1715902/2017 H5N2 PB1
A/Northern shoveler/Ohio/16OS5078/2016 H10N7 PB1
A/mallard/Maryland/12OS565/2012 H2N2 PB1
A/mallard/Kentucky/AH0069753/2016 H7N7 PB1
A/mallard/Ohio/14OS0568/2014 H4N8 PB1
A/American green-winged teal/Missouri/17OS3188/2017 H3N8 PB1
A/herring gull/Iceland/1359/2011 H2N5 PB1
A/mallard/Maryland/10OS1947/2010 H6N1 PB1
A/mallard/New Jersey/ND0007132/2013 H4N8 PB1
A/Aix galericulata/South Korea/K17-1638-5/2017 H6N5 PB1
A/American black duck/Maryland/16OS2661/2017 H7N3 PB1
A/blue-winged teal/Ohio/14OS1080/2014 H3N2 PB1
A/northern pintail/Interior Alaska/7MP0278/2007 H1N1 PB1
A/chicken/Washington/3490-18/2015 H5N2 PB1
A/duck/Korea/U5-2/2007 H3N8 PB1
A/aquatic bird/Korea/w209/2007 H5N3 PB1
A/chicken/Jiangsu/SC099/2013 H7N9 PB1
A/chicken/Hong Kong/YO12W/2012 H9N2 PB1
A/aquatic bird/Korea/W69/2005H6N5 PB1
A/duck/Vietnam/ST1551-3/2012 H5N1 PB1
A/duck/Korea/JS53/2004 H3N2 PB1
A/wild waterfowl/Hong Kong/MPP1311/2013 H2N9 PB1
A/goose/China/GS37-2.seq/2016 H5N6 PB1
A/duck/Jiangxi/20717/2013 H7N3 PB1
A/duck/Fujian/2087/2007 H6N6 PB1
A/breeder duck/Korea/Gochang1/2014 H5N8 PB1
A/waterfowl/Korea/S353/2016 H11N9 PB1
A/canine/South Korea/20170110-1F1/2016 H3N2 PB1
A/wild duck/Korea/MHC39-13/2011 H7N9 PB1
A/duck/Eastern China/14/2010 H6N6 PB1
A/wild bird/Korea/A10/2012 H1N8 PB1
A/Wild bird/Korea/A35/2013 H4N6 PB1
A/wild bird feces/Korea/H598/2014 H5N3 PB1
A/wild bird/Korea/A13/2010 H10N1 PB1
A/wild waterfowl/Korea/M54-F-1/2010 H6N2 PB1
A/wild duck/Korea/CSM4-28/2010 H4N6 PB1
A/wild duck/Korea/SNU50-5/2009 H5N1 PB1
A/white-fronted goose/Korea/H952/2017 H6N1 PB1
A/aquatic bird/Korea/CN9/2009 H6N8 PB1
A/turkey/Poland/09/2014 H9N2 PB1
A/mallard/Korea/KNU YP09/2009 H1N1 PB1
A/aquatic bird/Korea/CN5/2009 H6N5 PB1
A/wild waterfowl/Korea/F7-18/2018 H4N8 PB1
A/wild duck/South Korea/KNU18-104/2018 H7N7 PB1
A/mandarin duck/Korea/H993-10/2017 H7N2 PB1
A/common teal/Korea/W555/2017 H5N8 PB1
A/Mandarin_duck/Korea/K17-1826/2017 H5N6 PB1
A/wild bird/Korea/SK14/2014 H1N1 PB1
A/duck/Bangladesh/28250/2016 H5N1 PB1
A/northern pintail/Korea/WB1833/2012 H7N1 PB1
A/mallard/Korea/WB566/2013 H7N7 PB1
A/duck/Mongolia/941/2018 H3N8 PB1
A/mallard/Korea/CL45/2017 H4N6 PB1
A/wild waterfowl/Korea/F56-1/2017 H6N2 PB1
NAm
EA
0.020

b. PB1

NAm

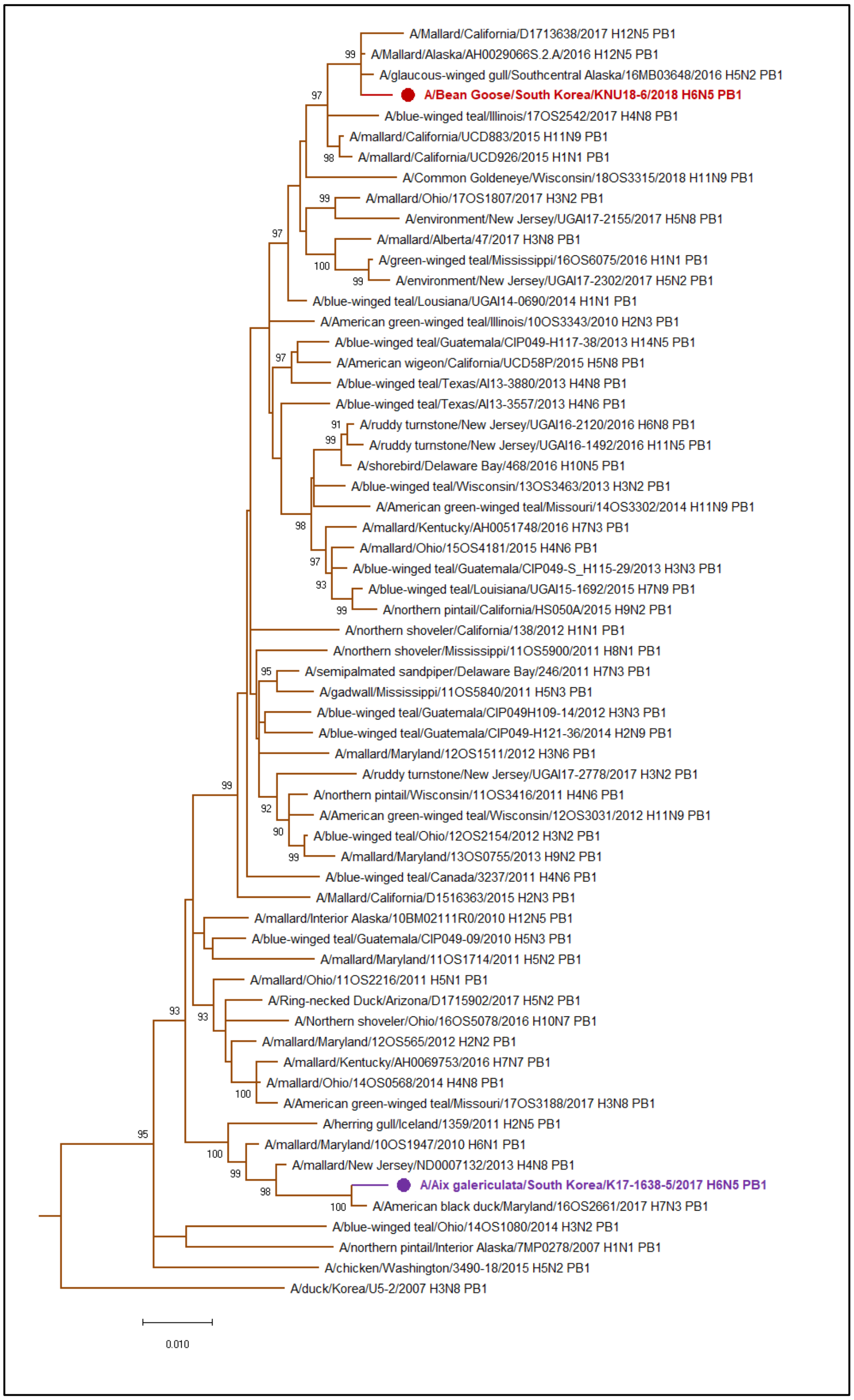
A/Mallard/California/D1713638/2017 H12N5 PB1
A/Mallard/Alaska/AH0029066S.2.A/2016 H12N5 PB1
A/glaucous-winged gull/Southcentral Alaska/16MB03648/2016 H5N2 PB1
A/Bean Goose/South Korea/KNU18-6/2018 H6N5 PB1
A/blue-winged teal/Illinois/17OS2542/2017 H4N8 PB1
A/mallard/California/UCD883/2015 H11N9 PB1
A/mallard/California/UCD926/2015 H1N1 PB1
A/Common Goldeneye/Wisconsin/18OS3315/2018 H11N9 PB1
A/mallard/Ohio/17OS1807/2017 H3N2 PB1
A/environment/New Jersey/UGAI17-2155/2017 H5N8 PB1
A/mallard/Alberta/47/2017 H3N8 PB1
A/green-winged teal/Mississippi/16OS6075/2016 H1N1 PB1
A/environment/New Jersey/UGAI17-2302/2017 H5N2 PB1
A/blue-winged teal/Lousiana/UGAI14-0690/2014 H1N1 PB1
A/American green-winged teal/Illinois/10OS3343/2010 H2N3 PB1
A/blue-winged teal/Guatemala/CIP049-H117-38/2013 H14N5 PB1
A/American wigeon/California/UCD58P/2015 H5N8 PB1
A/blue-winged teal/Texas/AI13-3880/2013 H4N8 PB1
A/blue-winged teal/Texas/AI13-3557/2013 H4N6 PB1
A/ruddy turnstone/New Jersey/UGAI16-2120/2016 H6N8 PB1
A/ruddy turnstone/New Jersey/UGAI16-1492/2016 H11N5 PB1
A/shorebird/Delaware Bay/468/2016 H10N5 PB1
A/blue-winged teal/Wisconsin/13OS3463/2013 H3N2 PB1
A/American green-winged teal/Missouri/14OS3302/2014 H11N9 PB1
A/mallard/Kentucky/AH0051748/2016 H7N3 PB1
A/mallard/Ohio/15OS4181/2015 H4N6 PB1
A/blue-winged teal/Guatemala/CIP049-S_H115-29/2013 H3N3 PB1
A/blue-winged teal/Louisiana/UGAI15-1692/2015 H7N9 PB1
A/northern pintail/California/HS050A/2015 H9N2 PB1
A/northern shoveler/California/138/2012 H1N1 PB1
A/northern shoveler/Mississippi/11OS5900/2011 H8N1 PB1
A/semipalmated sandpiper/Delaware Bay/246/2011 H7N3 PB1
A/gadwall/Mississippi/11OS5840/2011 H5N3 PB1
A/blue-winged teal/Guatemala/CIP049H109-14/2012 H3N3 PB1
A/blue-winged teal/Guatemala/CIP049-H121-36/2014 H2N9 PB1
A/mallard/Maryland/12OS1511/2012 H3N6 PB1
A/ruddy turnstone/New Jersey/UGAI17-2778/2017 H3N2 PB1
A/northern pintail/Wisconsin/11OS3416/2011 H4N6 PB1
A/American green-winged teal/Wisconsin/12OS3031/2012 H11N9 PB1
A/blue-winged teal/Ohio/12OS2154/2012 H3N2 PB1
A/mallard/Maryland/13OS0755/2013 H9N2 PB1
A/blue-winged teal/Canada/3237/2011 H4N6 PB1
A/Mallard/California/D1516363/2015 H2N3 PB1
A/mallard/Interior Alaska/10BM02111R0/2010 H12N5 PB1
A/blue-winged teal/Guatemala/CIP049-09/2010 H5N3 PB1
A/mallard/Maryland/11OS1714/2011 H5N2 PB1
A/mallard/Ohio/11OS2216/2011 H5N1 PB1
A/Ring-necked Duck/Arizona/D1715902/2017 H5N2 PB1
A/Northern shoveler/Ohio/16OS5078/2016 H10N7 PB1
A/mallard/Maryland/12OS565/2012 H2N2 PB1
A/mallard/Kentucky/AH0069753/2016 H7N7 PB1
A/mallard/Ohio/14OS0568/2014 H4N8 PB1
A/American green-winged teal/Missouri/17OS3188/2017 H3N8 PB1
A/herring gull/Iceland/1359/2011 H2N5 PB1
A/mallard/Maryland/10OS1947/2010 H6N1 PB1
A/mallard/New Jersey/ND0007132/2013 H4N8 PB1
A/Aix galericulata/South Korea/K17-1638-5/2017 H6N5 PB1
A/American black duck/Maryland/16OS2661/2017 H7N3 PB1
A/blue-winged teal/Ohio/14OS1080/2014 H3N2 PB1
A/northern pintail/Interior Alaska/7MP0278/2007 H1N1 PB1
A/chicken/Washington/3490-18/2015 H5N2 PB1
A/duck/Korea/U5-2/2007 H3N8 PB1
0.010

C. PA

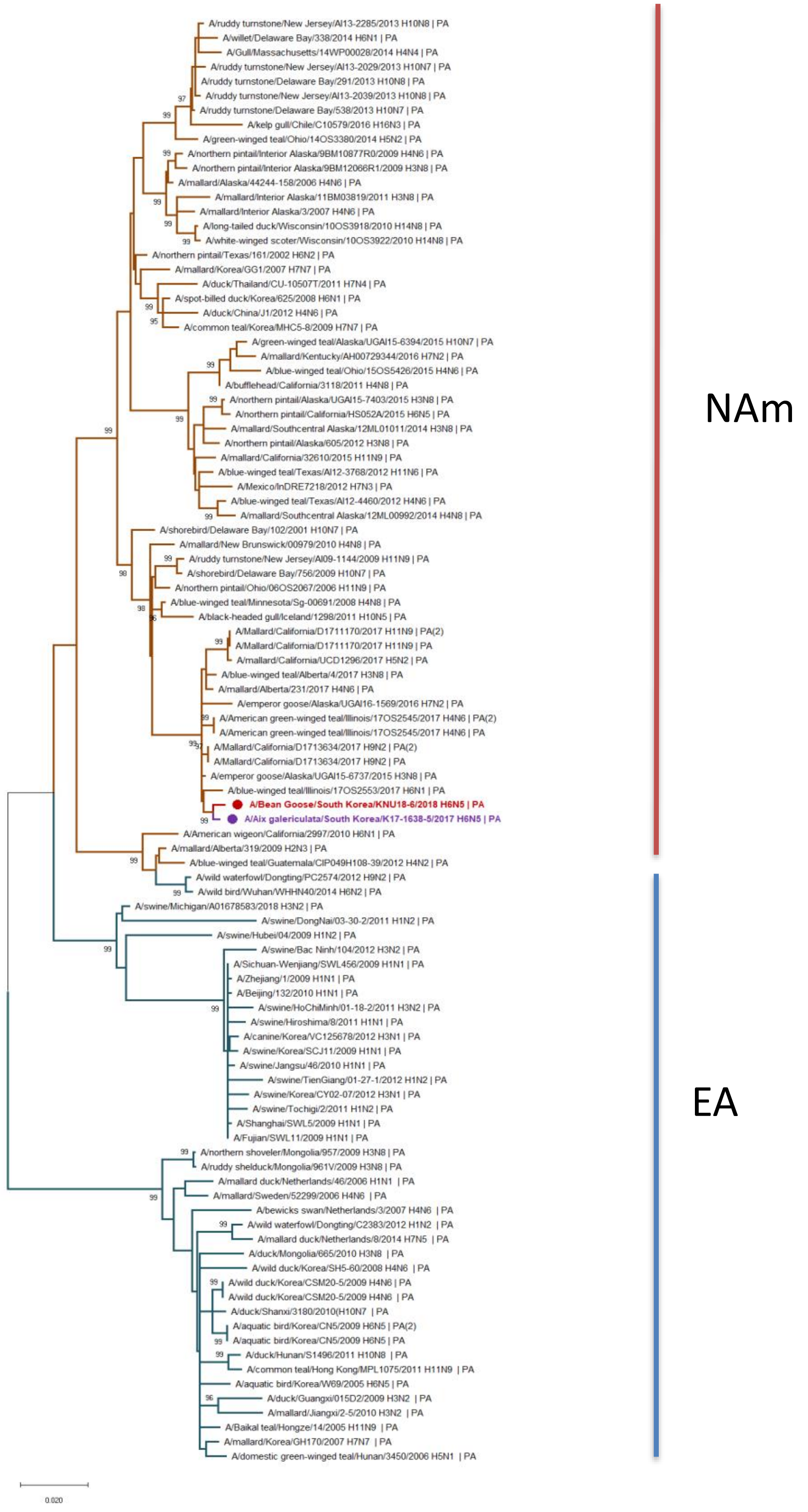

A/ruddy turnstone/New Jersey/AI13-2285/2013 H10N8 | PA
A/willet/Delaware Bay/338/2014 H6N1 | PA
A/Gull/Massachusetts/14WP00028/2014 H4N4 | PA
A/ruddy turnstone/New Jersey/AI13-2029/2013 H10N7 | PA
A/ruddy turnstone/Delaware Bay/291/2013 H10N8 | PA
A/ruddy turnstone/New Jersey/AI13-2039/2013 H10N8 | PA
A/ruddy turnstone/Delaware Bay/538/2013 H10N7 | PA
A/kelp gull/Chile/C10579/2016 H16N3 | PA
A/green-winged teal/Ohio/14OS3380/2014 H5N2 | PA
A/northern pintail/Interior Alaska/9BM10877R0/2009 H4N6 | PA
A/northern pintail/Interior Alaska/9BM12066R1/2009 H3N8 | PA
A/mallard/Alaska/44244-158/2006 H4N6 | PA
A/mallard/Interior Alaska/11BM03819/2011 H3N8 | PA
A/mallard/Interior Alaska/3/2007 H4N6 | PA
A/long-tailed duck/Wisconsin/10OS3918/2010 H14N8 | PA
A/white-winged scoter/Wisconsin/10OS3922/2010 H14N8 | PA
A/northern pintail/Texas/161/2002 H6N2 | PA
A/mallard/Korea/GG1/2007 H7N7 | PA
A/duck/Thailand/CU-10507T/2011 H7N4 | PA
A/spot-billed duck/Korea/625/2008 H6N1 | PA
A/duck/China/J1/2012 H4N6 | PA
A/common teal/Korea/MHC5-8/2009 H7N7 | PA
A/green-winged teal/Alaska/UGAI15-6394/2015 H10N7 | PA
A/mallard/Kentucky/AH00729344/2016 H7N2 | PA
A/blue-winged teal/Ohio/15OS5426/2015 H4N6 | PA
A/bufflehead/California/3118/2011 H4N8 | PA
A/northern pintail/Alaska/UGAI15-7403/2015 H3N8 | PA
A/northern pintail/California/HS052A/2015 H6N5 | PA
A/mallard/Southcentral Alaska/12ML01011/2014 H3N8 | PA
A/northern pintail/Alaska/605/2012 H3N8 | PA
A/mallard/California/32610/2015 H11N9 | PA
A/blue-winged teal/Texas/AI12-3768/2012 H11N6 | PA
A/Mexico/InDRE7218/2012 H7N3 | PA
A/blue-winged teal/Texas/AI12-4460/2012 H4N6 | PA
A/mallard/Southcentral Alaska/12ML00992/2014 H4N8 | PA
A/shorebird/Delaware Bay/102/2001 H10N7 | PA
A/mallard/New Brunswick/00979/2010 H4N8 | PA
A/ruddy turnstone/New Jersey/AI09-1144/2009 H11N9 | PA
A/shorebird/Delaware Bay/756/2009 H10N7 | PA
A/northern pintail/Ohio/06OS2067/2006 H11N9 | PA
A/blue-winged teal/Minnesota/Sg-00691/2008 H4N8 | PA
A/black-headed gull/Iceland/1298/2011 H10N5 | PA
A/Mallard/California/D1711170/2017 H11N9 | PA(2)
A/Mallard/California/D1711170/2017 H11N9 | PA
A/mallard/California/UCD1296/2017 H5N2 | PA
A/blue-winged teal/Alberta/4/2017 H3N8 | PA
A/mallard/Alberta/231/2017 H4N6 | PA
A/emperor goose/Alaska/UGAI16-1569/2016 H7N2 | PA
A/American green-winged teal/Illinois/17OS2545/2017 H4N6 | PA(2)
A/American green-winged teal/Illinois/17OS2545/2017 H4N6 | PA
A/Mallard/California/D1713634/2017 H9N2 | PA(2)
A/Mallard/California/D1713634/2017 H9N2 | PA
A/emperor goose/Alaska/UGAI15-6737/2015 H3N8 | PA
A/blue-winged teal/Illinois/17OS2553/2017 H6N1 | PA
A/Bean Goose/South Korea/KNU18-6/2018 H6N5 | PA
A/Aix galericulata/South Korea/K17-1638-5/2017 H6N5 | PA
A/American wigeon/California/2997/2010 H6N1 | PA
A/mallard/Alberta/319/2009 H2N3 | PA
A/blue-winged teal/Guatemala/CIP049H108-39/2012 H4N2 | PA
A/wild waterfowl/Dongting/PC2574/2012 H9N2 | PA
A/wild bird/Wuhan/WHHN40/2014 H6N2 | PA
A/swine/Michigan/A01678583/2018 H3N2 | PA
A/swine/DongNai/03-30-2/2011 H1N2 | PA
A/swine/Hubei/04/2009 H1N2 | PA
A/swine/Bac Ninh/104/2012 H3N2 | PA
A/Sichuan-Wenjiang/SWL456/2009 H1N1 | PA
A/Zhejiang/1/2009 H1N1 | PA
A/Beijing/132/2010 H1N1 | PA
A/swine/HoChiMinh/01-18-2/2011 H3N2 | PA
A/swine/Hiroshima/8/2011 H1N1 | PA
A/canine/Korea/VC125678/2012 H3N1 | PA
A/swine/Korea/SCJ11/2009 H1N1 | PA
A/swine/Jangsu/46/2010 H1N1 | PA
A/swine/TienGiang/01-27-1/2012 H1N2 | PA
A/swine/Korea/CY02-07/2012 H3N1 | PA
A/swine/Tochigi/2/2011 H1N2 | PA
A/Shanghai/SWL5/2009 H1N1 | PA
A/Fujian/SWL11/2009 H1N1 | PA
A/northern shoveler/Mongolia/957/2009 H3N8 | PA
A/ruddy shelduck/Mongolia/961V/2009 H3N8 | PA
A/mallard duck/Netherlands/46/2006 H1N1 | PA
A/mallard/Sweden/52299/2006 H4N6 | PA
A/bewicks swan/Netherlands/3/2007 H4N6 | PA
A/wild waterfowl/Dongting/C2383/2012 H1N2 | PA
A/mallard duck/Netherlands/8/2014 H7N5 | PA
A/duck/Mongolia/665/2010 H3N8 | PA
A/wild duck/Korea/SH5-60/2008 H4N6 | PA
A/wild duck/Korea/CSM20-5/2009 H4N6 | PA
A/wild duck/Korea/CSM20-5/2009 H4N6 | PA
A/duck/Shanxi/3180/2010(H10N7 | PA
A/aquatic bird/Korea/CN5/2009 H6N5 | PA(2)
A/aquatic bird/Korea/CN5/2009 H6N5 | PA
A/duck/Hunan/S1496/2011 H10N8 | PA
A/common teal/Hong Kong/MPL1075/2011 H11N9 | PA
A/aquatic bird/Korea/W69/2005 H6N5 | PA
A/duck/Guangxi/015D2/2009 H3N2 | PA
A/mallard/Jiangxi/2-5/2010 H3N2 | PA
A/Baikal teal/Hongze/14/2005 H11N9 | PA
A/mallard/Korea/GH170/2007 H7N7 | PA
A/domestic green-winged teal/Hunan/3450/2006 H5N1 | PA
NAm
EA
0.020

c. PA

NAm

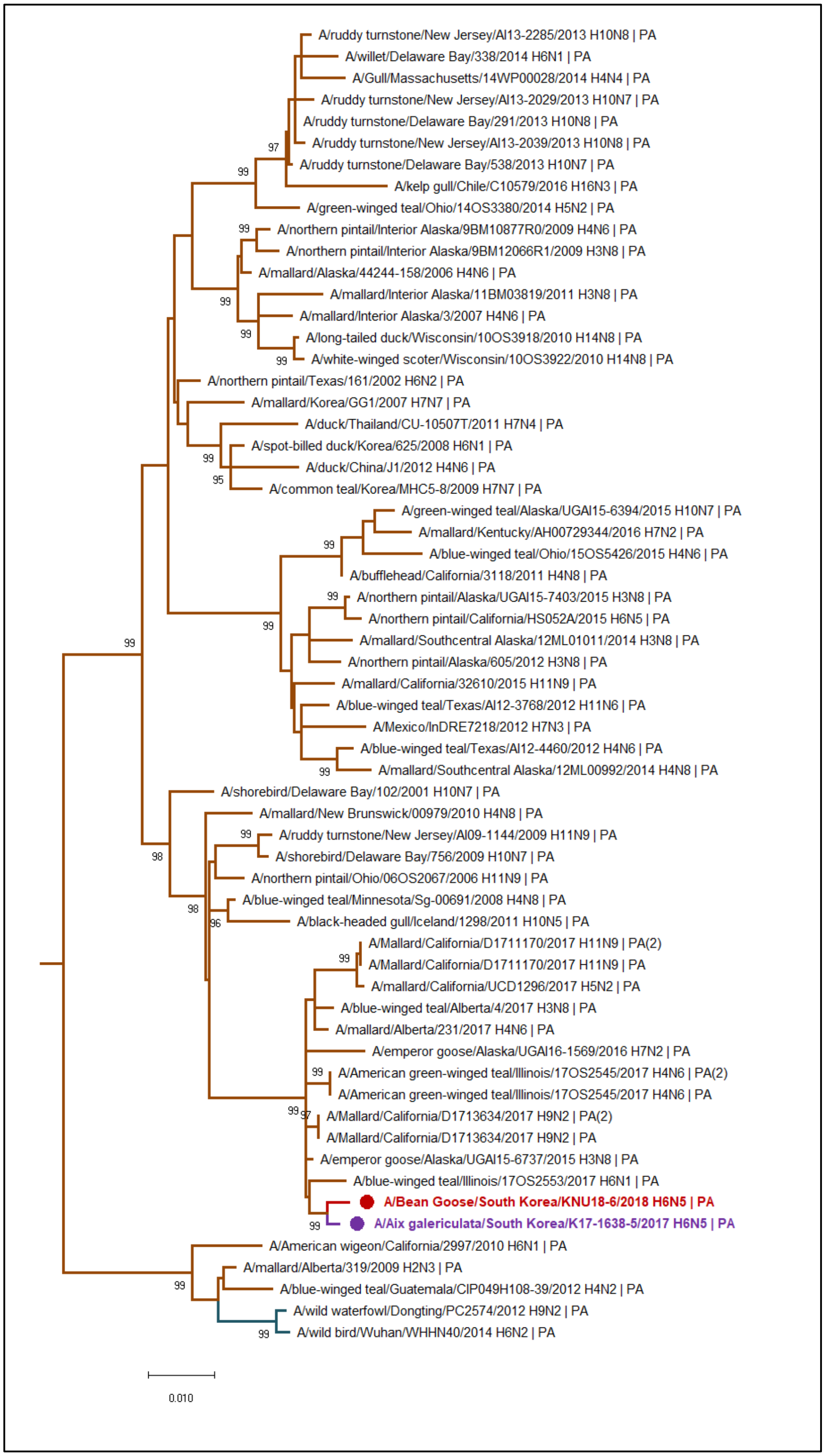
A/ruddy turnstone/New Jersey/AI13-2285/2013 H10N8 | PA
A/willet/Delaware Bay/338/2014 H6N1 | PA
A/Gull/Massachusetts/14WP00028/2014 H4N4 | PA
A/ruddy turnstone/New Jersey/AI13-2029/2013 H10N7 | PA
A/ruddy turnstone/Delaware Bay/291/2013 H10N8 | PA
A/ruddy turnstone/New Jersey/AI13-2039/2013 H10N8 | PA
A/ruddy turnstone/Delaware Bay/538/2013 H10N7 | PA
A/kelp gull/Chile/C10579/2016 H16N3 | PA
A/green-winged teal/Ohio/14OS3380/2014 H5N2 | PA
A/northern pintail/Interior Alaska/9BM10877R0/2009 H4N6 | PA
A/northern pintail/Interior Alaska/9BM12066R1/2009 H3N8 | PA
A/mallard/Alaska/44244-158/2006 H4N6 | PA
A/mallard/Interior Alaska/11BM03819/2011 H3N8 | PA
A/mallard/Interior Alaska/3/2007 H4N6 | PA
A/long-tailed duck/Wisconsin/10OS3918/2010 H14N8 | PA
A/white-winged scoter/Wisconsin/10OS3922/2010 H14N8 | PA
A/northern pintail/Texas/161/2002 H6N2 | PA
A/mallard/Korea/GG1/2007 H7N7 | PA
A/duck/Thailand/CU-10507T/2011 H7N4 | PA
A/spot-billed duck/Korea/625/2008 H6N1 | PA
A/duck/China/J1/2012 H4N6 | PA
A/common teal/Korea/MHC5-8/2009 H7N7 | PA
A/green-winged teal/Alaska/UGAI15-6394/2015 H10N7 | PA
A/mallard/Kentucky/AH00729344/2016 H7N2 | PA
A/blue-winged teal/Ohio/15OS5426/2015 H4N6 | PA
A/bufflehead/California/3118/2011 H4N8 | PA
A/northern pintail/Alaska/UGAI15-7403/2015 H3N8 | PA
A/northern pintail/California/HS052A/2015 H6N5 | PA
A/mallard/Southcentral Alaska/12ML01011/2014 H3N8 | PA
A/northern pintail/Alaska/605/2012 H3N8 | PA
A/mallard/California/32610/2015 H11N9 | PA
A/blue-winged teal/Texas/AI12-3768/2012 H11N6 | PA
A/Mexico/InDRE7218/2012 H7N3 | PA
A/blue-winged teal/Texas/AI12-4460/2012 H4N6 | PA
A/mallard/Southcentral Alaska/12ML00992/2014 H4N8 | PA
A/shorebird/Delaware Bay/102/2001 H10N7 | PA
A/mallard/New Brunswick/00979/2010 H4N8 | PA
A/ruddy turnstone/New Jersey/AI09-1144/2009 H11N9 | PA
A/shorebird/Delaware Bay/756/2009 H10N7 | PA
A/northern pintail/Ohio/06OS2067/2006 H11N9 | PA
A/blue-winged teal/Minnesota/Sg-00691/2008 H4N8 | PA
A/black-headed gull/Iceland/1298/2011 H10N5 | PA
A/Mallard/California/D1711170/2017 H11N9 | PA(2)
A/Mallard/California/D1711170/2017 H11N9 | PA
A/mallard/California/UCD1296/2017 H5N2 | PA
A/blue-winged teal/Alberta/4/2017 H3N8 | PA
A/mallard/Alberta/231/2017 H4N6 | PA
A/emperor goose/Alaska/UGAI16-1569/2016 H7N2 | PA
A/American green-winged teal/Illinois/17OS2545/2017 H4N6 | PA(2)
A/American green-winged teal/Illinois/17OS2545/2017 H4N6 | PA
A/Mallard/California/D1713634/2017 H9N2 | PA(2)
A/Mallard/California/D1713634/2017 H9N2 | PA
A/emperor goose/Alaska/UGAI15-6737/2015 H3N8 | PA
A/blue-winged teal/Illinois/17OS2553/2017 H6N1 | PA
A/Bean Goose/South Korea/KNU18-6/2018 H6N5 | PA
A/Aix galericulata/South Korea/K17-1638-5/2017 H6N5 | PA
A/American wigeon/California/2997/2010 H6N1 | PA
A/mallard/Alberta/319/2009 H2N3 | PA
A/blue-winged teal/Guatemala/CIP049H108-39/2012 H4N2 | PA
A/wild waterfowl/Dongting/PC2574/2012 H9N2 | PA
A/wild bird/Wuhan/WHHN40/2014 H6N2 | PA
0.010

D. HA

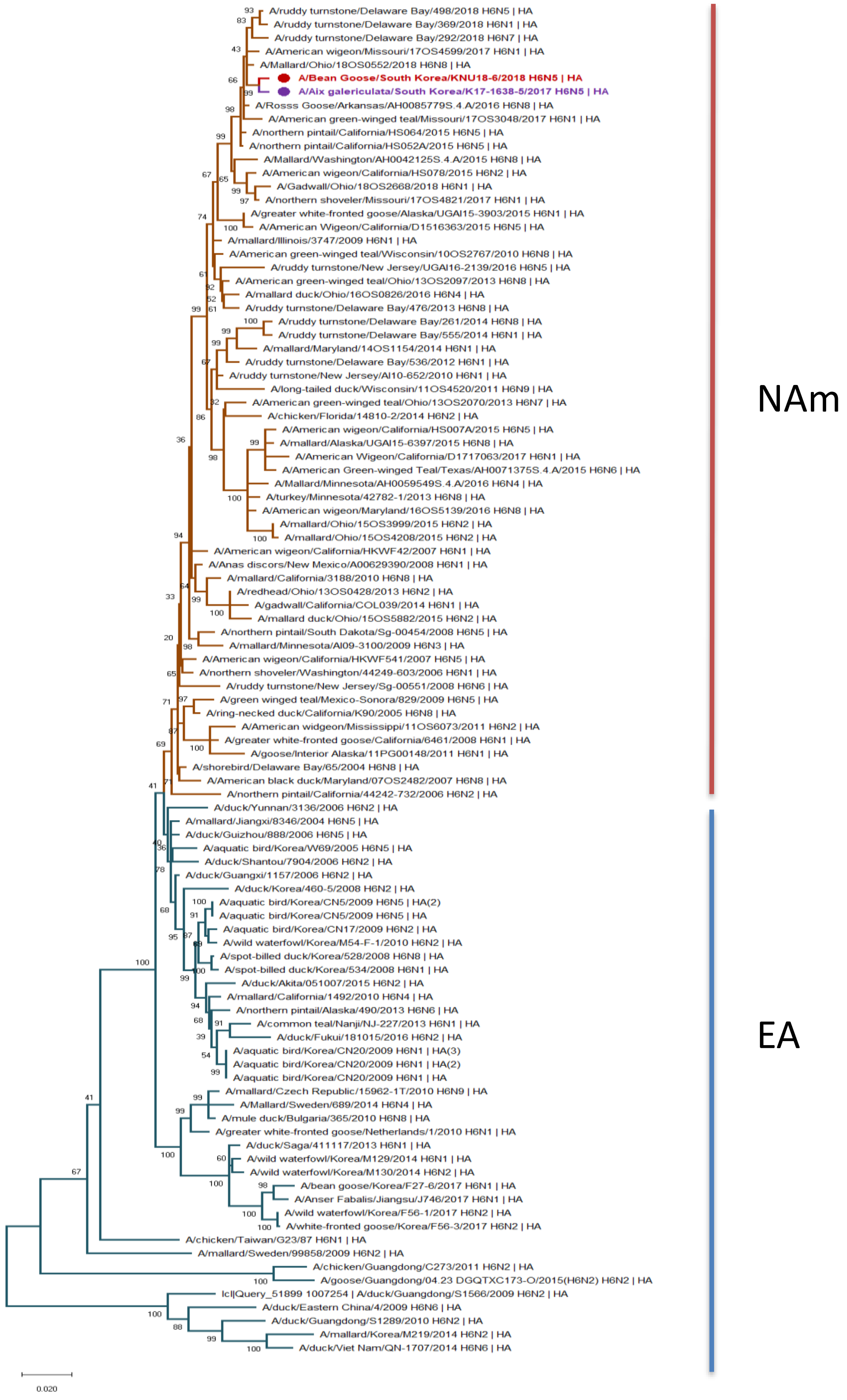
A/ruddy turnstone/Delaware Bay/498/2018 H6N5 | HA
A/ruddy turnstone/Delaware Bay/369/2018 H6N1 | HA
A/ruddy turnstone/Delaware Bay/292/2018 H6N7 | HA
A/American wigeon/Missouri/17OS4599/2017 H6N1 | HA
A/Mallard/Ohio/18OS0552/2018 H6N8 | HA
A/Bean Goose/South Korea/KNU18-6/2018 H6N5 | HA
A/Aix galericulata/South Korea/K17-1638-5/2017 H6N5 | HA
A/Rosss Goose/Arkansas/AH0085779S.4.A/2016 H6N8 | HA
A/American green-winged teal/Missouri/17OS3048/2017 H6N1 | HA
A/northern pintail/California/HS064/2015 H6N5 | HA
A/northern pintail/California/HS052A/2015 H6N5 | HA
A/Mallard/Washington/AH0042125S.4.A/2015 H6N8 | HA
A/American wigeon/California/HS078/2015 H6N2 | HA
A/Gadwall/Ohio/18OS2668/2018 H6N1 | HA
A/northern shoveler/Missouri/17OS4821/2017 H6N1 | HA
A/greater white-fronted goose/Alaska/UGAI15-3903/2015 H6N1 | HA
A/American Wigeon/California/D1516363/2015 H6N5 | HA
A/mallard/Illinois/3747/2009 H6N1 | HA
A/American green-winged teal/Wisconsin/10OS2767/2010 H6N8 | HA
A/ruddy turnstone/New Jersey/UGAI16-2139/2016 H6N5 | HA
A/American green-winged teal/Ohio/13OS2097/2013 H6N8 | HA
A/mallard duck/Ohio/16OS0826/2016 H6N4 | HA
A/ruddy turnstone/Delaware Bay/476/2013 H6N8 | HA
A/ruddy turnstone/Delaware Bay/261/2014 H6N8 | HA
A/ruddy turnstone/Delaware Bay/555/2014 H6N1 | HA
A/mallard/Maryland/14OS1154/2014 H6N1 | HA
A/ruddy turnstone/Delaware Bay/536/2012 H6N1 | HA
A/ruddy turnstone/New Jersey/AI10-652/2010 H6N1 | HA
A/long-tailed duck/Wisconsin/11OS4520/2011 H6N9 | HA
A/American green-winged teal/Ohio/13OS2070/2013 H6N7 | HA
A/chicken/Florida/14810-2/2014 H6N2 | HA
A/American wigeon/California/HS007A/2015 H6N5 | HA
A/mallard/Alaska/UGAI15-6397/2015 H6N8 | HA
A/American Wigeon/California/D1717063/2017 H6N1 | HA
A/American Green-winged Teal/Texas/AH0071375S.4.A/2015 H6N6 | HA
A/Mallard/Minnesota/AH0059549S.4.A/2016 H6N4 | HA
A/turkey/Minnesota/42782-1/2013 H6N8 | HA
A/American wigeon/Maryland/16OS5139/2016 H6N8 | HA
A/mallard/Ohio/15OS3999/2015 H6N2 | HA
A/mallard/Ohio/15OS4208/2015 H6N2 | HA
A/American wigeon/California/HKWF42/2007 H6N1 | HA
A/Anas discors/New Mexico/A00629390/2008 H6N1 | HA
A/mallard/California/3188/2010 H6N8 | HA
A/redhead/Ohio/13OS0428/2013 H6N2 | HA
A/gadwall/California/COL039/2014 H6N1 | HA
A/mallard duck/Ohio/15OS5882/2015 H6N2 | HA
A/northern pintail/South Dakota/Sg-00454/2008 H6N5 | HA
A/mallard/Minnesota/AI09-3100/2009 H6N3 | HA
A/American wigeon/California/HKWF541/2007 H6N5 | HA
A/northern shoveler/Washington/44249-603/2006 H6N1 | HA
A/ruddy turnstone/New Jersey/Sg-00551/2008 H6N6 | HA
A/green winged teal/Mexico-Sonora/829/2009 H6N5 | HA
A/ring-necked duck/California/K90/2005 H6N8 | HA
A/American widgeon/Mississippi/11OS6073/2011 H6N2 | HA
A/greater white-fronted goose/California/6461/2008 H6N1 | HA
A/goose/Interior Alaska/11PG00148/2011 H6N1 | HA
A/shorebird/Delaware Bay/65/2004 H6N8 | HA
A/American black duck/Maryland/07OS2482/2007 H6N8 | HA
A/northern pintail/California/44242-732/2006 H6N2 | HA
A/duck/Yunnan/3136/2006 H6N2 | HA
A/mallard/Jiangxi/8346/2004 H6N5 | HA
A/duck/Guizhou/888/2006 H6N5 | HA
A/aquatic bird/Korea/W69/2005 H6N5 | HA
A/duck/Shantou/7904/2006 H6N2 | HA
A/duck/Guangxi/1157/2006 H6N2 | HA
A/duck/Korea/460-5/2008 H6N2 | HA
A/aquatic bird/Korea/CN5/2009 H6N5 | HA(2)
A/aquatic bird/Korea/CN5/2009 H6N5 | HA
A/aquatic bird/Korea/CN17/2009 H6N2 | HA
A/wild waterfowl/Korea/M54-F-1/2010 H6N2 | HA
A/spot-billed duck/Korea/528/2008 H6N8 | HA
A/spot-billed duck/Korea/534/2008 H6N1 | HA
A/duck/Akita/051007/2015 H6N2 | HA
A/mallard/California/1492/2010 H6N4 | HA
A/northern pintail/Alaska/490/2013 H6N6 | HA
A/common teal/Nanji/NJ-227/2013 H6N1 | HA
A/duck/Fukui/181015/2016 H6N2 | HA
A/aquatic bird/Korea/CN20/2009 H6N1 | HA(3)
A/aquatic bird/Korea/CN20/2009 H6N1 | HA(2)
A/aquatic bird/Korea/CN20/2009 H6N1 | HA
A/mallard/Czech Republic/15962-1T/2010 H6N9 | HA
A/Mallard/Sweden/689/2014 H6N4 | HA
A/mule duck/Bulgaria/365/2010 H6N8 | HA
A/greater white-fronted goose/Netherlands/1/2010 H6N1 | HA
A/duck/Saga/411117/2013 H6N1 | HA
A/wild waterfowl/Korea/M129/2014 H6N1 | HA
A/wild waterfowl/Korea/M130/2014 H6N2 | HA
A/bean goose/Korea/F27-6/2017 H6N1 | HA
A/Anser Fabalis/Jiangsu/J746/2017 H6N1 | HA
A/wild waterfowl/Korea/F56-1/2017 H6N2 | HA
A/white-fronted goose/Korea/F56-3/2017 H6N2 | HA
A/chicken/Taiwan/G23/87 H6N1 | HA
A/mallard/Sweden/99858/2009 H6N2 | HA
A/chicken/Guangdong/C273/2011 H6N2 | HA
A/goose/Guangdong/04.23 DGQTXC173-O/2015(H6N2) H6N2 | HA
lcl|Query_51899 1007254 | A/duck/Guangdong/S1566/2009 H6N2 | HA
A/duck/Eastern China/4/2009 H6N6 | HA
A/duck/Guangdong/S1289/2010 H6N2 | HA
A/mallard/Korea/M219/2014 H6N2 | HA
A/duck/Viet Nam/QN-1707/2014 H6N6 | HA
NAm
EA
0.020

d. HA
NAm

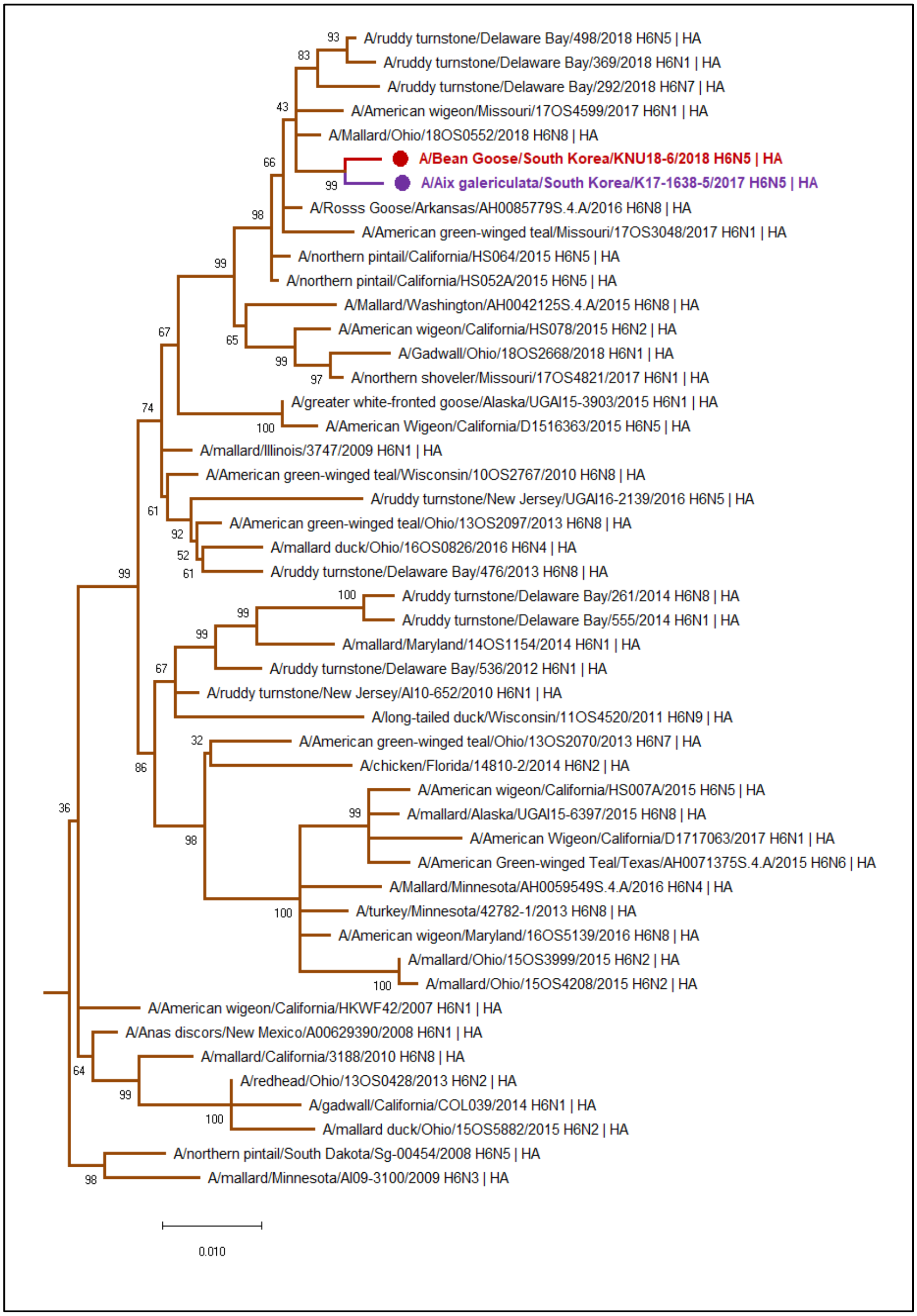
A/ruddy turnstone/Delaware Bay/498/2018 H6N5 | HA
A/ruddy turnstone/Delaware Bay/369/2018 H6N1 | HA
A/ruddy turnstone/Delaware Bay/292/2018 H6N7 | HA
A/American wigeon/Missouri/17OS4599/2017 H6N1 | HA
A/Mallard/Ohio/18OS0552/2018 H6N8 | HA
A/Bean Goose/South Korea/KNU18-6/2018 H6N5 | HA
A/Aix galericulata/South Korea/K17-1638-5/2017 H6N5 | HA
A/Rosss Goose/Arkansas/AH0085779S.4.A/2016 H6N8 | HA
A/American green-winged teal/Missouri/17OS3048/2017 H6N1 | HA
A/northern pintail/California/HS064/2015 H6N5 | HA
A/northern pintail/California/HS052A/2015 H6N5 | HA
A/Mallard/Washington/AH0042125S.4.A/2015 H6N8 | HA
A/American wigeon/California/HS078/2015 H6N2 | HA
A/Gadwall/Ohio/18OS2668/2018 H6N1 | HA
A/northern shoveler/Missouri/17OS4821/2017 H6N1 | HA
A/greater white-fronted goose/Alaska/UGAI15-3903/2015 H6N1 | HA
A/American Wigeon/California/D1516363/2015 H6N5 | HA
A/mallard/Illinois/3747/2009 H6N1 | HA
A/American green-winged teal/Wisconsin/10OS2767/2010 H6N8 | HA
A/ruddy turnstone/New Jersey/UGAI16-2139/2016 H6N5 | HA
A/American green-winged teal/Ohio/13OS2097/2013 H6N8 | HA
A/mallard duck/Ohio/16OS0826/2016 H6N4 | HA
A/ruddy turnstone/Delaware Bay/476/2013 H6N8 | HA
A/ruddy turnstone/Delaware Bay/261/2014 H6N8 | HA
A/ruddy turnstone/Delaware Bay/555/2014 H6N1 | HA
A/mallard/Maryland/14OS1154/2014 H6N1 | HA
A/ruddy turnstone/Delaware Bay/536/2012 H6N1 | HA
A/ruddy turnstone/New Jersey/AI10-652/2010 H6N1 | HA
A/long-tailed duck/Wisconsin/11OS4520/2011 H6N9 | HA
A/American green-winged teal/Ohio/13OS2070/2013 H6N7 | HA
A/chicken/Florida/14810-2/2014 H6N2 | HA
A/American wigeon/California/HS007A/2015 H6N5 | HA
A/mallard/Alaska/UGAI15-6397/2015 H6N8 | HA
A/American Wigeon/California/D1717063/2017 H6N1 | HA
A/American Green-winged Teal/Texas/AH0071375S.4.A/2015 H6N6 | HA
A/Mallard/Minnesota/AH0059549S.4.A/2016 H6N4 | HA
A/turkey/Minnesota/42782-1/2013 H6N8 | HA
A/American wigeon/Maryland/16OS5139/2016 H6N8 | HA
A/mallard/Ohio/15OS3999/2015 H6N2 | HA
A/mallard/Ohio/15OS4208/2015 H6N2 | HA
A/American wigeon/California/HKWF42/2007 H6N1 | HA
A/Anas discors/New Mexico/A00629390/2008 H6N1 | HA
A/mallard/California/3188/2010 H6N8 | HA
A/redhead/Ohio/13OS0428/2013 H6N2 | HA
A/gadwall/California/COL039/2014 H6N1 | HA
A/mallard duck/Ohio/15OS5882/2015 H6N2 | HA
A/northern pintail/South Dakota/Sg-00454/2008 H6N5 | HA
A/mallard/Minnesota/AI09-3100/2009 H6N3 | HA
0.010

E. NP

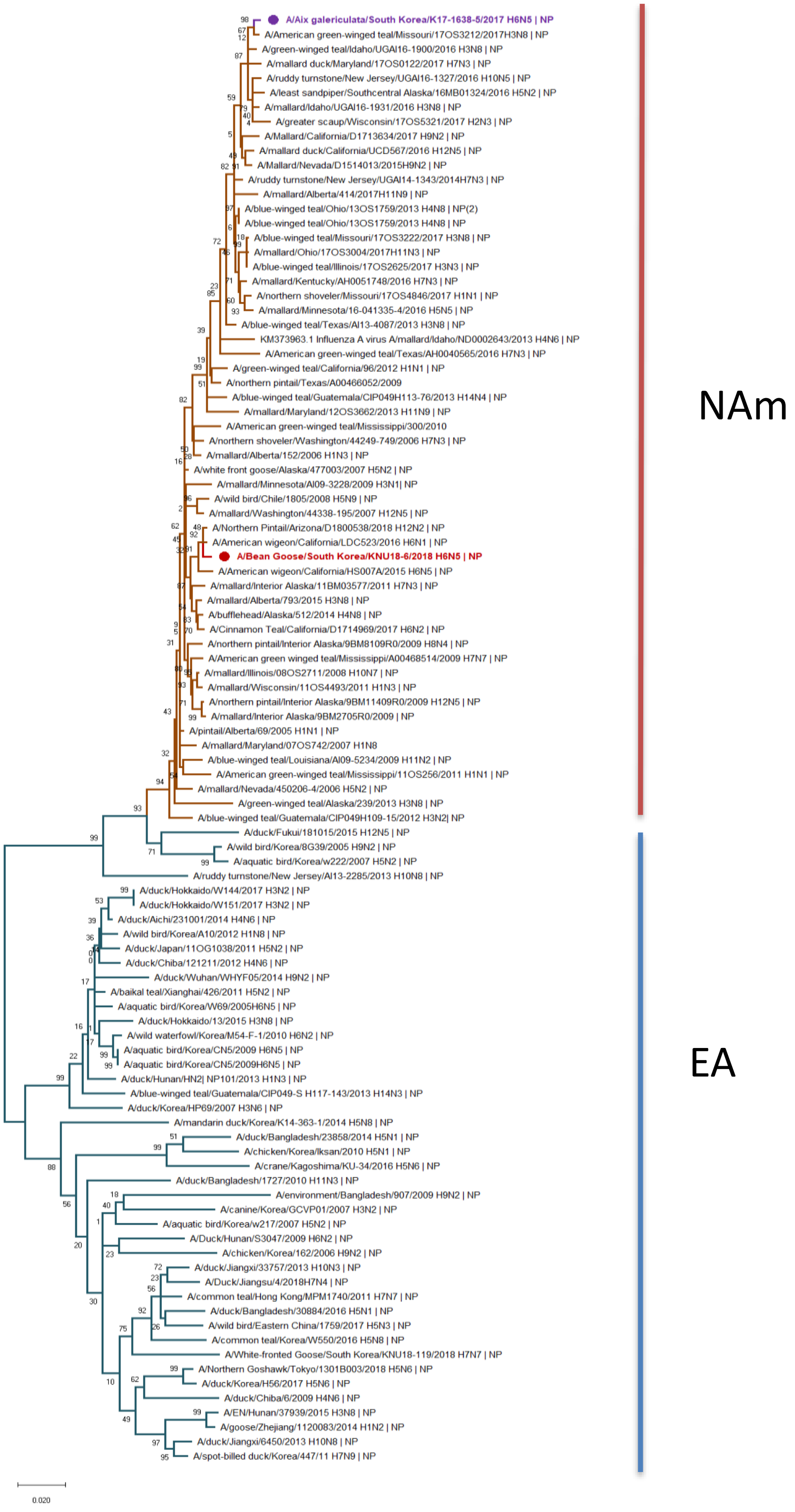

A/Aix galericulata/South Korea/K17-1638-5/2017 H6N5 | NP
A/American green-winged teal/Missouri/17OS3212/2017H3N8 | NP
A/green-winged teal/Idaho/UGAI16-1900/2016 H3N8 | NP
A/mallard duck/Maryland/17OS0122/2017 H7N3 | NP
A/ruddy turnstone/New Jersey/UGAI16-1327/2016 H10N5 | NP
A/least sandpiper/Southcentral Alaska/16MB01324/2016 H5N2 | NP
A/mallard/Idaho/UGAI16-1931/2016 H3N8 | NP
A/greater scaup/Wisconsin/17OS5321/2017 H2N3 | NP
A/Mallard/California/D1713634/2017 H9N2 | NP
A/mallard duck/California/UCD567/2016 H12N5 | NP
A/Mallard/Nevada/D1514013/2015H9N2 | NP
A/ruddy turnstone/New Jersey/UGAI14-1343/2014H7N3 | NP
A/mallard/Alberta/414/2017H11N9 | NP
A/blue-winged teal/Ohio/13OS1759/2013 H4N8 | NP(2)
A/blue-winged teal/Ohio/13OS1759/2013 H4N8 | NP
A/blue-winged teal/Missouri/17OS3222/2017 H3N8 | NP
A/mallard/Ohio/17OS3004/2017H11N3 | NP
A/blue-winged teal/Illinois/17OS2625/2017 H3N3 | NP
A/mallard/Kentucky/AH0051748/2016 H7N3 | NP
A/northern shoveler/Missouri/17OS4846/2017 H1N1 | NP
A/mallard/Minnesota/16-041335-4/2016 H5N5 | NP
A/blue-winged teal/Texas/AI13-4087/2013 H3N8 | NP
KM373963.1 Influenza A virus A/mallard/Idaho/ND0002643/2013 H4N6 | NP
A/American green-winged teal/Texas/AH0040565/2016 H7N3 | NP
A/green-winged teal/California/96/2012 H1N1 | NP
A/northern pintail/Texas/A00466052/2009
A/blue-winged teal/Guatemala/CIP049H113-76/2013 H14N4 | NP
A/mallard/Maryland/12OS3662/2013 H11N9 | NP
A/American green-winged teal/Mississippi/300/2010
A/northern shoveler/Washington/44249-749/2006 H7N3 | NP
A/mallard/Alberta/152/2006 H1N3 | NP
A/white front goose/Alaska/477003/2007 H5N2 | NP
A/mallard/Minnesota/AI09-3228/2009 H3N1| NP
A/wild bird/Chile/1805/2008 H5N9 | NP
A/mallard/Washington/44338-195/2007 H12N5 | NP
A/Northern Pintail/Arizona/D1800538/2018 H12N2 | NP
A/American wigeon/California/LDC523/2016 H6N1 | NP
A/Bean Goose/South Korea/KNU18-6/2018 H6N5 | NP
A/American wigeon/California/HS007A/2015 H6N5 | NP
A/mallard/Interior Alaska/11BM03577/2011 H7N3 | NP
A/mallard/Alberta/793/2015 H3N8 | NP
A/bufflehead/Alaska/512/2014 H4N8 | NP
A/Cinnamon Teal/California/D1714969/2017 H6N2 | NP
A/northern pintail/Interior Alaska/9BM8109R0/2009 H8N4 | NP
A/American green winged teal/Mississippi/A00468514/2009 H7N7 | NP
A/mallard/Illinois/08OS2711/2008 H10N7 | NP
A/mallard/Wisconsin/11OS4493/2011 H1N3 | NP
A/northern pintail/Interior Alaska/9BM11409R0/2009 H12N5 | NP
A/mallard/Interior Alaska/9BM2705R0/2009 | NP
A/pintail/Alberta/69/2005 H1N1 | NP
A/mallard/Maryland/07OS742/2007 H1N8
A/blue-winged teal/Louisiana/AI09-5234/2009 H11N2 | NP
A/American green-winged teal/Mississippi/11OS256/2011 H1N1 | NP
A/mallard/Nevada/450206-4/2006 H5N2 | NP
A/green-winged teal/Alaska/239/2013 H3N8 | NP
A/blue-winged teal/Guatemala/CIP049H109-15/2012 H3N2| NP
A/duck/Fukui/181015/2015 H12N5 | NP
A/wild bird/Korea/8G39/2005 H9N2 | NP
A/aquatic bird/Korea/w222/2007 H5N2 | NP
A/ruddy turnstone/New Jersey/AI13-2285/2013 H10N8 | NP
A/duck/Hokkaido/W144/2017 H3N2 | NP
A/duck/Hokkaido/W151/2017 H3N2 | NP
A/duck/Aichi/231001/2014 H4N6 | NP
A/wild bird/Korea/A10/2012 H1N8 | NP
A/duck/Japan/11OG1038/2011 H5N2 | NP
A/duck/Chiba/121211/2012 H4N6 | NP
A/duck/Wuhan/WHYF05/2014 H9N2 | NP
A/baikal teal/Xianghai/426/2011 H5N2 | NP
A/aquatic bird/Korea/W69/2005H6N5 | NP
A/duck/Hokkaido/13/2015 H3N8 | NP
A/wild waterfowl/Korea/M54-F-1/2010 H6N2 | NP
A/aquatic bird/Korea/CN5/2009 H6N5 | NP
A/aquatic bird/Korea/CN5/2009H6N5 | NP
A/duck/Hunan/HN2| NP101/2013 H1N3 | NP
A/blue-winged teal/Guatemala/CIP049-S H117-143/2013 H14N3 | NP
A/duck/Korea/HP69/2007 H3N6 | NP
A/mandarin duck/Korea/K14-363-1/2014 H5N8 | NP
A/duck/Bangladesh/23858/2014 H5N1 | NP
A/chicken/Korea/Iksan/2010 H5N1 | NP
A/crane/Kagoshima/KU-34/2016 H5N6 | NP
A/duck/Bangladesh/1727/2010 H11N3 | NP
A/environment/Bangladesh/907/2009 H9N2 | NP
A/canine/Korea/GCVP01/2007 H3N2 | NP
A/aquatic bird/Korea/w217/2007 H5N2 | NP
A/Duck/Hunan/S3047/2009 H6N2 | NP
A/chicken/Korea/162/2006 H9N2 | NP
A/duck/Jiangxi/33757/2013 H10N3 | NP
A/Duck/Jiangsu/4/2018H7N4 | NP
A/common teal/Hong Kong/MPM1740/2011 H7N7 | NP
A/duck/Bangladesh/30884/2016 H5N1 | NP
A/wild bird/Eastern China/1759/2017 H5N3 | NP
A/common teal/Korea/W550/2016 H5N8 | NP
A/White-fronted Goose/South Korea/KNU18-119/2018 H7N7 | NP
A/Northern Goshawk/Tokyo/1301B003/2018 H5N6 | NP
A/duck/Korea/H56/2017 H5N6 | NP
A/duck/Chiba/6/2009 H4N6 | NP
A/EN/Hunan/37939/2015 H3N8 | NP
A/goose/Zhejiang/1120083/2014 H1N2 | NP
A/duck/Jiangxi/6450/2013 H10N8 | NP
A/spot-billed duck/Korea/447/11 H7N9 | NP
0.020
NAm
EA

e. NP

NAm

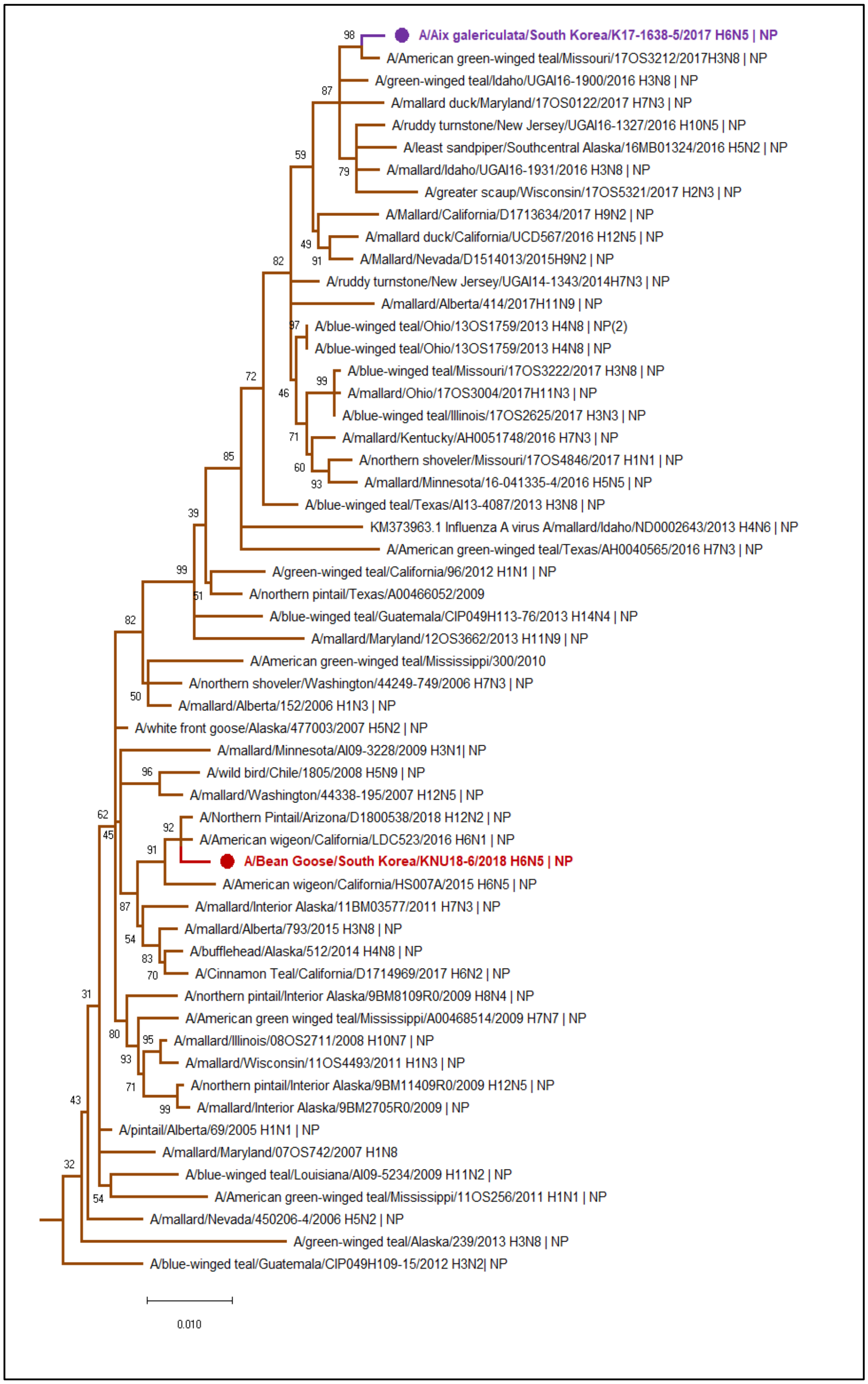
A/Aix galericulata/South Korea/K17-1638-5/2017 H6N5 | NP
A/American green-winged teal/Missouri/17OS3212/2017H3N8 | NP
A/green-winged teal/Idaho/UGAI16-1900/2016 H3N8 | NP
A/mallard duck/Maryland/17OS0122/2017 H7N3 | NP
A/ruddy turnstone/New Jersey/UGAI16-1327/2016 H10N5 | NP
A/least sandpiper/Southcentral Alaska/16MB01324/2016 H5N2 | NP
A/mallard/Idaho/UGAI16-1931/2016 H3N8 | NP
A/greater scaup/Wisconsin/17OS5321/2017 H2N3 | NP
A/Mallard/California/D1713634/2017 H9N2 | NP
A/mallard duck/California/UCD567/2016 H12N5 | NP
A/Mallard/Nevada/D1514013/2015H9N2 | NP
A/ruddy turnstone/New Jersey/UGAI14-1343/2014H7N3 | NP
A/mallard/Alberta/414/2017H11N9 | NP
A/blue-winged teal/Ohio/13OS1759/2013 H4N8 | NP(2)
A/blue-winged teal/Ohio/13OS1759/2013 H4N8 | NP
A/blue-winged teal/Missouri/17OS3222/2017 H3N8 | NP
A/mallard/Ohio/17OS3004/2017H11N3 | NP
A/blue-winged teal/Illinois/17OS2625/2017 H3N3 | NP
A/mallard/Kentucky/AH0051748/2016 H7N3 | NP
A/northern shoveler/Missouri/17OS4846/2017 H1N1 | NP
A/mallard/Minnesota/16-041335-4/2016 H5N5 | NP
A/blue-winged teal/Texas/AI13-4087/2013 H3N8 | NP
KM373963.1 Influenza A virus A/mallard/Idaho/ND0002643/2013 H4N6 | NP
A/American green-winged teal/Texas/AH0040565/2016 H7N3 | NP
A/green-winged teal/California/96/2012 H1N1 | NP
A/northern pintail/Texas/A00466052/2009
A/blue-winged teal/Guatemala/CIP049H113-76/2013 H14N4 | NP
A/mallard/Maryland/12OS3662/2013 H11N9 | NP
A/American green-winged teal/Mississippi/300/2010
A/northern shoveler/Washington/44249-749/2006 H7N3 | NP
A/mallard/Alberta/152/2006 H1N3 | NP
A/white front goose/Alaska/477003/2007 H5N2 | NP
A/mallard/Minnesota/AI09-3228/2009 H3N1| NP
A/wild bird/Chile/1805/2008 H5N9 | NP
A/mallard/Washington/44338-195/2007 H12N5 | NP
A/Northern Pintail/Arizona/D1800538/2018 H12N2 | NP
A/American wigeon/California/LDC523/2016 H6N1 | NP
A/Bean Goose/South Korea/KNU18-6/2018 H6N5 | NP
A/American wigeon/California/HS007A/2015 H6N5 | NP
A/mallard/Interior Alaska/11BM03577/2011 H7N3 | NP
A/mallard/Alberta/793/2015 H3N8 | NP
A/bufflehead/Alaska/512/2014 H4N8 | NP
A/Cinnamon Teal/California/D1714969/2017 H6N2 | NP
A/northern pintail/Interior Alaska/9BM8109R0/2009 H8N4 | NP
A/American green winged teal/Mississippi/A00468514/2009 H7N7 | NP
A/mallard/Illinois/08OS2711/2008 H10N7 | NP
A/mallard/Wisconsin/11OS4493/2011 H1N3 | NP
A/northern pintail/Interior Alaska/9BM11409R0/2009 H12N5 | NP
A/mallard/Interior Alaska/9BM2705R0/2009 | NP
A/pintail/Alberta/69/2005 H1N1 | NP
A/mallard/Maryland/07OS742/2007 H1N8
A/blue-winged teal/Louisiana/AI09-5234/2009 H11N2 | NP
A/American green-winged teal/Mississippi/11OS256/2011 H1N1 | NP
A/mallard/Nevada/450206-4/2006 H5N2 | NP
A/green-winged teal/Alaska/239/2013 H3N8 | NP
A/blue-winged teal/Guatemala/CIP049H109-15/2012 H3N2| NP
0.010

F. NA

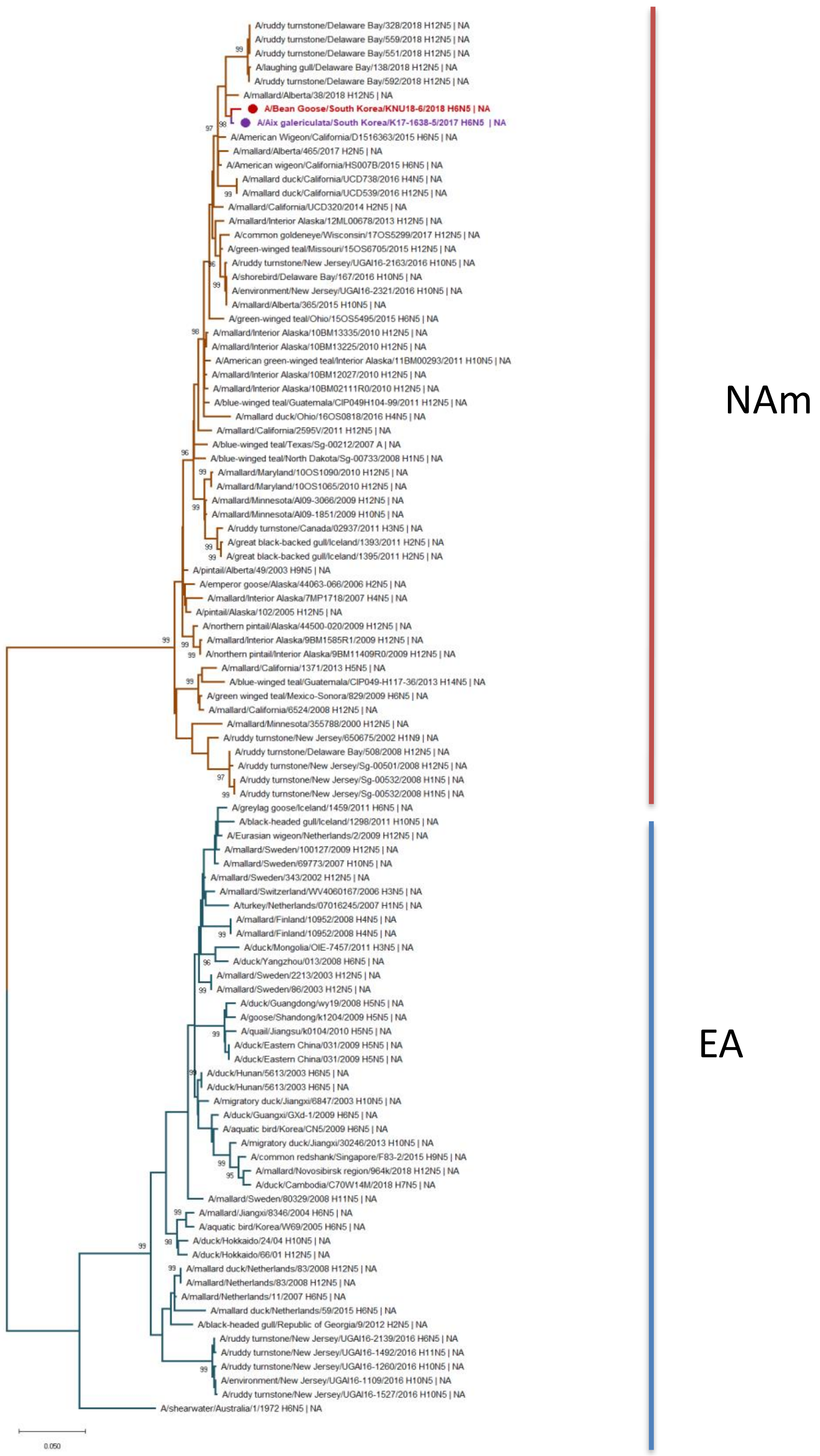

A/ruddy turnstone/Delaware Bay/328/2018 H12N5 | NA
A/ruddy turnstone/Delaware Bay/559/2018 H12N5 | NA
A/ruddy turnstone/Delaware Bay/551/2018 H12N5 | NA
A/laughing gull/Delaware Bay/138/2018 H12N5 | NA
A/ruddy turnstone/Delaware Bay/592/2018 H12N5 | NA
A/mallard/Alberta/38/2018 H12N5 | NA
A/Bean Goose/South Korea/KNU18-6/2018 H6N5 | NA
A/Aix galericulata/South Korea/K17-1638-5/2017 H6N5 | NA
A/American Wigeon/California/D1516363/2015 H6N5 | NA
A/mallard/Alberta/465/2017 H2N5 | NA
A/American wigeon/California/HS007B/2015 H6N5 | NA
A/mallard duck/California/UCD738/2016 H4N5 | NA
A/mallard duck/California/UCD539/2016 H12N5 | NA
A/mallard/California/UCD320/2014 H2N5 | NA
A/mallard/Interior Alaska/12ML00678/2013 H12N5 | NA
A/common goldeneye/Wisconsin/17OS5299/2017 H12N5 | NA
A/green-winged teal/Missouri/15OS6705/2015 H12N5 | NA
A/ruddy turnstone/New Jersey/UGAI16-2163/2016 H10N5 | NA
A/shorebird/Delaware Bay/167/2016 H10N5 | NA
A/environment/New Jersey/UGAI16-2321/2016 H10N5 | NA
A/mallard/Alberta/365/2015 H10N5 | NA
A/green-winged teal/Ohio/15OS5495/2015 H6N5 | NA
A/mallard/Interior Alaska/10BM13335/2010 H12N5 | NA
A/mallard/Interior Alaska/10BM13225/2010 H12N5 | NA
A/American green-winged teal/Interior Alaska/11BM00293/2011 H10N5 | NA
A/mallard/Interior Alaska/10BM12027/2010 H12N5 | NA
A/mallard/Interior Alaska/10BM02111R0/2010 H12N5 | NA
A/blue-winged teal/Guatemala/CIP049H104-99/2011 H12N5 | NA
A/mallard duck/Ohio/16OS0818/2016 H4N5 | NA
A/mallard/California/2595V/2011 H12N5 | NA
A/blue-winged teal/Texas/Sg-00212/2007 A | NA
A/blue-winged teal/North Dakota/Sg-00733/2008 H1N5 | NA
A/mallard/Maryland/10OS1090/2010 H12N5 | NA
A/mallard/Maryland/10OS1065/2010 H12N5 | NA
A/mallard/Minnesota/AI09-3066/2009 H12N5 | NA
A/mallard/Minnesota/AI09-1851/2009 H10N5 | NA
A/ruddy turnstone/Canada/02937/2011 H3N5 | NA
A/great black-backed gull/Iceland/1393/2011 H2N5 | NA
A/great black-backed gull/Iceland/1395/2011 H2N5 | NA
A/pintail/Alberta/49/2003 H9N5 | NA
A/emperor goose/Alaska/44063-066/2006 H2N5 | NA
A/mallard/Interior Alaska/7MP1718/2007 H4N5 | NA
A/pintail/Alaska/102/2005 H12N5 | NA
A/northern pintail/Alaska/44500-020/2009 H12N5 | NA
A/mallard/Interior Alaska/9BM1585R1/2009 H12N5 | NA
A/northern pintail/Interior Alaska/9BM11409R0/2009 H12N5 | NA
A/mallard/California/1371/2013 H5N5 | NA
A/blue-winged teal/Guatemala/CIP049-H117-36/2013 H14N5 | NA
A/green winged teal/Mexico-Sonora/829/2009 H6N5 | NA
A/mallard/California/6524/2008 H12N5 | NA
A/mallard/Minnesota/355788/2000 H12N5 | NA
A/ruddy turnstone/New Jersey/650675/2002 H1N9 | NA
A/ruddy turnstone/Delaware Bay/508/2008 H12N5 | NA
A/ruddy turnstone/New Jersey/Sg-00501/2008 H12N5 | NA
A/ruddy turnstone/New Jersey/Sg-00532/2008 H1N5 | NA
A/ruddy turnstone/New Jersey/Sg-00532/2008 H1N5 | NA
A/greylag goose/Iceland/1459/2011 H6N5 | NA
A/black-headed gull/Iceland/1298/2011 H10N5 | NA
A/Eurasian wigeon/Netherlands/2/2009 H12N5 | NA
A/mallard/Sweden/100127/2009 H12N5 | NA
A/mallard/Sweden/69773/2007 H10N5 | NA
A/mallard/Sweden/343/2002 H12N5 | NA
A/mallard/Switzerland/WV4060167/2006 H3N5 | NA
A/turkey/Netherlands/07016245/2007 H1N5 | NA
A/mallard/Finland/10952/2008 H4N5 | NA
A/mallard/Finland/10952/2008 H4N5 | NA
A/duck/Mongolia/OIE-7457/2011 H3N5 | NA
A/duck/Yangzhou/013/2008 H6N5 | NA
A/mallard/Sweden/2213/2003 H12N5 | NA
A/mallard/Sweden/86/2003 H12N5 | NA
A/duck/Guangdong/wy19/2008 H5N5 | NA
A/goose/Shandong/k1204/2009 H5N5 | NA
A/quail/Jiangsu/k0104/2010 H5N5 | NA
A/duck/Eastern China/031/2009 H5N5 | NA
A/duck/Eastern China/031/2009 H5N5 | NA
A/duck/Hunan/5613/2003 H6N5 | NA
A/duck/Hunan/5613/2003 H6N5 | NA
A/migratory duck/Jiangxi/6847/2003 H10N5 | NA
A/duck/Guangxi/GXd-1/2009 H6N5 | NA
A/aquatic bird/Korea/CN5/2009 H6N5 | NA
A/migratory duck/Jiangxi/30246/2013 H10N5 | NA
A/common redshank/Singapore/F83-2/2015 H9N5 | NA
A/mallard/Novosibirsk region/964k/2018 H12N5 | NA
A/duck/Cambodia/C70W14M/2018 H7N5 | NA
A/mallard/Sweden/80329/2008 H11N5 | NA
A/mallard/Jiangxi/8346/2004 H6N5 | NA
A/aquatic bird/Korea/W69/2005 H6N5 | NA
A/duck/Hokkaido/24/04 H10N5 | NA
A/duck/Hokkaido/66/01 H12N5 | NA
A/mallard duck/Netherlands/83/2008 H12N5 | NA
A/mallard/Netherlands/83/2008 H12N5 | NA
A/mallard/Netherlands/11/2007 H6N5 | NA
A/mallard duck/Netherlands/59/2015 H6N5 | NA
A/black-headed gull/Republic of Georgia/9/2012 H2N5 | NA
A/ruddy turnstone/New Jersey/UGAI16-2139/2016 H6N5 | NA
A/ruddy turnstone/New Jersey/UGAI16-1492/2016 H11N5 | NA
A/ruddy turnstone/New Jersey/UGAI16-1260/2016 H10N5 | NA
A/environment/New Jersey/UGAI16-1109/2016 H10N5 | NA
A/ruddy turnstone/New Jersey/UGAI16-1527/2016 H10N5 | NA
A/shearwater/Australia/1/1972 H6N5 | NA
0.050
NAm
EA

f. NA
NAm

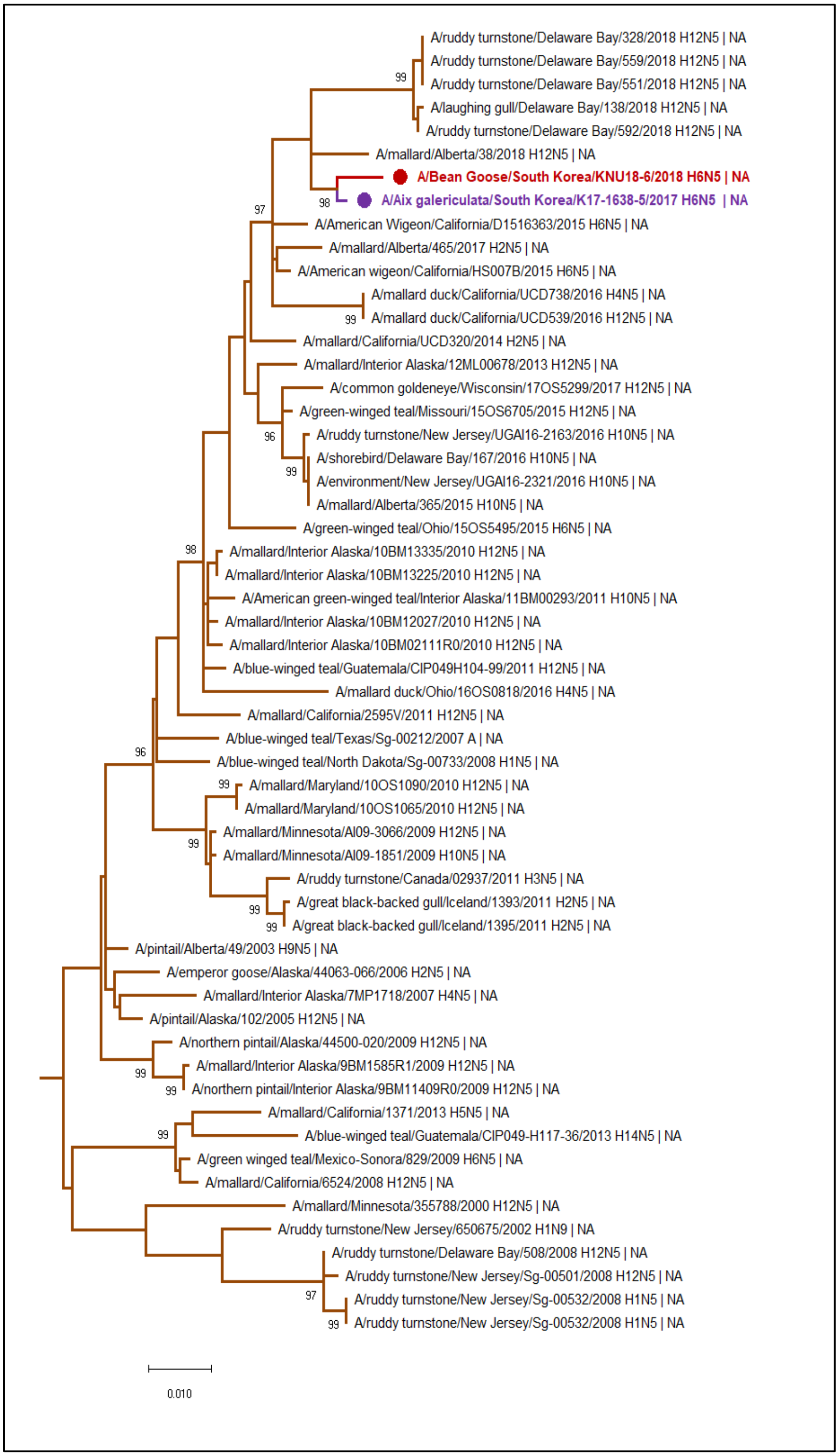

A/ruddy turnstone/Delaware Bay/328/2018 H12N5 | NA
A/ruddy turnstone/Delaware Bay/559/2018 H12N5 | NA
A/ruddy turnstone/Delaware Bay/551/2018 H12N5 | NA
A/laughing gull/Delaware Bay/138/2018 H12N5 | NA
A/ruddy turnstone/Delaware Bay/592/2018 H12N5 | NA
A/mallard/Alberta/38/2018 H12N5 | NA
A/Bean Goose/South Korea/KNU18-6/2018 H6N5 | NA
A/Aix galericulata/South Korea/K17-1638-5/2017 H6N5 | NA
A/American Wigeon/California/D1516363/2015 H6N5 | NA
A/mallard/Alberta/465/2017 H2N5 | NA
A/American wigeon/California/HS007B/2015 H6N5 | NA
A/mallard duck/California/UCD738/2016 H4N5 | NA
A/mallard duck/California/UCD539/2016 H12N5 | NA
A/mallard/California/UCD320/2014 H2N5 | NA
A/mallard/Interior Alaska/12ML00678/2013 H12N5 | NA
A/common goldeneye/Wisconsin/17OS5299/2017 H12N5 | NA
A/green-winged teal/Missouri/15OS6705/2015 H12N5 | NA
A/ruddy turnstone/New Jersey/UGAI16-2163/2016 H10N5 | NA
A/shorebird/Delaware Bay/167/2016 H10N5 | NA
A/environment/New Jersey/UGAI16-2321/2016 H10N5 | NA
A/mallard/Alberta/365/2015 H10N5 | NA
A/green-winged teal/Ohio/15OS5495/2015 H6N5 | NA
A/mallard/Interior Alaska/10BM13335/2010 H12N5 | NA
A/mallard/Interior Alaska/10BM13225/2010 H12N5 | NA
A/American green-winged teal/Interior Alaska/11BM00293/2011 H10N5 | NA
A/mallard/Interior Alaska/10BM12027/2010 H12N5 | NA
A/mallard/Interior Alaska/10BM02111R0/2010 H12N5 | NA
A/blue-winged teal/Guatemala/CIP049H104-99/2011 H12N5 | NA
A/mallard duck/Ohio/16OS0818/2016 H4N5 | NA
A/mallard/California/2595V/2011 H12N5 | NA
A/blue-winged teal/Texas/Sg-00212/2007 A | NA
A/blue-winged teal/North Dakota/Sg-00733/2008 H1N5 | NA
A/mallard/Maryland/10OS1090/2010 H12N5 | NA
A/mallard/Maryland/10OS1065/2010 H12N5 | NA
A/mallard/Minnesota/AI09-3066/2009 H12N5 | NA
A/mallard/Minnesota/AI09-1851/2009 H10N5 | NA
A/ruddy turnstone/Canada/02937/2011 H3N5 | NA
A/great black-backed gull/Iceland/1393/2011 H2N5 | NA
A/great black-backed gull/Iceland/1395/2011 H2N5 | NA
A/pintail/Alberta/49/2003 H9N5 | NA
A/emperor goose/Alaska/44063-066/2006 H2N5 | NA
A/mallard/Interior Alaska/7MP1718/2007 H4N5 | NA
A/pintail/Alaska/102/2005 H12N5 | NA
A/northern pintail/Alaska/44500-020/2009 H12N5 | NA
A/mallard/Interior Alaska/9BM1585R1/2009 H12N5 | NA
A/northern pintail/Interior Alaska/9BM11409R0/2009 H12N5 | NA
A/mallard/California/1371/2013 H5N5 | NA
A/blue-winged teal/Guatemala/CIP049-H117-36/2013 H14N5 | NA
A/green winged teal/Mexico-Sonora/829/2009 H6N5 | NA
A/mallard/California/6524/2008 H12N5 | NA
A/mallard/Minnesota/355788/2000 H12N5 | NA
A/ruddy turnstone/New Jersey/650675/2002 H1N9 | NA
A/ruddy turnstone/Delaware Bay/508/2008 H12N5 | NA
A/ruddy turnstone/New Jersey/Sg-00501/2008 H12N5 | NA
A/ruddy turnstone/New Jersey/Sg-00532/2008 H1N5 | NA
A/ruddy turnstone/New Jersey/Sg-00532/2008 H1N5 | NA
0.010

G. MP

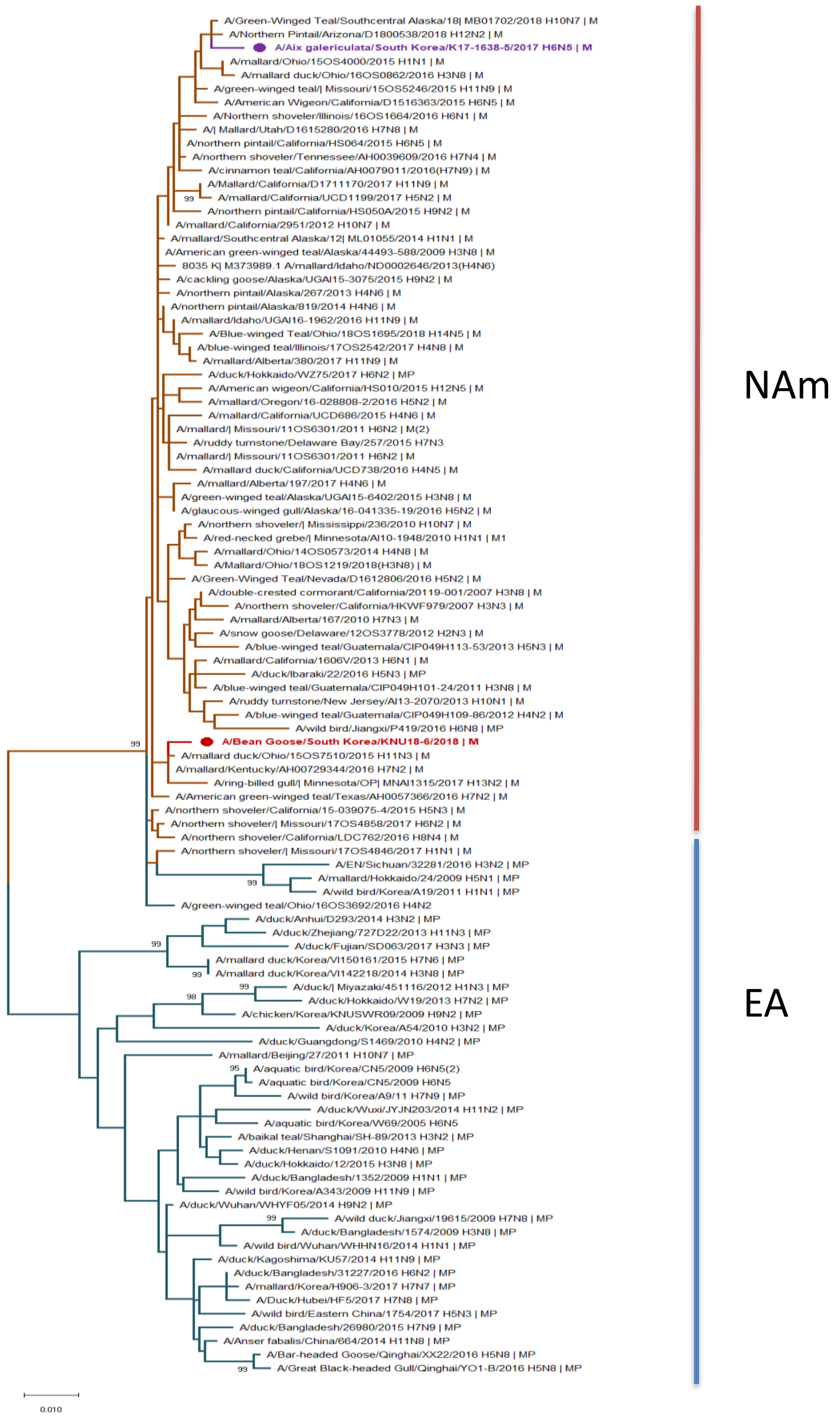

A/Green-Winged Teal/Southcentral Alaska/18| MB01702/2018 H10N7 | M
A/Northern Pintail/Arizona/D1800538/2018 H12N2 | M
A/Aix galericulata/South Korea/K17-1638-5/2017 H6N5 | M
A/mallard/Ohio/15OS4000/2015 H1N1 | M
A/mallard duck/Ohio/16OS0862/2016 H3N8 | M
A/green-winged teal/| Missouri/15OS5246/2015 H11N9 | M
A/American Wigeon/California/D1516363/2015 H6N5 | M
A/Northern shoveler/Illinois/16OS1664/2016 H6N1 | M
A/| Mallard/Utah/D1615280/2016 H7N8 | M
A/northern pintail/California/HS064/2015 H6N5 | M
A/northern shoveler/Tennessee/AH0039609/2016 H7N4 | M
A/cinnamon teal/California/AH0079011/2016(H7N9) | M
A/Mallard/California/D1711170/2017 H11N9 | M
99
A/mallard/California/UCD1199/2017 H5N2 | M
A/northern pintail/California/HS050A/2015 H9N2 | M
A/mallard/California/2951/2012 H10N7 | M
A/mallard/Southcentral Alaska/12| ML01055/2014 H1N1 | M
A/American green-winged teal/Alaska/44493-588/2009 H3N8 | M
8035 K| M373989.1 A/mallard/Idaho/ND0002646/2013(H4N6)
A/cackling goose/Alaska/UGAI15-3075/2015 H9N2 | M
A/northern pintail/Alaska/267/2013 H4N6 | M
A/northern pintail/Alaska/819/2014 H4N6 | M
A/mallard/Idaho/UGAI16-1962/2016 H11N9 | M
A/Blue-winged Teal/Ohio/18OS1695/2018 H14N5 | M
A/blue-winged teal/Illinois/17OS2542/2017 H4N8 | M
A/mallard/Alberta/380/2017 H11N9 | M
A/duck/Hokkaido/WZ75/2017 H6N2 | MP
A/American wigeon/California/HS010/2015 H12N5 | M
A/mallard/Oregon/16-028808-2/2016 H5N2 | M
A/mallard/California/UCD686/2015 H4N6 | M
A/mallard/| Missouri/11OS6301/2011 H6N2 | M(2)
A/ruddy turnstone/Delaware Bay/257/2015 H7N3
A/mallard/| Missouri/11OS6301/2011 H6N2 | M
A/mallard duck/California/UCD738/2016 H4N5 | M
A/mallard/Alberta/197/2017 H4N6 | M
A/green-winged teal/Alaska/UGAI15-6402/2015 H3N8 | M
A/glaucous-winged gull/Alaska/16-041335-19/2016 H5N2 | M
A/northern shoveler/| Mississippi/236/2010 H10N7 | M
A/red-necked grebe/| Minnesota/AI10-1948/2010 H1N1 | M1
A/mallard/Ohio/14OS0573/2014 H4N8 | M
A/Mallard/Ohio/18OS1219/2018(H3N8) | M
A/Green-Winged Teal/Nevada/D1612806/2016 H5N2 | M
A/double-crested cormorant/California/20119-001/2007 H3N8 | M
A/northern shoveler/California/HKWF979/2007 H3N3 | M
A/mallard/Alberta/167/2010 H7N3 | M
A/snow goose/Delaware/12OS3778/2012 H2N3 | M
A/blue-winged teal/Guatemala/CIP049H113-53/2013 H5N3 | M
A/mallard/California/1606V/2013 H6N1 | M
A/duck/Ibaraki/22/2016 H5N3 | MP
A/blue-winged teal/Guatemala/CIP049H101-24/2011 H3N8 | M
A/ruddy turnstone/New Jersey/AI13-2070/2013 H10N1 | M
A/blue-winged teal/Guatemala/CIP049H109-86/2012 H4N2 | M
A/wild bird/Jiangxi/P419/2016 H6N8 | MP
99
A/Bean Goose/South Korea/KNU18-6/2018 | M
A/mallard duck/Ohio/15OS7510/2015 H11N3 | M
A/mallard/Kentucky/AH00729344/2016 H7N2 | M
A/ring-billed gull/| Minnesota/OP| MNAI1315/2017 H13N2 | M
A/American green-winged teal/Texas/AH0057366/2016 H7N2 | M
A/northern shoveler/California/15-039075-4/2015 H5N3 | M
A/northern shoveler/| Missouri/17OS4858/2017 H6N2 | M
A/northern shoveler/California/LDC762/2016 H8N4 | M
A/northern shoveler/| Missouri/17OS4846/2017 H1N1 | M
A/EN/Sichuan/32281/2016 H3N2 | MP
A/mallard/Hokkaido/24/2009 H5N1 | MP
99
A/wild bird/Korea/A19/2011 H1N1 | MP
A/green-winged teal/Ohio/16OS3692/2016 H4N2
A/duck/Anhui/D293/2014 H3N2 | MP
A/duck/Zhejiang/727D22/2013 H11N3 | MP
99
A/duck/Fujian/SD063/2017 H3N3 | MP
A/mallard duck/Korea/VI150161/2015 H7N6 | MP
99
A/mallard duck/Korea/VI142218/2014 H3N8 | MP
99
A/duck/| Miyazaki/451116/2012 H1N3 | MP
98
A/duck/Hokkaido/W19/2013 H7N2 | MP
A/chicken/Korea/KNUSWR09/2009 H9N2 | MP
A/duck/Korea/A54/2010 H3N2 | MP
A/duck/Guangdong/S1469/2010 H4N2 | MP
A/mallard/Beijing/27/2011 H10N7 | MP
95
A/aquatic bird/Korea/CN5/2009 H6N5(2)
A/aquatic bird/Korea/CN5/2009 H6N5
A/wild bird/Korea/A9/11 H7N9 | MP
A/duck/Wuxi/JYJN203/2014 H11N2 | MP
A/aquatic bird/Korea/W69/2005 H6N5
A/baikal teal/Shanghai/SH-89/2013 H3N2 | MP
A/duck/Henan/S1091/2010 H4N6 | MP
A/duck/Hokkaido/12/2015 H3N8 | MP
A/duck/Bangladesh/1352/2009 H1N1 | MP
A/wild bird/Korea/A343/2009 H11N9 | MP
A/duck/Wuhan/WHYF05/2014 H9N2 | MP
99
A/wild duck/Jiangxi/19615/2009 H7N8 | MP
A/duck/Bangladesh/1574/2009 H3N8 | MP
A/wild bird/Wuhan/WHHN16/2014 H1N1 | MP
A/duck/Kagoshima/KU57/2014 H11N9 | MP
A/duck/Bangladesh/31227/2016 H6N2 | MP
A/mallard/Korea/H906-3/2017 H7N7 | MP
A/Duck/Hubei/HF5/2017 H7N8 | MP
A/wild bird/Eastern China/1754/2017 H5N3 | MP
A/duck/Bangladesh/26980/2015 H7N9 | MP
A/Anser fabalis/China/664/2014 H11N8 | MP
A/Bar-headed Goose/Qinghai/XX22/2016 H5N8 | MP
99
A/Great Black-headed Gull/Qinghai/YO1-B/2016 H5N8 | MP
NAm
EA
0.010

g. MP
NAm

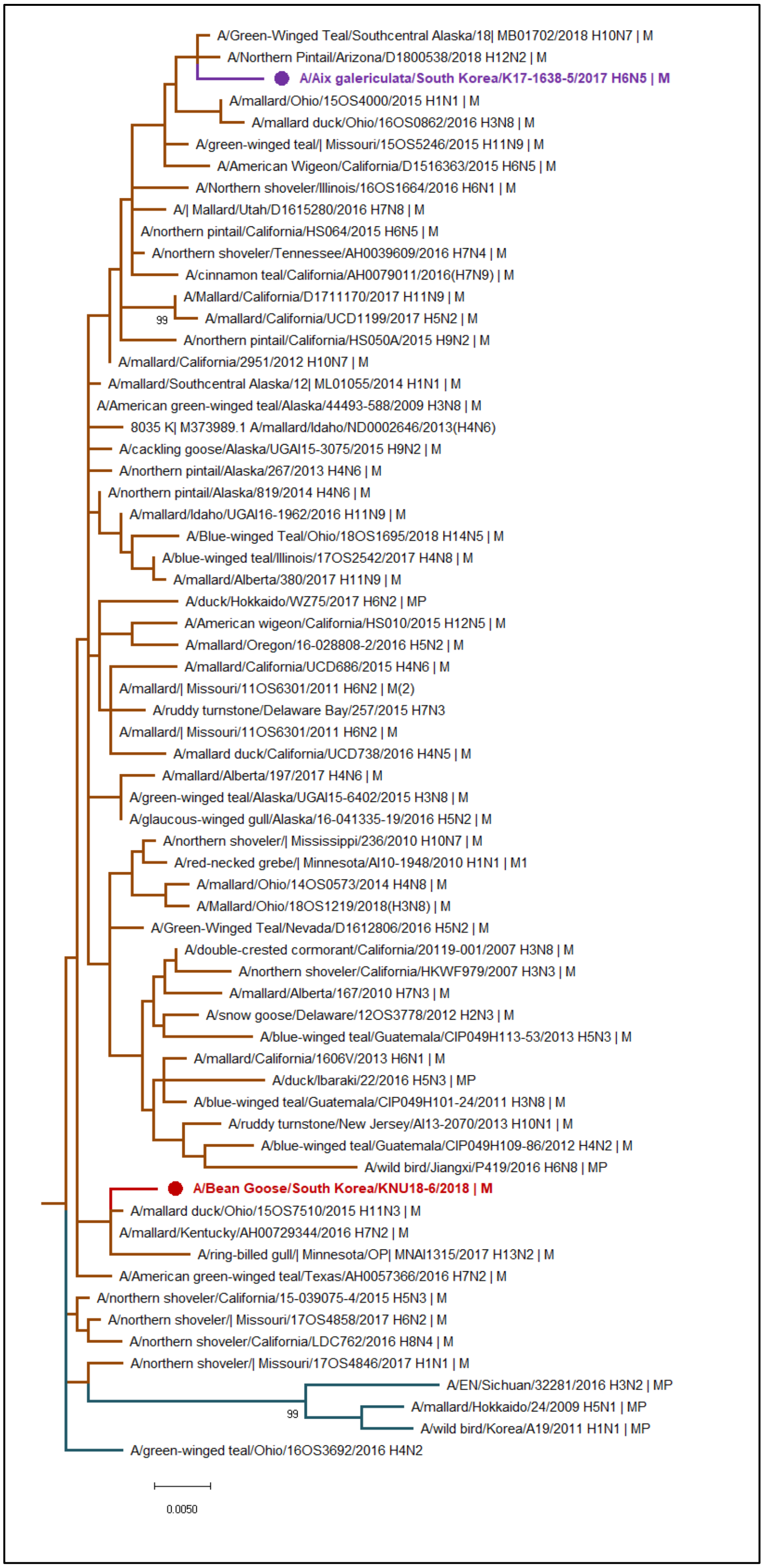
A/Green-Winged Teal/Southcentral Alaska/18| MB01702/2018 H10N7 | M
A/Northern Pintail/Arizona/D1800538/2018 H12N2 | M
A/Aix galericulata/South Korea/K17-1638-5/2017 H6N5 | M
A/mallard/Ohio/15OS4000/2015 H1N1 | M
A/mallard duck/Ohio/16OS0862/2016 H3N8 | M
A/green-winged teal/| Missouri/15OS5246/2015 H11N9 | M
A/American Wigeon/California/D1516363/2015 H6N5 | M
A/Northern shoveler/Illinois/16OS1664/2016 H6N1 | M
A/| Mallard/Utah/D1615280/2016 H7N8 | M
A/northern pintail/California/HS064/2015 H6N5 | M
A/northern shoveler/Tennessee/AH0039609/2016 H7N4 | M
A/cinnamon teal/California/AH0079011/2016(H7N9) | M
A/Mallard/California/D1711170/2017 H11N9 | M
99
A/mallard/California/UCD1199/2017 H5N2 | M
A/northern pintail/California/HS050A/2015 H9N2 | M
A/mallard/California/2951/2012 H10N7 | M
A/mallard/Southcentral Alaska/12| ML01055/2014 H1N1 | M
A/American green-winged teal/Alaska/44493-588/2009 H3N8 | M
8035 K| M373989.1 A/mallard/Idaho/ND0002646/2013(H4N6)
A/cackling goose/Alaska/UGAI15-3075/2015 H9N2 | M
A/northern pintail/Alaska/267/2013 H4N6 | M
A/northern pintail/Alaska/819/2014 H4N6 | M
A/mallard/Idaho/UGAI16-1962/2016 H11N9 | M
A/Blue-winged Teal/Ohio/18OS1695/2018 H14N5 | M
A/blue-winged teal/Illinois/17OS2542/2017 H4N8 | M
A/mallard/Alberta/380/2017 H11N9 | M
A/duck/Hokkaido/WZ75/2017 H6N2 | MP
A/American wigeon/California/HS010/2015 H12N5 | M
A/mallard/Oregon/16-028808-2/2016 H5N2 | M
A/mallard/California/UCD686/2015 H4N6 | M
A/mallard/| Missouri/11OS6301/2011 H6N2 | M(2)
A/ruddy turnstone/Delaware Bay/257/2015 H7N3
A/mallard/| Missouri/11OS6301/2011 H6N2 | M
A/mallard duck/California/UCD738/2016 H4N5 | M
A/mallard/Alberta/197/2017 H4N6 | M
A/green-winged teal/Alaska/UGAI15-6402/2015 H3N8 | M
A/glaucous-winged gull/Alaska/16-041335-19/2016 H5N2 | M
A/northern shoveler/| Mississippi/236/2010 H10N7 | M
A/red-necked grebe/| Minnesota/AI10-1948/2010 H1N1 | M1
A/mallard/Ohio/14OS0573/2014 H4N8 | M
A/Mallard/Ohio/18OS1219/2018(H3N8) | M
A/Green-Winged Teal/Nevada/D1612806/2016 H5N2 | M
A/double-crested cormorant/California/20119-001/2007 H3N8 | M
A/northern shoveler/California/HKWF979/2007 H3N3 | M
A/mallard/Alberta/167/2010 H7N3 | M
A/snow goose/Delaware/12OS3778/2012 H2N3 | M
A/blue-winged teal/Guatemala/CIP049H113-53/2013 H5N3 | M
A/mallard/California/1606V/2013 H6N1 | M
A/duck/Ibaraki/22/2016 H5N3 | MP
A/blue-winged teal/Guatemala/CIP049H101-24/2011 H3N8 | M
A/ruddy turnstone/New Jersey/AI13-2070/2013 H10N1 | M
A/blue-winged teal/Guatemala/CIP049H109-86/2012 H4N2 | M
A/wild bird/Jiangxi/P419/2016 H6N8 | MP
A/Bean Goose/South Korea/KNU18-6/2018 | M
A/mallard duck/Ohio/15OS7510/2015 H11N3 | M
A/mallard/Kentucky/AH00729344/2016 H7N2 | M
A/ring-billed gull/| Minnesota/OP| MNAI1315/2017 H13N2 | M
A/American green-winged teal/Texas/AH0057366/2016 H7N2 | M
A/northern shoveler/California/15-039075-4/2015 H5N3 | M
A/northern shoveler/| Missouri/17OS4858/2017 H6N2 | M
A/northern shoveler/California/LDC762/2016 H8N4 | M
A/northern shoveler/| Missouri/17OS4846/2017 H1N1 | M
A/EN/Sichuan/32281/2016 H3N2 | MP
A/mallard/Hokkaido/24/2009 H5N1 | MP
99
A/wild bird/Korea/A19/2011 H1N1 | MP
A/green-winged teal/Ohio/16OS3692/2016 H4N2
0.0050

H. NS

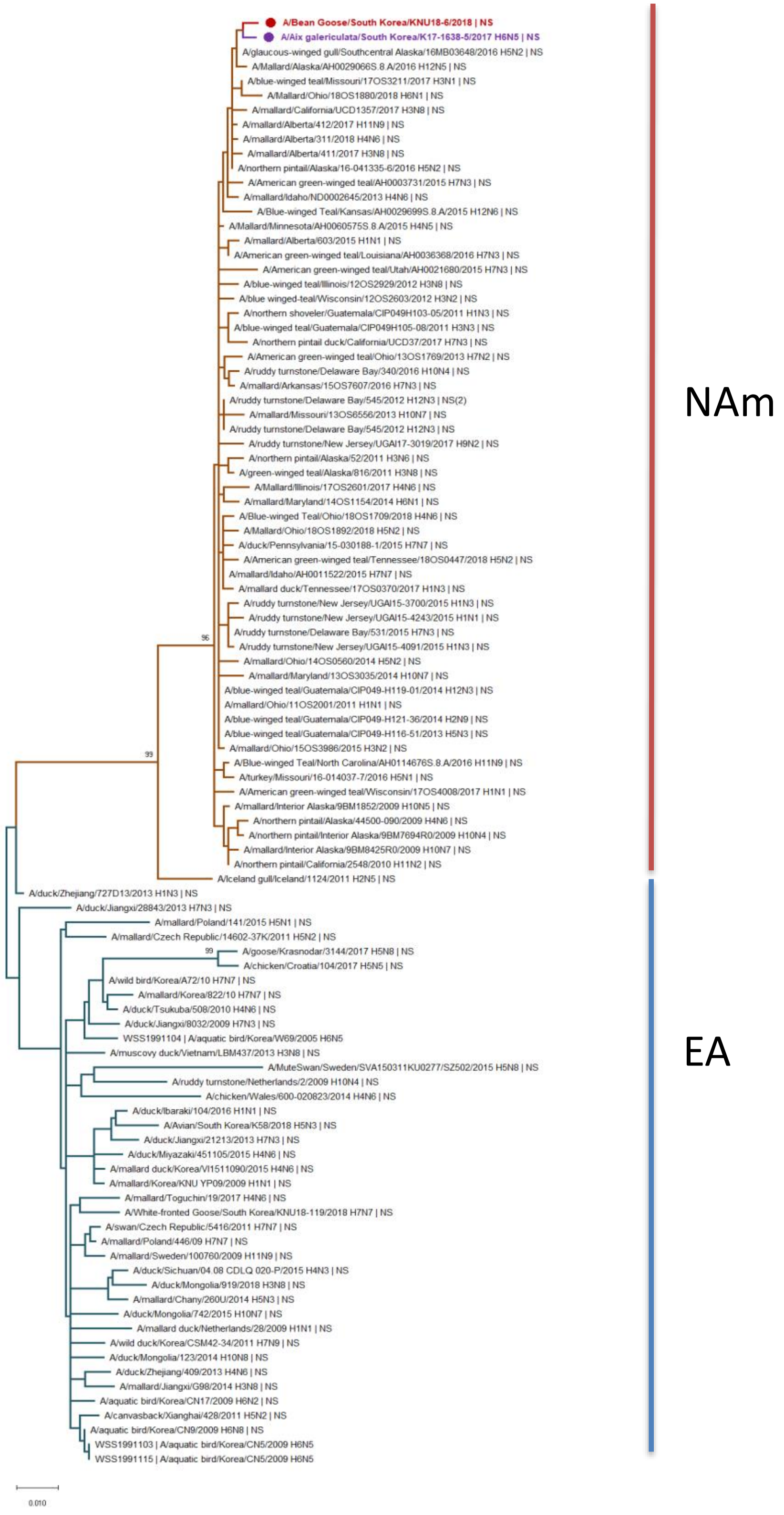
A/Bean Goose/South Korea/KNU18-6/2018 | NS
A/Aix galericulata/South Korea/K17-1638-5/2017 H6N5 | NS
A/glaucous-winged gull/Southcentral Alaska/16MB03648/2016 H5N2 | NS
A/Mallard/Alaska/AH0029066S.8.A/2016 H12N5 | NS
A/blue-winged teal/Missouri/17OS3211/2017 H3N1 | NS
A/Mallard/Ohio/18OS1880/2018 H6N1 | NS
A/mallard/California/UCD1357/2017 H3N8 | NS
A/mallard/Alberta/412/2017 H11N9 | NS
A/mallard/Alberta/311/2018 H4N6 | NS
A/mallard/Alberta/411/2017 H3N8 | NS
A/northern pintail/Alaska/16-041335-6/2016 H5N2 | NS
A/American green-winged teal/AH0003731/2015 H7N3 | NS
A/mallard/Idaho/ND0002645/2013 H4N6 | NS
A/Blue-winged Teal/Kansas/AH0029699S.8.A/2015 H12N6 | NS
A/Mallard/Minnesota/AH0060575S.8.A/2015 H4N5 | NS
A/mallard/Alberta/603/2015 H1N1 | NS
A/American green-winged teal/Louisiana/AH0036368/2016 H7N3 | NS
A/American green-winged teal/Utah/AH0021680/2015 H7N3 | NS
A/blue-winged teal/Illinois/12OS2929/2012 H3N8 | NS
A/blue winged-teal/Wisconsin/12OS2603/2012 H3N2 | NS
A/northern shoveler/Guatemala/CIP049H103-05/2011 H1N3 | NS
A/blue-winged teal/Guatemala/CIP049H105-08/2011 H3N3 | NS
A/northern pintail duck/California/UCD37/2017 H7N3 | NS
A/American green-winged teal/Ohio/13OS1769/2013 H7N2 | NS
A/ruddy turnstone/Delaware Bay/340/2016 H10N4 | NS
A/mallard/Arkansas/15OS7607/2016 H7N3 | NS
A/ruddy turnstone/Delaware Bay/545/2012 H12N3 | NS(2)
A/mallard/Missouri/13OS6556/2013 H10N7 | NS
A/ruddy turnstone/Delaware Bay/545/2012 H12N3 | NS
A/ruddy turnstone/New Jersey/UGAI17-3019/2017 H9N2 | NS
A/northern pintail/Alaska/52/2011 H3N6 | NS
A/green-winged teal/Alaska/816/2011 H3N8 | NS
A/Mallard/Illinois/17OS2601/2017 H4N6 | NS
A/mallard/Maryland/14OS1154/2014 H6N1 | NS
A/Blue-winged Teal/Ohio/18OS1709/2018 H4N6 | NS
A/Mallard/Ohio/18OS1892/2018 H5N2 | NS
A/duck/Pennsylvania/15-030188-1/2015 H7N7 | NS
A/American green-winged teal/Tennessee/18OS0447/2018 H5N2 | NS
A/mallard/Idaho/AH0011522/2015 H7N7 | NS
A/mallard duck/Tennessee/17OS0370/2017 H1N3 | NS
A/ruddy turnstone/New Jersey/UGAI15-3700/2015 H1N3 | NS
A/ruddy turnstone/New Jersey/UGAI15-4243/2015 H1N1 | NS
A/ruddy turnstone/Delaware Bay/531/2015 H7N3 | NS
A/ruddy turnstone/New Jersey/UGAI15-4091/2015 H1N3 | NS
96
A/mallard/Ohio/14OS0560/2014 H5N2 | NS
A/mallard/Maryland/13OS3035/2014 H10N7 | NS
A/blue-winged teal/Guatemala/CIP049-H119-01/2014 H12N3 | NS
A/mallard/Ohio/11OS2001/2011 H1N1 | NS
A/blue-winged teal/Guatemala/CIP049-H121-36/2014 H2N9 | NS
A/blue-winged teal/Guatemala/CIP049-H116-51/2013 H5N3 | NS
A/mallard/Ohio/15OS3986/2015 H3N2 | NS
99
A/Blue-winged Teal/North Carolina/AH0114676S.8.A/2016 H11N9 | NS
A/turkey/Missouri/16-014037-7/2016 H5N1 | NS
A/American green-winged teal/Wisconsin/17OS4008/2017 H1N1 | NS
A/mallard/Interior Alaska/9BM1852/2009 H10N5 | NS
A/northern pintail/Alaska/44500-090/2009 H4N6 | NS
A/northern pintail/Interior Alaska/9BM7694R0/2009 H10N4 | NS
A/mallard/Interior Alaska/9BM8425R0/2009 H10N7 | NS
A/northern pintail/California/2548/2010 H11N2 | NS
A/Iceland gull/Iceland/1124/2011 H2N5 | NS
A/duck/Zhejiang/727D13/2013 H1N3 | NS
A/duck/Jiangxi/28843/2013 H7N3 | NS
A/mallard/Poland/141/2015 H5N1 | NS
A/mallard/Czech Republic/14602-37K/2011 H5N2 | NS
99
A/goose/Krasnodar/3144/2017 H5N8 | NS
A/chicken/Croatia/104/2017 H5N5 | NS
A/wild bird/Korea/A72/10 H7N7 | NS
A/mallard/Korea/822/10 H7N7 | NS
A/duck/Tsukuba/508/2010 H4N6 | NS
A/duck/Jiangxi/8032/2009 H7N3 | NS
WSS1991104 | A/aquatic bird/Korea/W69/2005 H6N5
A/muscovy duck/Vietnam/LBM437/2013 H3N8 | NS
A/MuteSwan/Sweden/SVA150311KU0277/SZ502/2015 H5N8 | NS
A/ruddy turnstone/Netherlands/2/2009 H10N4 | NS
A/chicken/Wales/600-020823/2014 H4N6 | NS
A/duck/Ibaraki/104/2016 H1N1 | NS
A/Avian/South Korea/K58/2018 H5N3 | NS
A/duck/Jiangxi/21213/2013 H7N3 | NS
A/duck/Miyazaki/451105/2015 H4N6 | NS
A/mallard duck/Korea/VI1511090/2015 H4N6 | NS
A/mallard/Korea/KNU YP09/2009 H1N1 | NS
A/mallard/Toguchin/19/2017 H4N6 | NS
A/White-fronted Goose/South Korea/KNU18-119/2018 H7N7 | NS
A/swan/Czech Republic/5416/2011 H7N7 | NS
A/mallard/Poland/446/09 H7N7 | NS
A/mallard/Sweden/100760/2009 H11N9 | NS
A/duck/Sichuan/04.08 CDLQ 020-P/2015 H4N3 | NS
A/duck/Mongolia/919/2018 H3N8 | NS
A/mallard/Chany/260U/2014 H5N3 | NS
A/duck/Mongolia/742/2015 H10N7 | NS
A/mallard duck/Netherlands/28/2009 H1N1 | NS
A/wild duck/Korea/CSM42-34/2011 H7N9 | NS
A/duck/Mongolia/123/2014 H10N8 | NS
A/duck/Zhejiang/409/2013 H4N6 | NS
A/mallard/Jiangxi/G98/2014 H3N8 | NS
A/aquatic bird/Korea/CN17/2009 H6N2 | NS
A/canvasback/Xianghai/428/2011 H5N2 | NS
A/aquatic bird/Korea/CN9/2009 H6N8 | NS
WSS1991103 | A/aquatic bird/Korea/CN5/2009 H6N5
WSS1991115 | A/aquatic bird/Korea/CN5/2009 H6N5
0.010
NAm
EA

h. NS
NAm

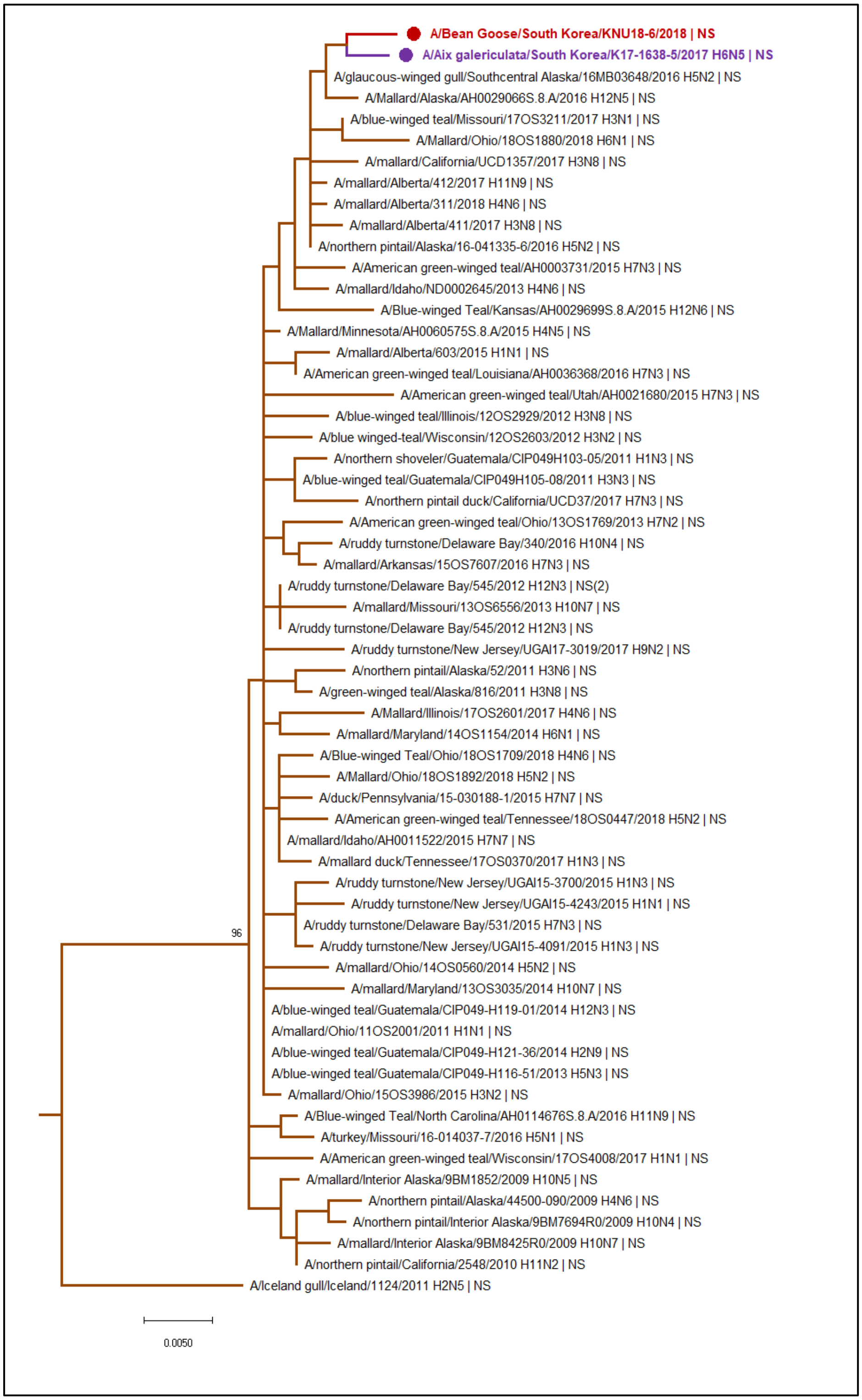
A/Bean Goose/South Korea/KNU18-6/2018 | NS
A/Aix galericulata/South Korea/K17-1638-5/2017 H6N5 | NS
A/glaucous-winged gull/Southcentral Alaska/16MB03648/2016 H5N2 | NS
A/Mallard/Alaska/AH0029066S.8.A/2016 H12N5 | NS
A/blue-winged teal/Missouri/17OS3211/2017 H3N1 | NS
A/Mallard/Ohio/18OS1880/2018 H6N1 | NS
A/mallard/California/UCD1357/2017 H3N8 | NS
A/mallard/Alberta/412/2017 H11N9 | NS
A/mallard/Alberta/311/2018 H4N6 | NS
A/mallard/Alberta/411/2017 H3N8 | NS
A/northern pintail/Alaska/16-041335-6/2016 H5N2 | NS
A/American green-winged teal/AH0003731/2015 H7N3 | NS
A/mallard/Idaho/ND0002645/2013 H4N6 | NS
A/Blue-winged Teal/Kansas/AH0029699S.8.A/2015 H12N6 | NS
A/Mallard/Minnesota/AH0060575S.8.A/2015 H4N5 | NS
A/mallard/Alberta/603/2015 H1N1 | NS
A/American green-winged teal/Louisiana/AH0036368/2016 H7N3 | NS
A/American green-winged teal/Utah/AH0021680/2015 H7N3 | NS
A/blue-winged teal/Illinois/12OS2929/2012 H3N8 | NS
A/blue winged-teal/Wisconsin/12OS2603/2012 H3N2 | NS
A/northern shoveler/Guatemala/CIP049H103-05/2011 H1N3 | NS
A/blue-winged teal/Guatemala/CIP049H105-08/2011 H3N3 | NS
A/northern pintail duck/California/UCD37/2017 H7N3 | NS
A/American green-winged teal/Ohio/13OS1769/2013 H7N2 | NS
A/ruddy turnstone/Delaware Bay/340/2016 H10N4 | NS
A/mallard/Arkansas/15OS7607/2016 H7N3 | NS
A/ruddy turnstone/Delaware Bay/545/2012 H12N3 | NS(2)
A/mallard/Missouri/13OS6556/2013 H10N7 | NS
A/ruddy turnstone/Delaware Bay/545/2012 H12N3 | NS
A/ruddy turnstone/New Jersey/UGAI17-3019/2017 H9N2 | NS
A/northern pintail/Alaska/52/2011 H3N6 | NS
A/green-winged teal/Alaska/816/2011 H3N8 | NS
A/Mallard/Illinois/17OS2601/2017 H4N6 | NS
A/mallard/Maryland/14OS1154/2014 H6N1 | NS
A/Blue-winged Teal/Ohio/18OS1709/2018 H4N6 | NS
A/Mallard/Ohio/18OS1892/2018 H5N2 | NS
A/duck/Pennsylvania/15-030188-1/2015 H7N7 | NS
A/American green-winged teal/Tennessee/18OS0447/2018 H5N2 | NS
A/mallard/Idaho/AH0011522/2015 H7N7 | NS
A/mallard duck/Tennessee/17OS0370/2017 H1N3 | NS
A/ruddy turnstone/New Jersey/UGAI15-3700/2015 H1N3 | NS
A/ruddy turnstone/New Jersey/UGAI15-4243/2015 H1N1 | NS
A/ruddy turnstone/Delaware Bay/531/2015 H7N3 | NS
A/ruddy turnstone/New Jersey/UGAI15-4091/2015 H1N3 | NS
96
A/mallard/Ohio/14OS0560/2014 H5N2 | NS
A/mallard/Maryland/13OS3035/2014 H10N7 | NS
A/blue-winged teal/Guatemala/CIP049-H119-01/2014 H12N3 | NS
A/mallard/Ohio/11OS2001/2011 H1N1 | NS
A/blue-winged teal/Guatemala/CIP049-H121-36/2014 H2N9 | NS
A/blue-winged teal/Guatemala/CIP049-H116-51/2013 H5N3 | NS
A/mallard/Ohio/15OS3986/2015 H3N2 | NS
A/Blue-winged Teal/North Carolina/AH0114676S.8.A/2016 H11N9 | NS
A/turkey/Missouri/16-014037-7/2016 H5N1 | NS
A/American green-winged teal/Wisconsin/17OS4008/2017 H1N1 | NS
A/mallard/Interior Alaska/9BM1852/2009 H10N5 | NS
A/northern pintail/Alaska/44500-090/2009 H4N6 | NS
A/northern pintail/Interior Alaska/9BM7694R0/2009 H10N4 | NS
A/mallard/Interior Alaska/9BM8425R0/2009 H10N7 | NS
A/northern pintail/California/2548/2010 H11N2 | NS
A/Iceland gull/Iceland/1124/2011 H2N5 | NS
0.0050

Figure S2. Phylogenetic tree of whole genome of the K6 avian influenza virus (AIV) compared to influenza sequences available in NCBI and GISAID. K6 was marked in red color and K17 was marked in purple color. Nam, North America lineage; EA, Eurasian lineage.

PB2

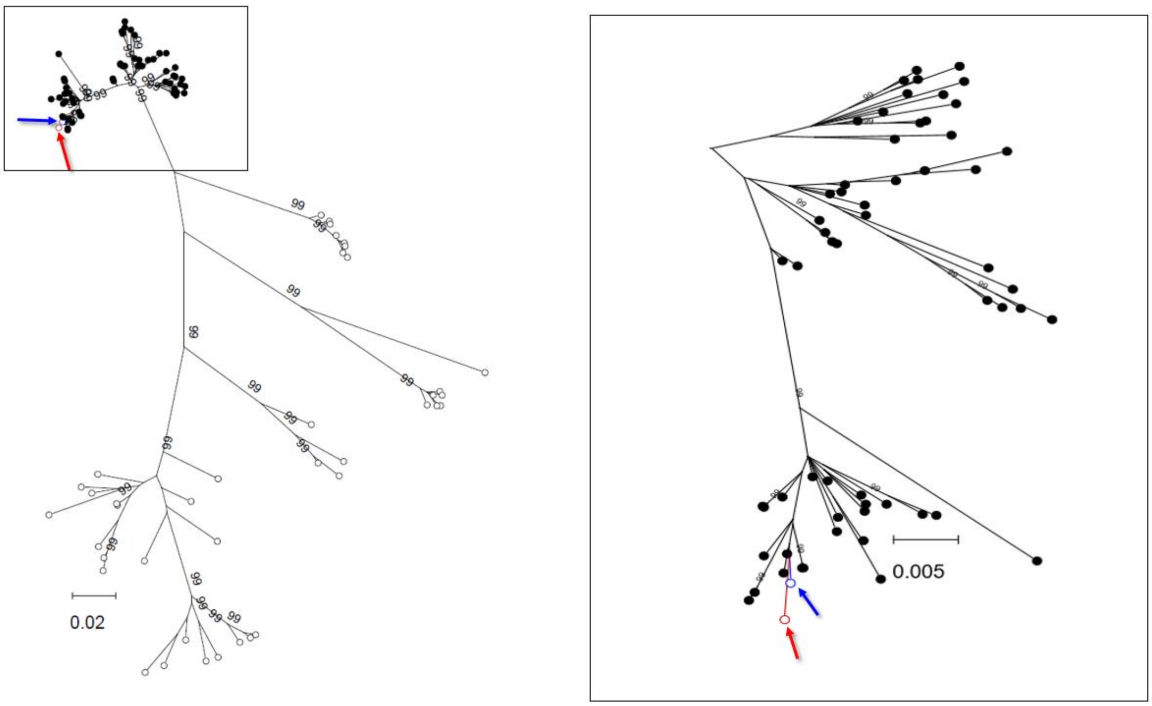

PB1

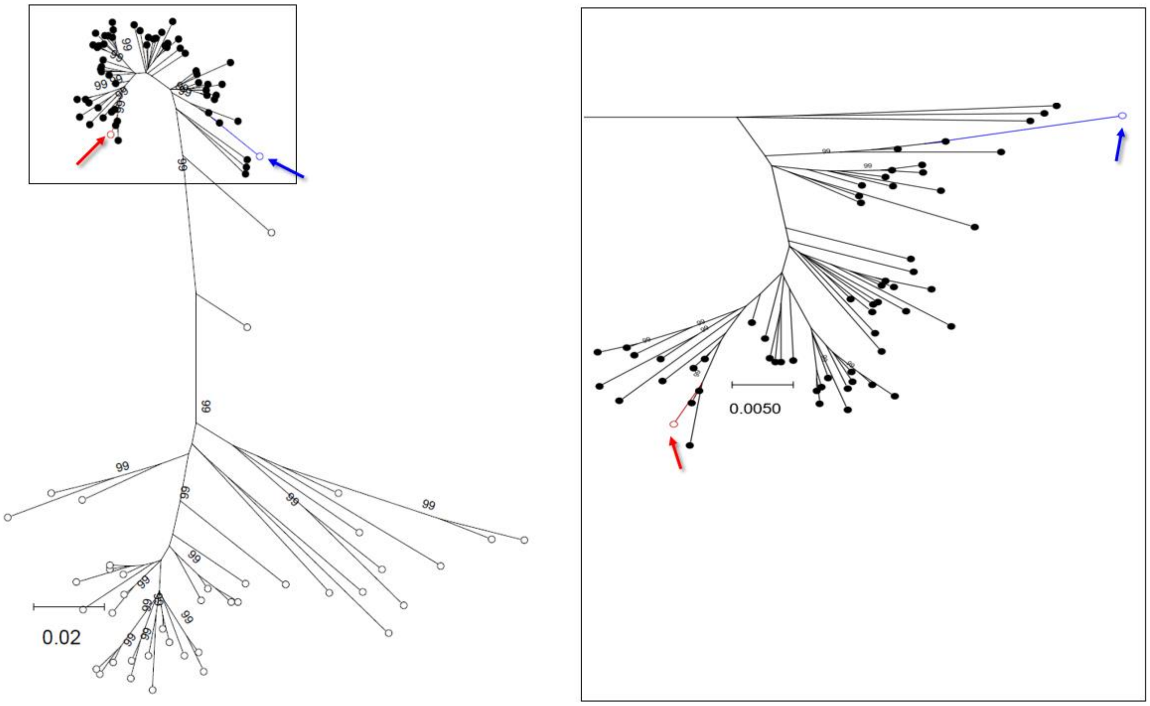

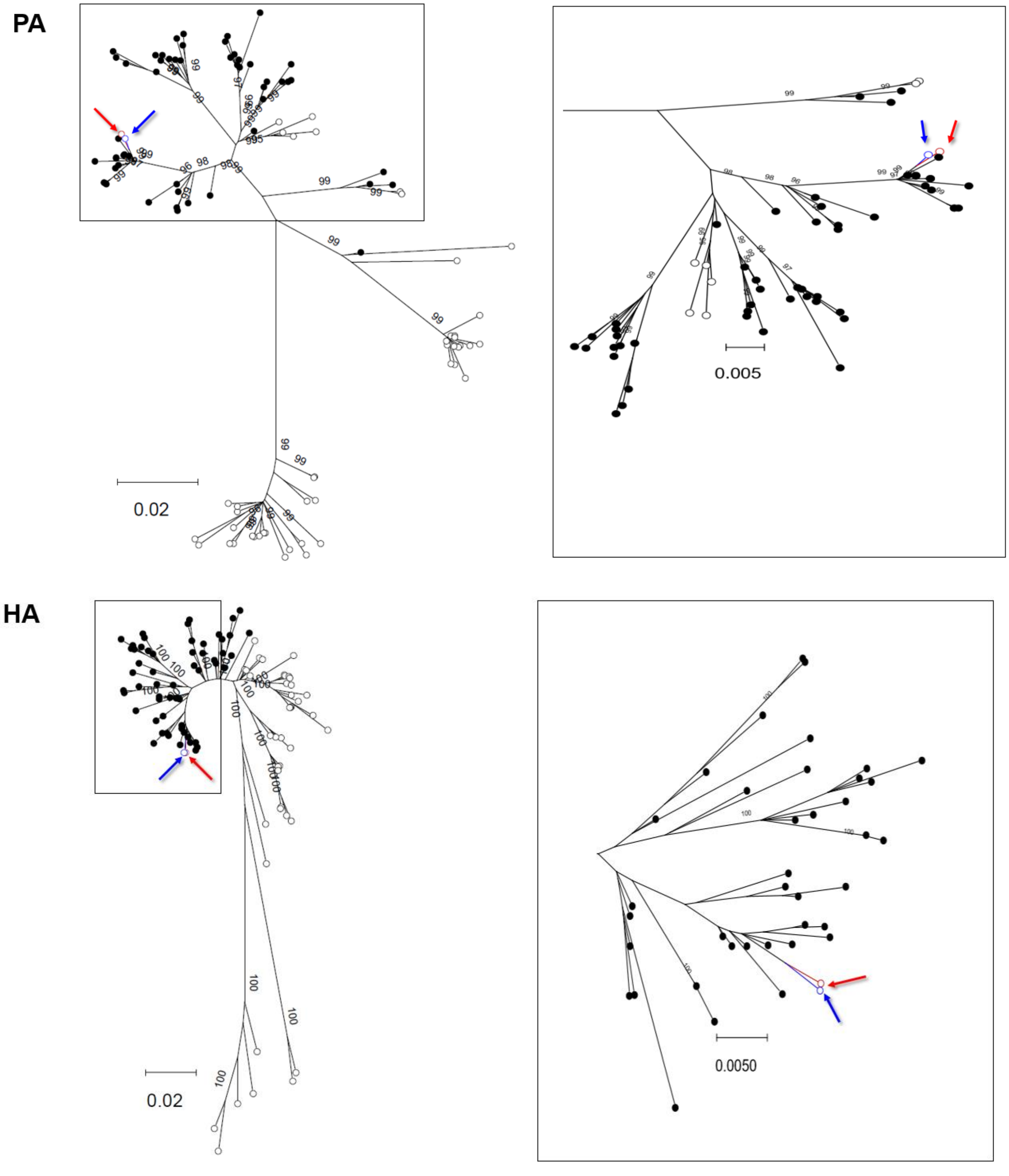
PA
0.02
0.005
HA
0.02
0.0050

NP

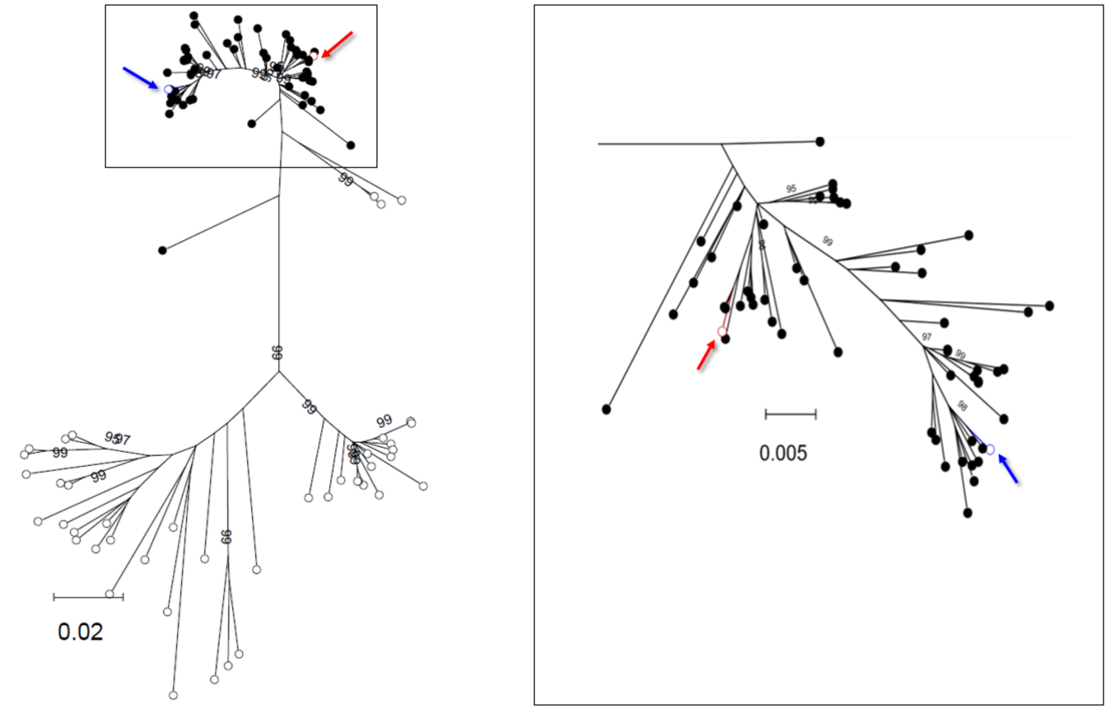

NA

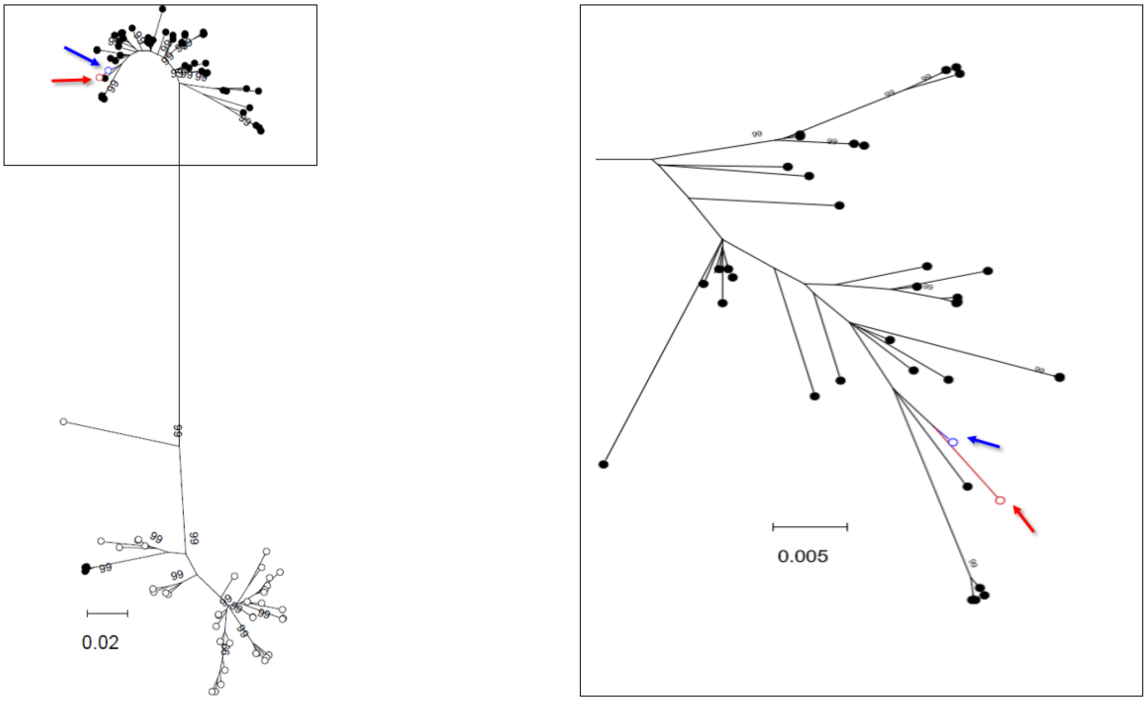

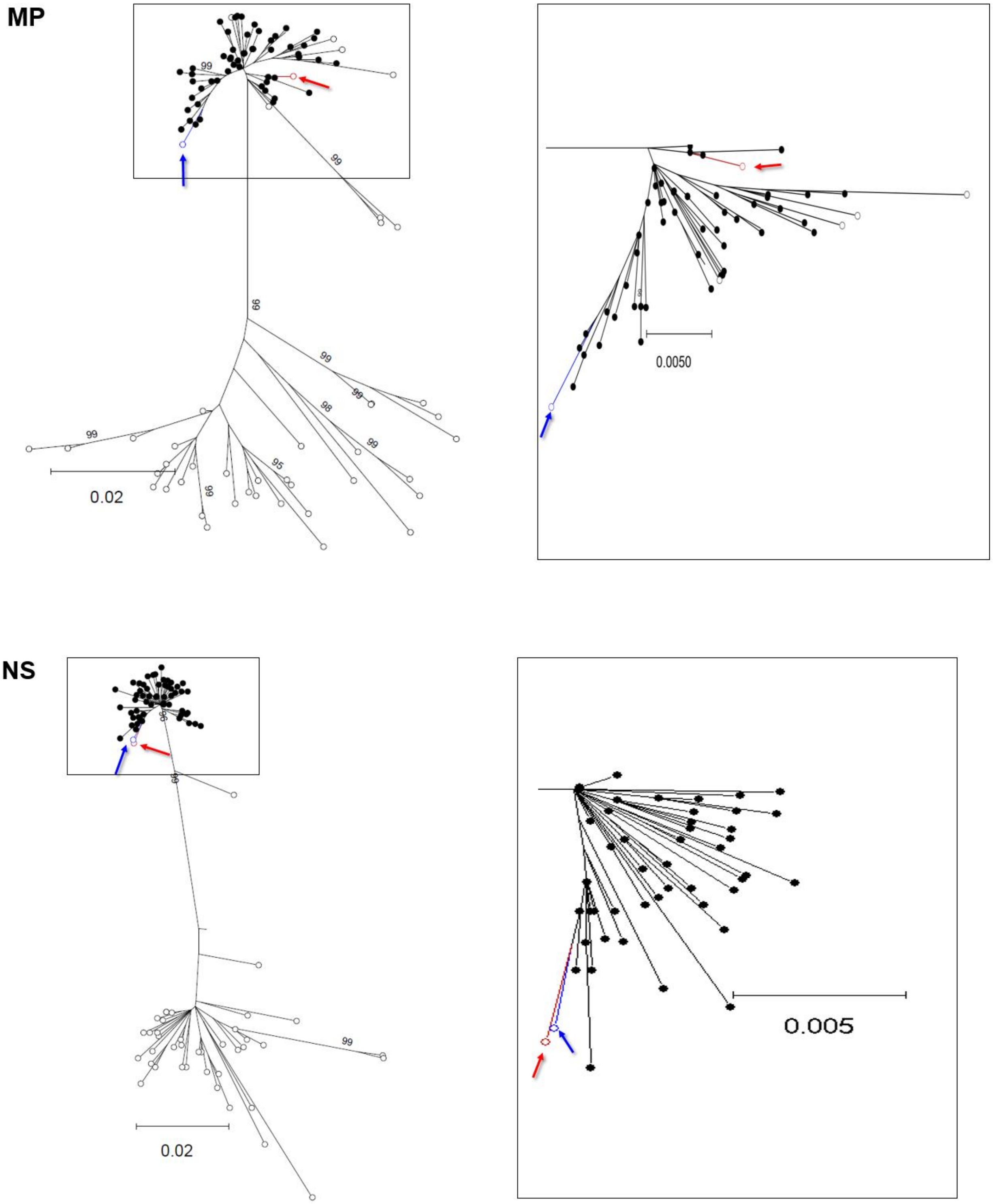

Figure S3. Maximum likelihood phylogenetic trees showing inferred relationships among nucleotide sequences for the complete coding regions of K6 and K17 gene segments. Red open circle indicates K6-H6N5 and blue open circle indicates K17. These

are highlighted with red and blue arrows used for K6 and K17, respectively. Closed and open circles indicate lineages of North America and Eurasia, respectively.

A.

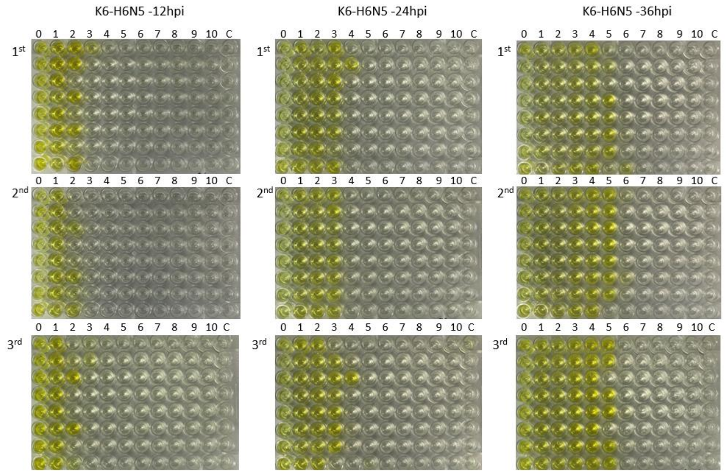

B.

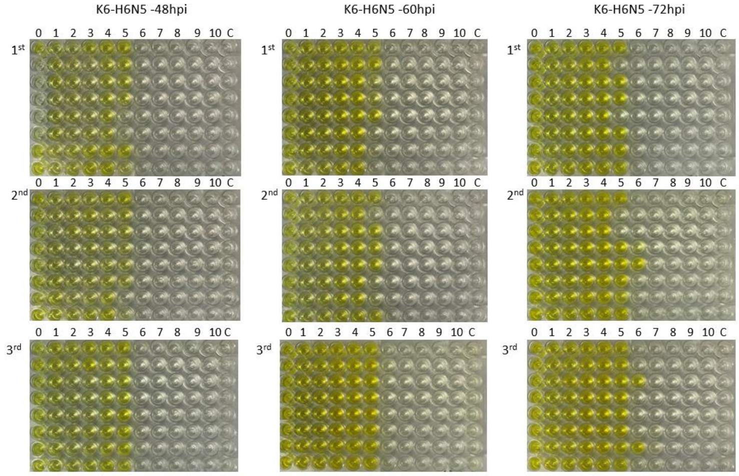

C.

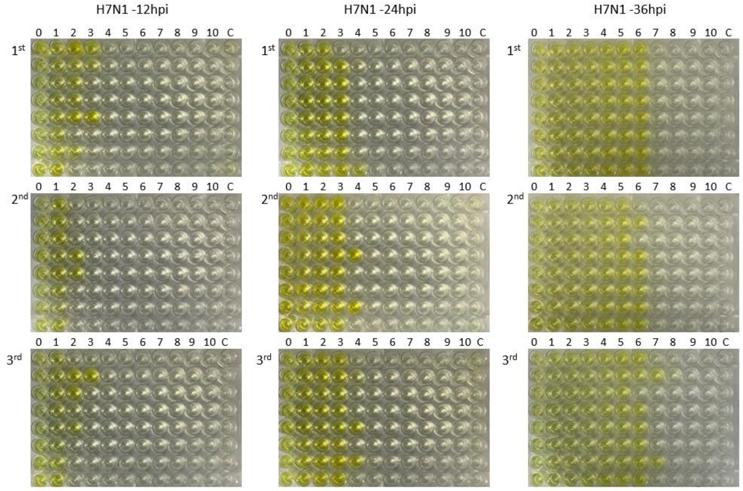

D.

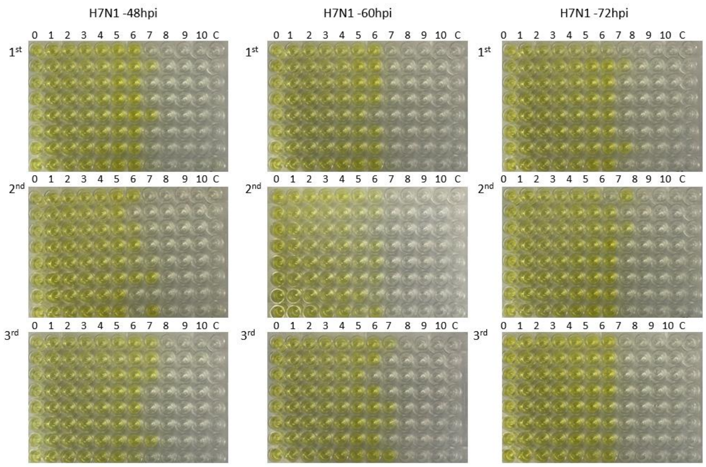
H7N1 -48hpi
H7N1 -60hpi
H7N1 -72hpi
0 1 2 3 4 5 6 7 8 9 10 C
1st
2nd
3rd

E.

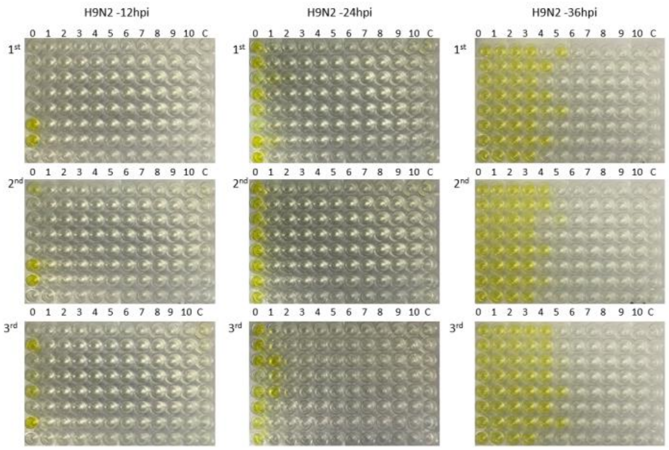
H9N2 -12hpi
H9N2 -24hpi
H9N2 -36hpi
0 1 2 3 4 5 6 7 8 9 10 C
1st
2nd
3rd

F.

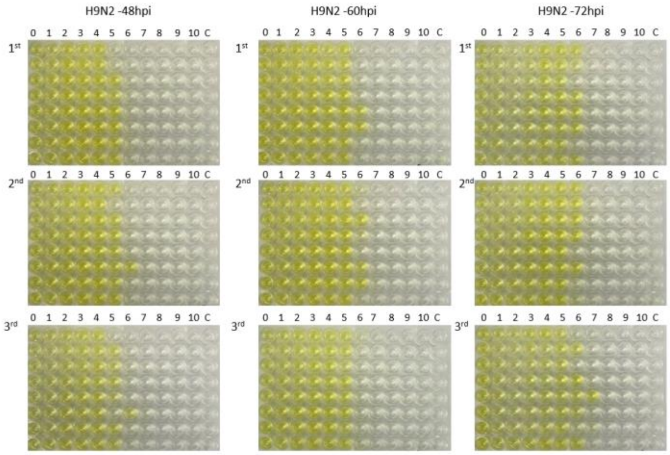
H9N2 -48hpi
H9N2 -60hpi
H9N2 -72hpi
0 1 2 3 4 5 6 7 8 9 10 C
1st
2nd
3rd

G.

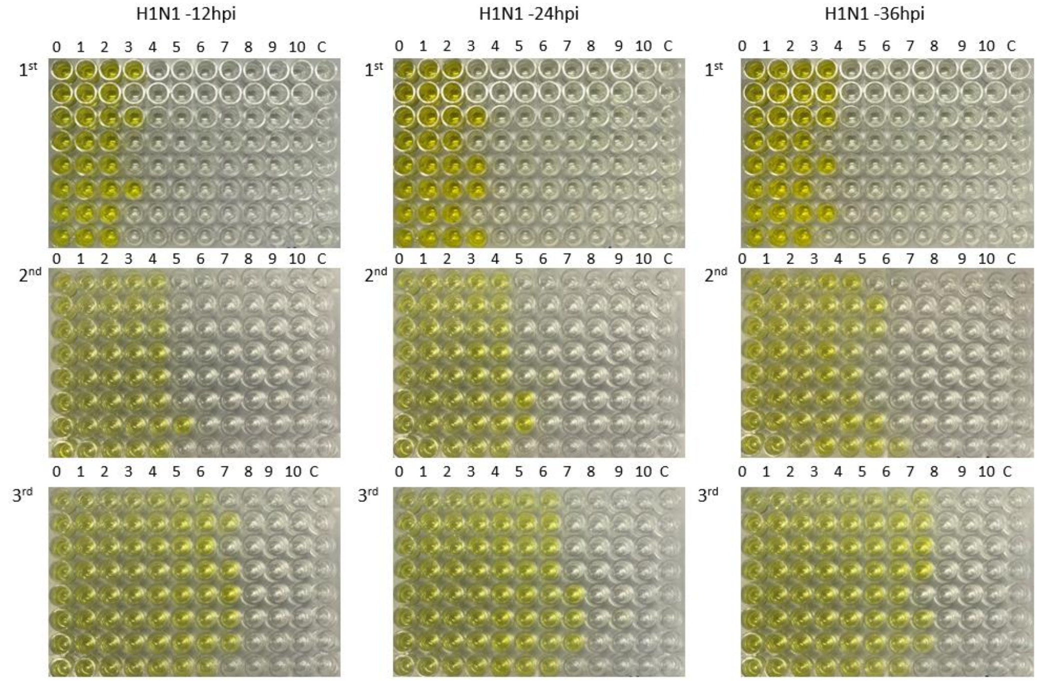

.

H.

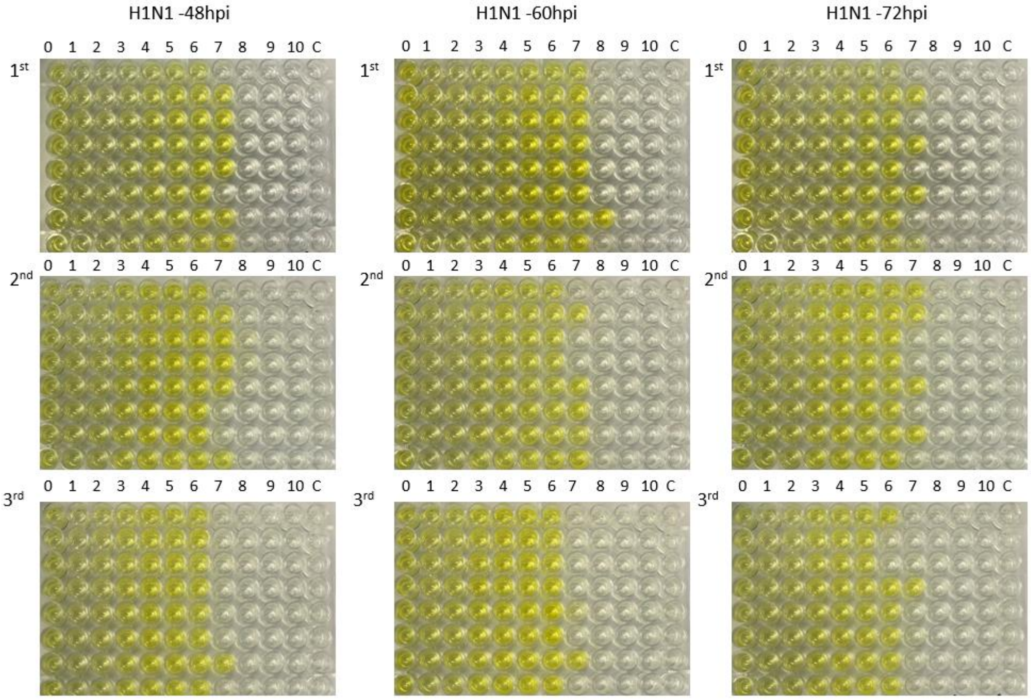

.

Figure S4. Raw ELISA data to conduct TCID$_{50}$ assay. Viruses were inoculated into MDCK monolayers at a multiplicity of infection (MOI) of 0.01. The supernatant were collected at different time point: 12, 24, 36, 48, 60 and 72 hours post of infection (hpi), respectively. The viral supernantant of different viruses (A,B - K6 (H6N5); C,D - H7N1; E,F - H9N2; G,H – H1N1) was serial 10-fold diluted and added to MDCK cell. After 3

days inoculation, medium was removed and cells was washed with PBS then fixed with 80% acetone followed by blocking with 5% non-fat milk. Cells was continuously washed with PBS-T (0.01 Tween 20) and reacted with 0.1 μg/well of anti-influenza nucleoprotein (Medix Biochemica, Finland). Subsequently, secondary mAb in the form of horseradish peroxidase (HRP)-conjugated rabbit anti-mouse IgG (Abcam, Cambridge, UK) was added to each well according to the manufacture's protocol. Washing with PBS-T was performed five times to remove nonspecific binding and 3,3′,5,5′-tetra methyl benzidine (Sigma-Aldrich) substrate solution was added. The reaction was stop by 0.18M H_2SO_4 and the OD value at 450nm was observed. $TCID_{50}$ titer was calculated. 0-10: 10^0-10^{10} folds dilution of stock, C- mock-infection.

A.

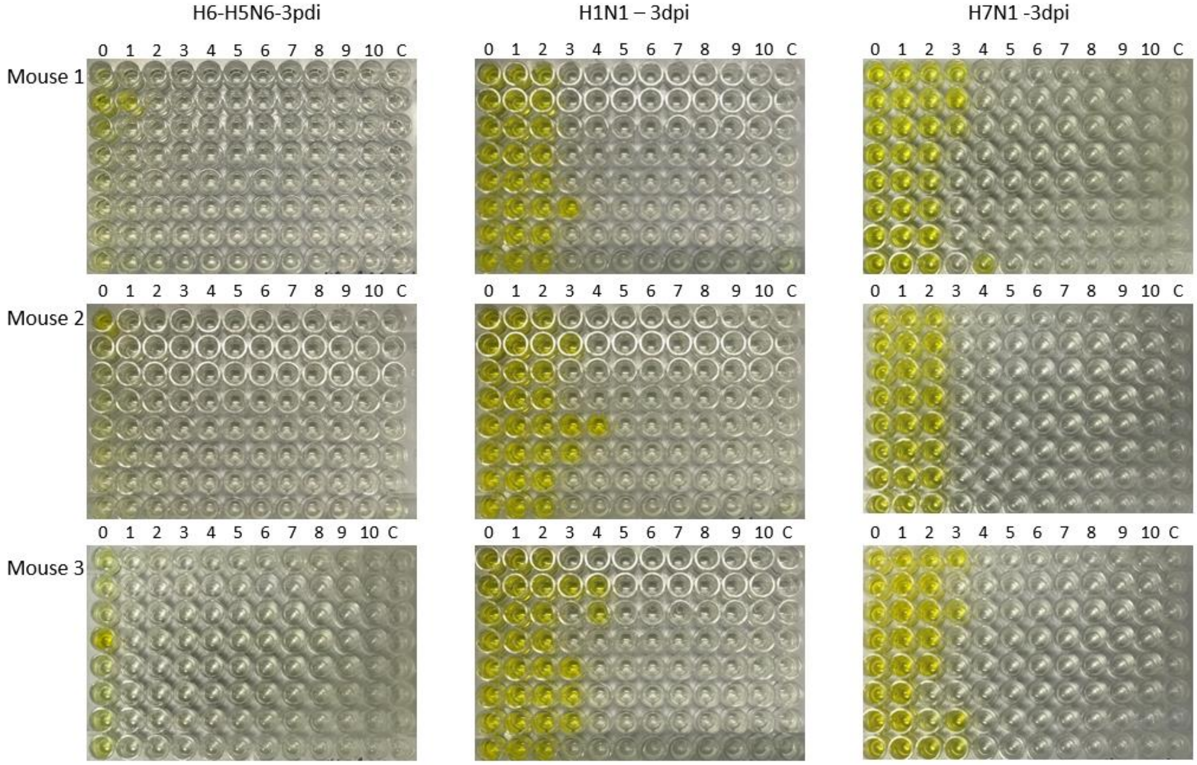
H6-H5N6-3pdi
H1N1 – 3dpi
H7N1 -3dpi
0 1 2 3 4 5 6 7 8 9 10 C
Mouse 1
Mouse 2
Mouse 3

B.

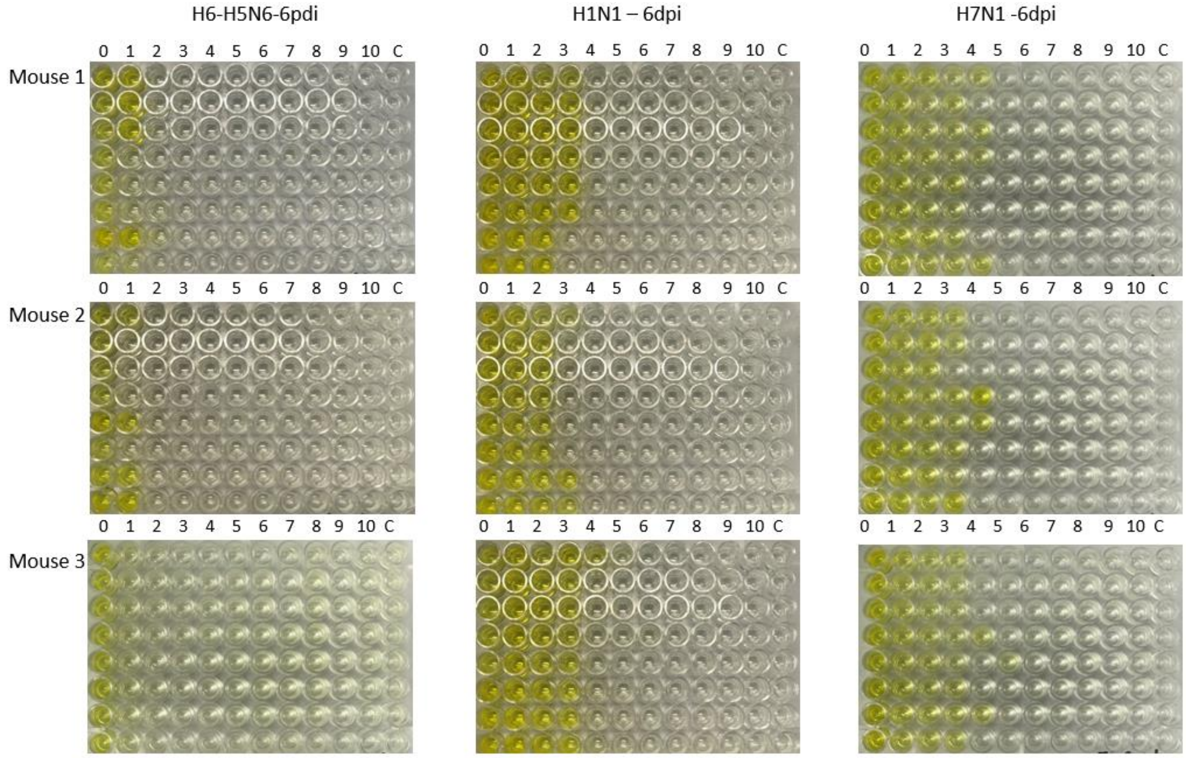
H6-H5N6-6pdi
H1N1 – 6dpi
H7N1 -6dpi
0 1 2 3 4 5 6 7 8 9 10 C
Mouse 1
Mouse 2
Mouse 3

C.

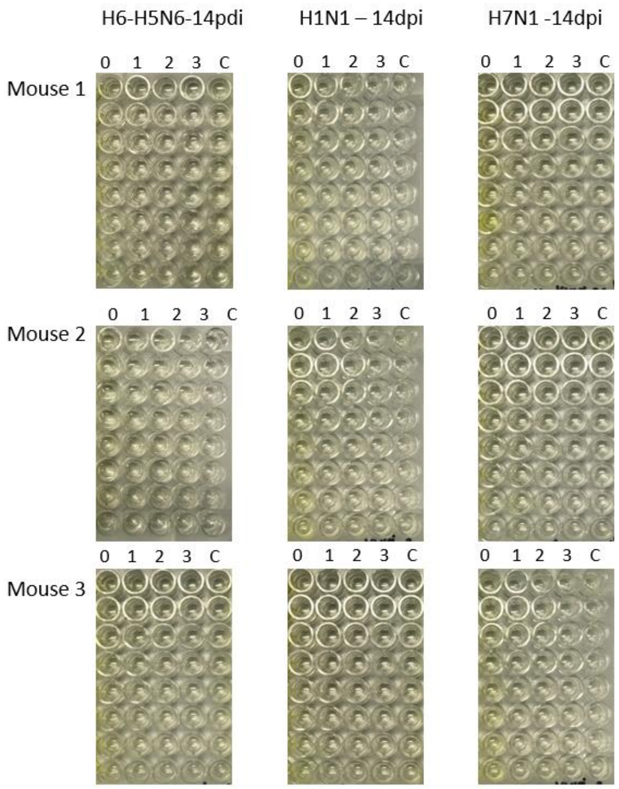

Figure S5. Raw ELISA data to conduct $TCID_{50}$ assay to measure virus K6-H6N5, H1N1, H7N1 titer in lung at day 3 (A); day 6 (B); day 14 (C) post infected. 0,10^0- fold dilution of stock; 1, 10^1- fold dilution of stock; 2, 10^2- fold dilution of stock; 3, 10^3- fold dilution of stock; 4, 10^4- fold dilution of stock; 5,10^5- fold dilution of stock; 6, 10^6- fold dilution of stock; 7, 10^7- fold dilution of stock; 8, 10^8- fold dilution of stock; 9, 10^9- fold dilution of stock; 10, 10^{10}- fold dilution of stock; C, mock-infection;

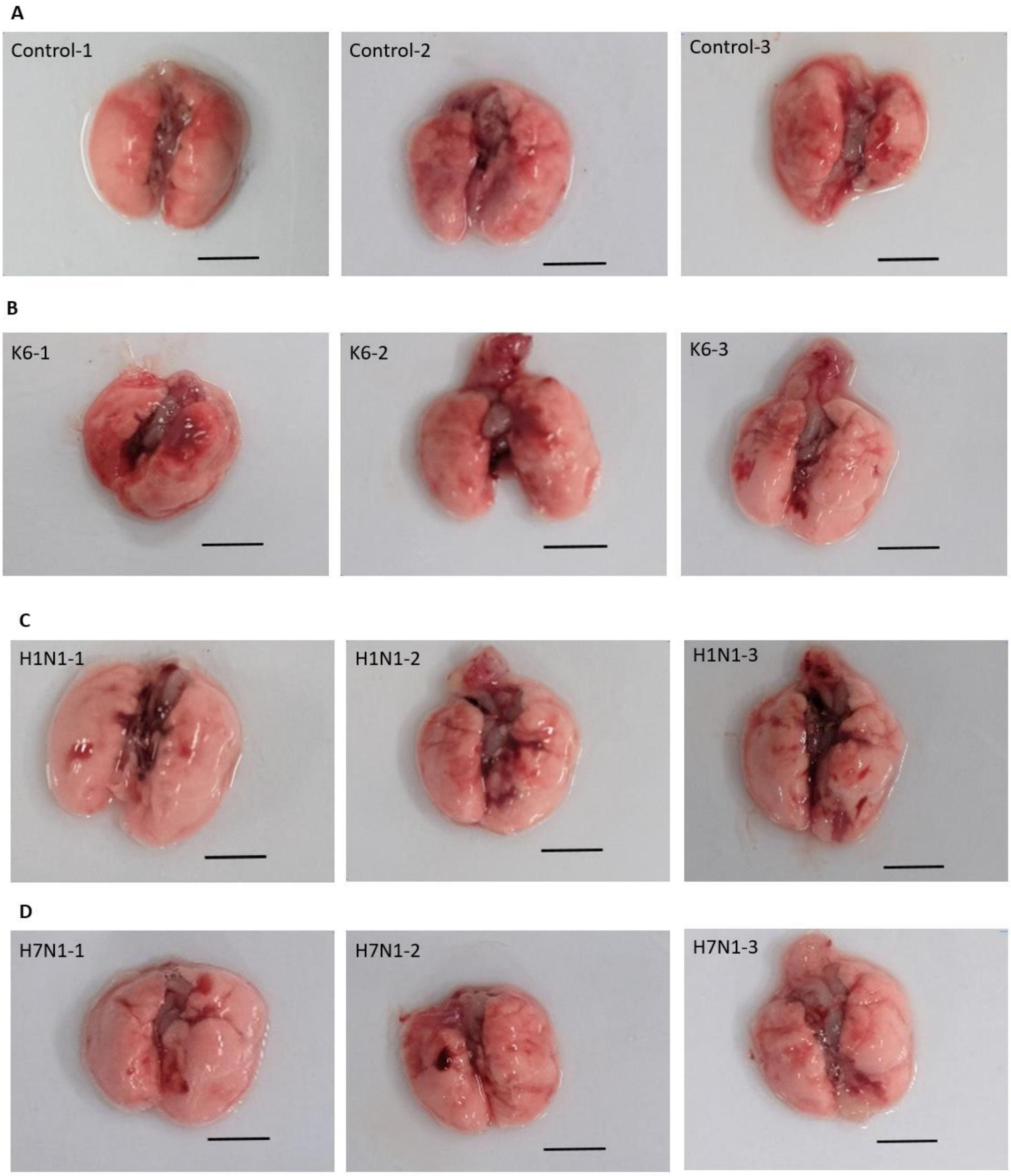

Figure S6. Lungs from (A) uninfected control; (B) K6-H6N5; (C) H7N1; (D) H7N1 - infected mouse at 6 dpi. Scale bar: 0.5cm

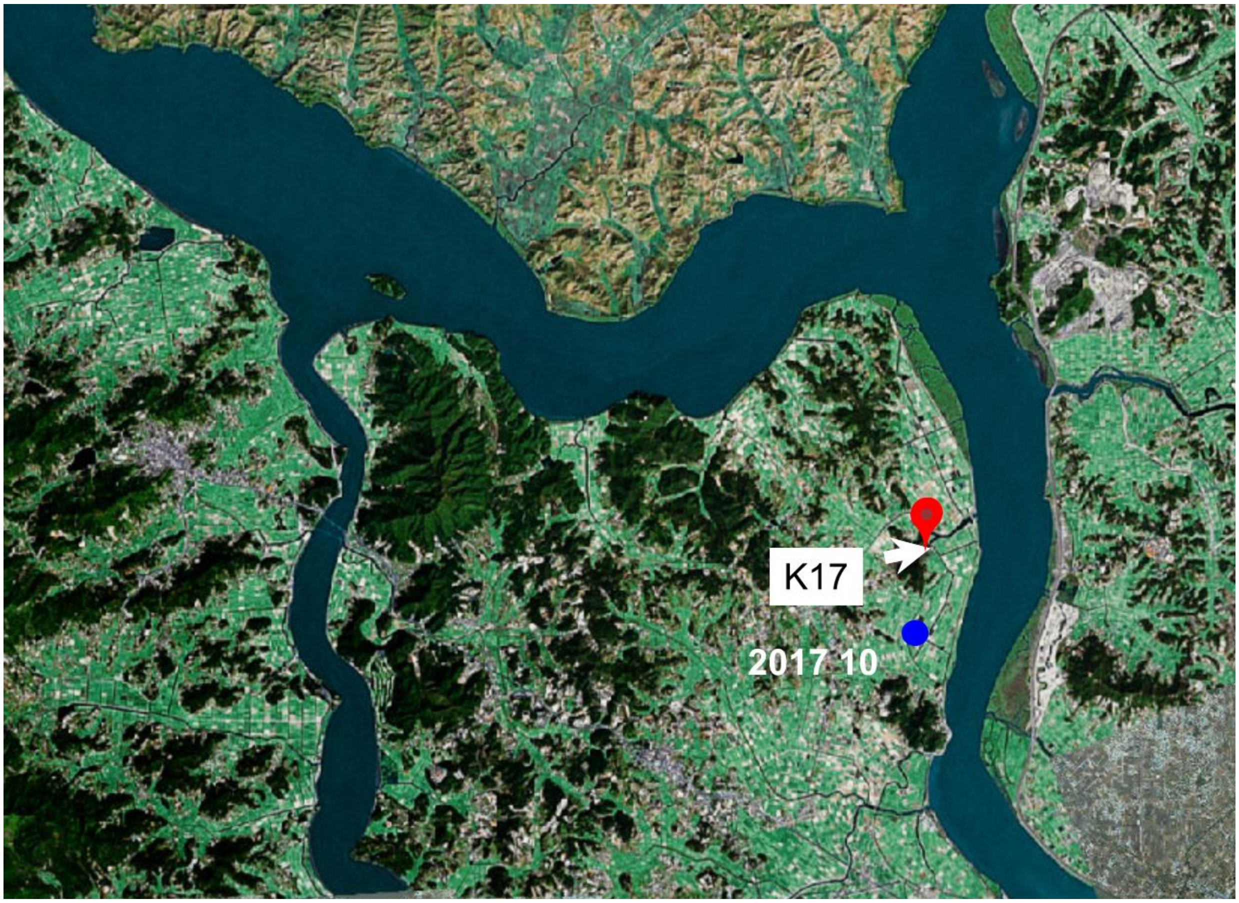

Figure S7. Location map of mandarin duck marked with satellite transmitters in Korea in October in 2017. White arrow indicates the isolation place of A/Aix galericulata/South Korea/K17-1638-5/2017(H6N5) and blue dot indicates the position of mandarin duck in October 2017. Map was retrieved from National Institute of Biological Resources, Korea.

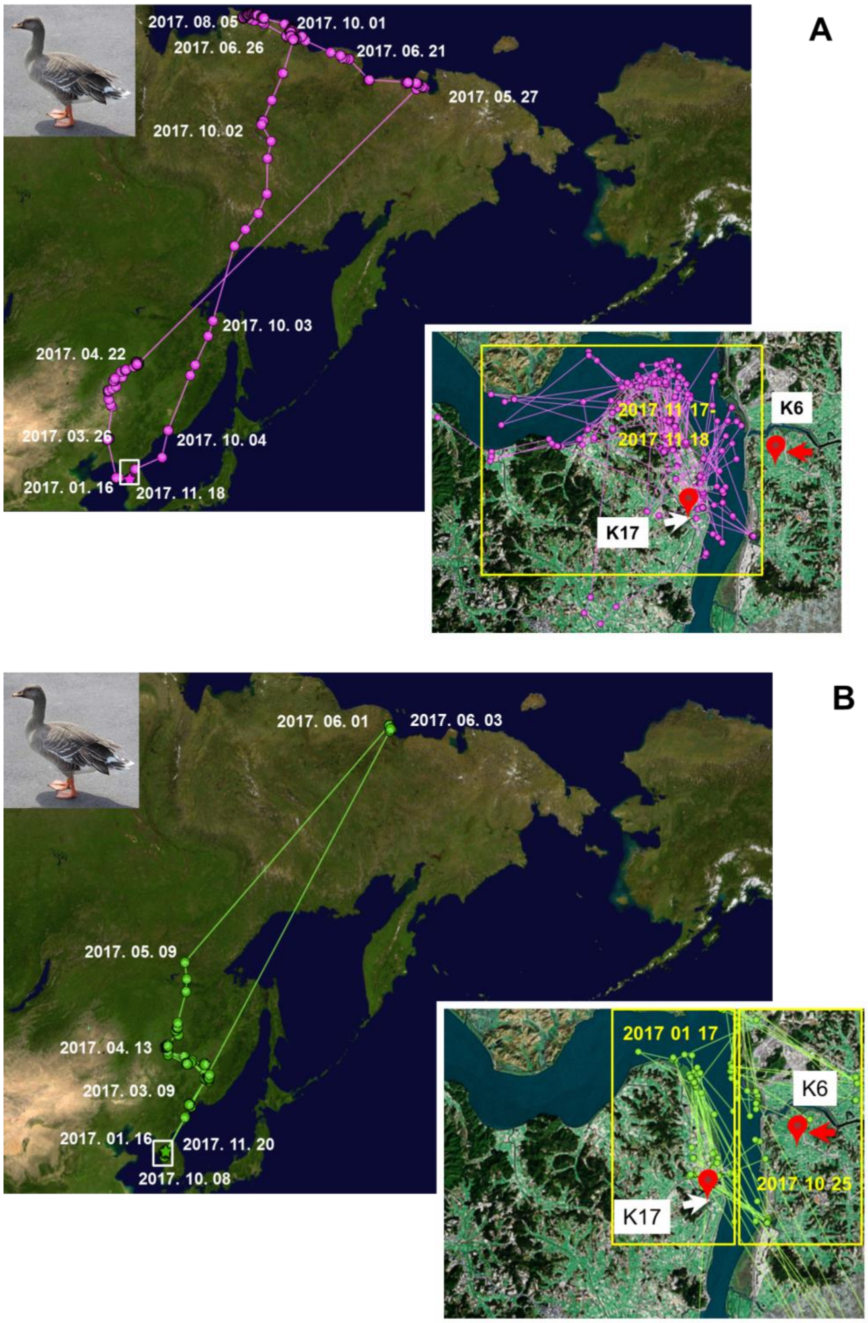

Figure S8. Spatial distribution patterns of Bean geese during Jan 2017- Nov 2017. Two satellite tracking devices (A, **br1691**; B, **br1692**) are shown. Red and white arrows indicate the isolated places of K6 and K17, respectively. Maps were retrieved from National Institute of Biological Resources, Korea. Date of movement (yellow color) is marked in yellow lined box in insert.
